# *In Vivo* Multimodal Self-Immolative Prodrug Nanomicelle-Hydrogel Modulate Key Intratumoral and Metastatic Genomic Signaling Pathways in Pancreatic Cancer

**DOI:** 10.1021/acsnanomed.5c00174

**Published:** 2026-07-07

**Authors:** João M. J. M. Ravasco, Jhenifer Oliveira, João Conniot, Bárbara B. Mendes, Catarina Leal, Susete N. Fernandes, Carlos A. M. Afonso, João Conde

**Affiliations:** † NOVA Medical School|Faculdade de Ciências Médicas, 50106 NMS|FCM, Universidade NOVA de Lisboa, 1169-056 Lisbon, Portugal; ‡ Comprehensive Health Research Centre (CHRC), NOVA Medical School|Faculdade de Ciências Médicas, NMS|FCM, Universidade NOVA de Lisboa, 1169-056 Lisbon, Portugal; § i3N/CENIMAT, Department of Materials Science, NOVA School of Science and Technology, Universidade NOVA de Lisboa, Campus de Caparica, 2829-516 Caparica, Portugal; ∥ Departamento de Física, Instituto Superior de Engenharia de Lisboa, Instituto Politécnico de Lisboa, 1549-020 Lisboa, Portugal; ⊥ Instituto de Investigação do Medicamento (iMed), Faculdade de Farmácia, 70880 Universidade de Lisboa, Av. Prof. Gama Pinto, 1649-003 Lisboa, Portugal

**Keywords:** micelles, pancreatic ductal adenocarcinoma, gemcitabine, paclitaxel, hydrogel, local delivery, biomarker, therapeutic target

## Abstract

To address the critical challenge of high-lethality pancreatic ductal adenocarcinoma and poor response to conventional chemotherapies, this study introduces a self-immolative micelle encapsulating paclitaxel (PTX) or gemcitabine (GEM), two frontline chemotherapeutic agents. These chemotherapeutic agents present a 2.3-fold reduction for GEM and a 61.7-fold reduction for PTX in IC_50_ values when conjugated to the micelle system, compared to their free drug counterparts. Through longitudinal bioluminescence imaging, we observed a significant reduction in peritoneal tumor implants versus metastatic tumor deposits growth and a decrease in metastatic spread in the GEM- and GEM+PTX-micelle (micelle@GEM and micelle@GEM+PTX)-treated groups compared with the free drug formulations. Gene expression analysis of treated tumors revealed interesting oncogenic pathways, including PI3K-Akt and MAPK signaling, suggesting a targeted action at the molecular level. Our results identified the dual roles of *PSMA1* and *UBE2C* as poor prognostic markers of pancreatic cancer progression and as candidate genes of interest, with key roles in the ubiquitination and proteasome degradation pathways in the pathophysiology of the disease. These data suggest that encapsulation of PTX and GEM in these micelles embedded within a hydrogel could alter the pharmacokinetics and pharmacodynamics of these agents, thereby modulating their interactions with cellular targets. Such targeted approaches could transform the treatment of pancreatic cancer by providing tailored therapies based on the unique genomic landscape of an individual’s tumor, thus optimizing the clinical outcomes.

## Graphical abstract

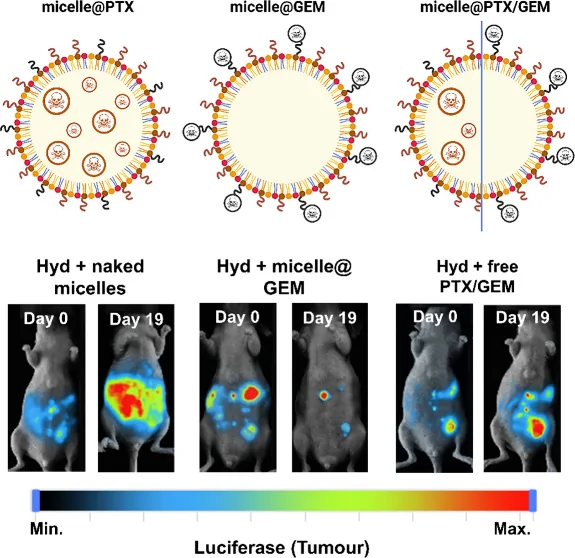

## Introduction

Pancreatic cancer remains both one of the leading causes of cancer mortality, and one of the most lethal malignancies.^1–3^ Although less frequent than lung, breast, colorectal, and prostate malignancies, it was the third highest cause of cancer-related death in 2021.^1,3–5^ This discrepancy is largely attributed to a late diagnosis, conferring a rate of 51% distant dissemination at diagnosis with a 5-year relative survival of only 3.2% (Surveillance, Epidemiology, and End Results 21, 2015–2021).^6^ The standard of care for metastatic disease remains cytotoxic therapy, and a variety of novel therapeutic approaches are currently under active investigation.^7,8^ Systemic chemotherapy (CT) remains the mainstay in both first-line (1 L) and second-line and beyond (2 L + ) regimens in patients with locally advanced and metastatic pancreatic ductal adenocarcinoma (PDAC) without actionable genomic alterations.^9–12^ Gemcitabine (GEM) with nab-paclitaxel (PTX), with or without cisplatin, or fluoropyrimidine regimens are preferred in both 1 and 2 L + settings for patients with good to intermediate performance scores, but offer limited benefit and high systemic toxicity.^13^ Thus, patients inevitably progress and have limited access to high-value 2 L + therapies, further contributing to the low 5-year survival. Recently, NALIRIFOX (i.e., liposomal irinotecan, 5-fluorouracil, leucovorin, and oxaliplatin) was approved for first-line therapy of eligible patients with metastatic PDAC. NAPOLI 3 trial showed that NALIRIFOX improved survival outcomes compared with the established first-line treatment combination of gemcitabine plus nab-paclitaxel.^14,15^ Among many factors, the dense desmoplastic pancreatic tumor microenvironment (TME) has shown to play an important role in the failure of most therapeutic strategies, by promoting a particular low drug accumulation at the tumor site and for a short period of time.^10,16,17^ These factors limiting the delivery and efficacy of systemic therapy, highlight a striking need to develop more effective therapeutic strategies.^7,8^

Nanomedicines are revolutionizing the standardized means of delivering therapeutic cargo.^18,19^ While systemic chemotherapy and radiotherapy are the main conventional therapies in oncology. Inadequate drug bioavailability, cellular selectivity, and development of multidrug resistance are common causes of limited efficacy and high systemic toxicity.^20^ Nanomedicine has demonstrated numerically higher efficacy and safety compared to standard systemic free drug CT by overcoming some of these challenges, namely: (i) avoiding side and off-target effects by active biomarker targeting or EPR at the site of interest; (ii) enhancing intracellular uptake in cancer and metastatic cells; (iii) improving controlled payload pharmacokinetics and pharmacodynamics; and (iv) protecting the encapsulated payload from degradation or rapid clearance.^21–23^ The introduction of immunotherapy and targeted therapies has significantly improved the outcomes of many patient populations across several solid and hematologic tumors; however, these have shown limited clinical benefit in pancreatic cancer.^4,15,24,25^ While limited, approved nanoparticles (NP) therapies for PDAC (e.g., liposomal irinotecan) highlight the importance of leveraging nanosystems to improve the efficacy and safety of standard chemotherapies.^26^ Alternative delivery routes and tuning key components that can exert synergistic activity within the tumor (e.g., MDR reversal and redox modulation) to achieve sustained drug release, improve response duration, and reduce off-target toxicity.^27,28^

Herein, we developed a hyaluronic acid (HA)-based hydrogel containing tailor-made tocopherol-based micelles for intraperitoneal administration. The nanosystem was designed to encapsulate lipophilic payloads (e.g., rhodamine, PTX) and/or append and protect the hydrophilic PDAC mainstay drug GEM (Figure 1a and b). Notably, GEM presents particularly marked hydrophilicity, serum instability and off-target effects. These limitations were bypassed by surface linkage to the micelle system via a responsive stable linker, which allows near quantitative and chemically intact GEM release inside cancer cells through a glutathione (GSH) stimulus. Hydrogels, particularly HA-based hydrogels, offer a biocompatible, biodegradable, and customizable platform for controlled and targeted drug delivery, which enhances therapeutic efficacy while minimizing side effects.^29^ The *in vitro* cytotoxic evaluation of micelles carrying GEM (micelle@GEM) and micelles carrying PTX (micelle@PTX) showed a significant reduction in pancreatic cancer cell metabolic activity by 50- and 100-fold, respectively, compared with the free drug combination. The multimodal system showed modulation of key genomic pancreatic cancer signaling pathways (e.g., PI3K-Akt and MAPK) in both peritoneal tumor implants versus metastatic tumor deposits, as well as significant enrichment of gene sets in ubiquitination, proteasome degradation, and MHC-mediated antigen processing, with a deep and durable anticancer response after a single administration of HA-based hydrogel containing drug-loaded micelles in a PDAC metastatic model.

**Figure 1:**
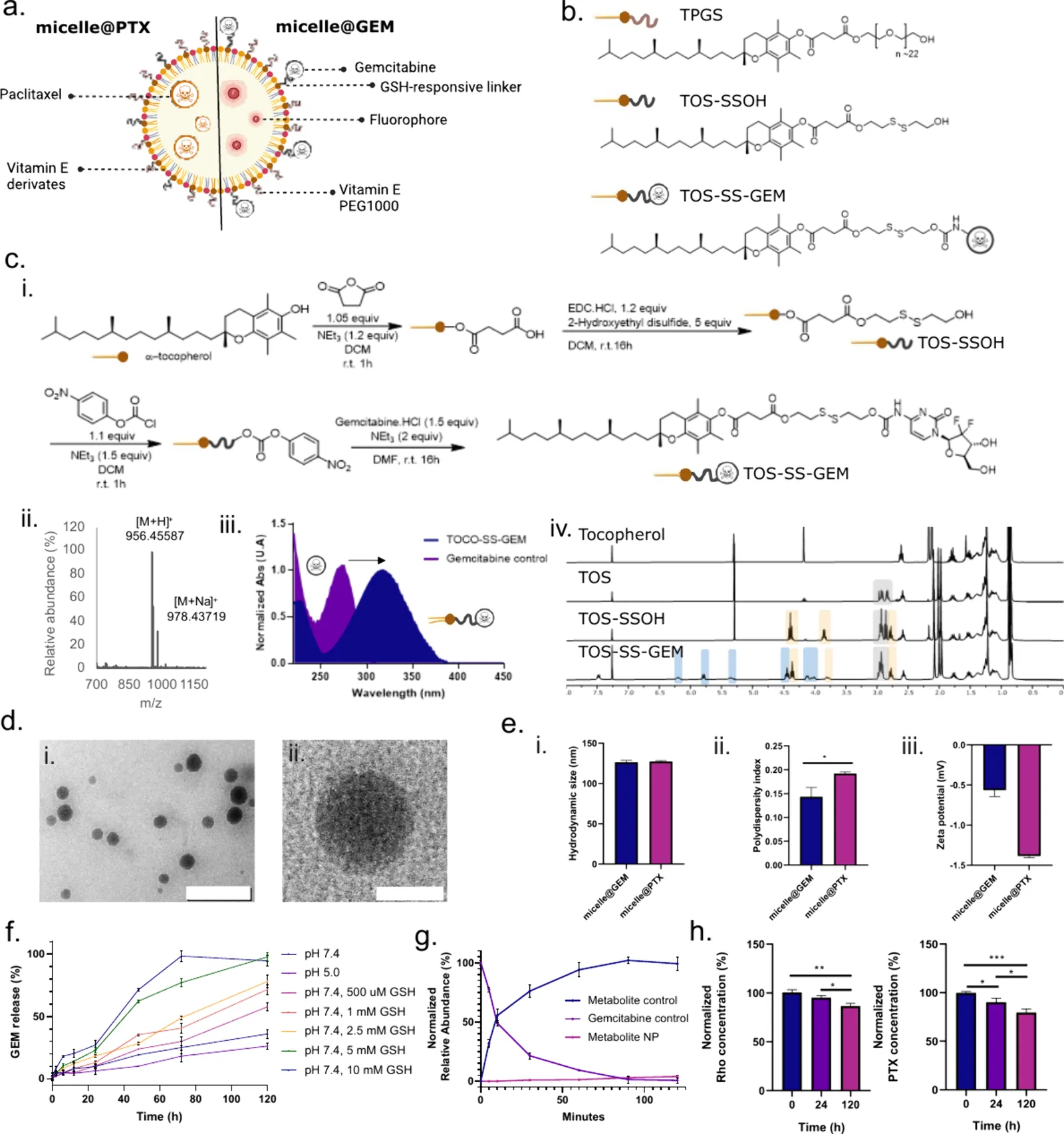
Production and characterization of micelles containing paclitaxel (micelle@PTX) or gemcitabine (micelle@GEM). (a) Schematic representation of vitamin-E-based micelles for triggerable multimodal pancreatic ductal adenocarcinoma (PDAC) nanotherapy. The nanoparticles are core–shell polymeric micelle: PTX resides in the hydrophobic tocopherol-rich core; GEM is redox-cleavably tethered to the corona (TPGS/TOS derivatives). (b) Schematic representation of the main micellar components. (c) (i) Chemical construction of the main micellar components, including TOS, TOS-SSOH, and TOS-SS-GEM.( ii) LC-HRMS, (iii) UV–vis, and (iv) C NMR spectra of the GEM product site, specifically modified at the −NH_2_ moiety. (d) (i, ii) TEM images of micelle@GEM. Scale: (d) (i) 500 nm, (d) (ii) 50 nm. (e) (i) Hydrodynamic size by DLS, (ii) polydispersity index, *N* = 3 independent biological experiments, mean ± SD, *p < 0.05, two-tailed unpaired *t* test, and (iii) charge based on zeta potential of micelle@PTX and micelle@GEM. (f) LC-HRMS study of TOS-SS-GEM and micelle@GEM at different glutathione (GSH) concentrations and pH. (g) Plasma metabolism by CDA. (h) Profile of the release of encapsulated agents (Rhodamine or PTX) from the produced micelles. *N* = 3 independent biological experiments, mean ± SD, *p < 0.05, **p < 0.01, ***p < 0.001, ordinary ANOVA test with Tukey’s multiple comparisons test.

## Experimental Section

### Chemical Synthesis, Nanoformulation, and Hydrogels

This information, as well as a detailed analysis of micelles and hydrogels, are extensively documented in the Supporting Information. Briefly, the production and characterization of nanoformulations involves a systematic approach, starting with general synthetic procedures to create a series of novel compounds. These compounds included α-tocopherol succinate (TOS), TOS-SSOH, Toco_OSSOH_PNB, and TOS-SS-GEM, with a gradual increase in specificity. To ensure the purity and structure of the synthesized compounds, liquid nuclear magnetic resonance spectroscopy (NMR) and liquid chromatography–mass spectrometry (LC-MS) were employed. Further, the research extended to the fabrication and detailed characterization of micelles and hydrogels. This involved preparing micelle@GEM and rhodamine-loaded micelle@GEM, followed by an assessment of their surface morphology, size, size distribution, and particle stability, see SI section 2. The release mechanism of these nanoformulations was analyzed using LC-HRMS for GSH-triggered release and CDA metabolism, along with UV–vis quantification of the cargo-loaded micelles. This provided a thorough evaluation of micelle characterization, highlighting the effectiveness and potential applications of these nanoformulations in targeted drug delivery. In combination groups, micelle@PTX and micelle@GEM were coembedded in the same HA hydrogel; no dual-loaded single micelle was used.

### Cell Culture

A human pancreatic adenocarcinoma cell line stably expressing luciferase (BxPC-3-Luc) was acquired from Japanese Collection of Research Bioresources Cell Bank (JCBR) with a catalog number #JCBR1448 (TeBU-BIO). BxPC3 derived from a 61-year-old female with a primary adenocarcinoma of the pancreas. BxPC-3-Luc was cultured in RPMI 1640 (Gibco) supplemented with 10% fetal bovine serum (FBS, Gibco) and 1% penicillin/streptomycin solution (PEN/STREP, Gibco) at 37 °C and 5% CO_2_. Cells were trypsinised with 0.25% trypsin-EDTA (Gibco) and resuspended in supplemented culture media for further experimental analysis.

### Metabolic Activity

The metabolic activity of BxPC-3-Luc cells was evaluated at 24h, 48h, and 72h after NP incubation using the [3-(4,5-Dimethylthiazol-2-yl)-2,5-Diphenyltetrazolium bromide assay (MTT, 0261006, Tebu-bio). BxPC3 cells were seeded at 5000 cells per well in a 96 well plate. Upon 24h of culture, the different micelle conditions (i.e., micelle@linker, micelle@PTX, micelle@GEM, and micelle@PTX+GEM) and free drugs (i.e., free GEM, free PTX, and free PTX + GEM) were added to each well with serial dilutions ranging from 0.00001 to 50 μM. At 24h, 48 h, or 72 h, the medium was removed and 100 μL of fresh medium was added. MTT solution (12 mM in PBS) was added to each well and incubated at 37 °C for 2 h. Dimethyl sulfoxide (DMSO, Sigma) was then added to each well to ensure the dissolution of formazan crystals and incubated for an additional 10 min at room temperature (RT). The optical density (OD) was measured at 540 nm using a microplate reader (Synergy HT, Marshall Scientific). The combination arm was designed as an equi-effect comparison, with each agent administered at its independently determined IC_50_. This design follows established frameworks for synergy assessment and is not a pharmacokinetic equi-mass comparison. Conclusions drawn from this design reflect effect-based benchmarking, not absolute dose-normalized pharmacokinetic equivalence.

### NPs Internalization

BxPC-3-Luc cells were seeded at 25000 cells per well in 24 well plate. Micelles@Rho (1 μM) was added to BxPC3 cells, which were then collected 0.5h, 4h, 6h, 24h, 48h, and 72h after micelles incubation. Cells were fixed with paraformaldehyde (PFA, 4 wt % in PBS) for 20 min at room temperature (RT), washed with PBS, and resuspended in FACS buffer (2 vol % FBS in PBS). Cells were acquired on a BD FACSAria III Cell sorter (BD Biosciences), and the results are presented as % parent or mean fluorescence intensity (a.u.).

### Confocal Microscopy

BxPC-3-Luc cells were seeded at 25000 cells per well in a 24 well plate on round coverslips (13 mm). After incubation with micelles@Rho (1 μM) for 24 h, cells were washed with PBS and fixed with a solution of PFA (4 wt % in PBS) and sucrose (2 wt % in PBS) for 15 min at 37 °C. After washing, the cells were permeabilised, washed with PBS, and incubated with 4, 6-diamidino-2-phenylindole (DAPI, 300 nM) for 10 min. The cells were washed with PBS and mounted on a glass slide with a coverslip using a drop of the ProLong Glass Antifade Mountant. The prepared slides were examined under a confocal laser-scanning microscope 710 (Zeiss).

### Cell Cycle

BxPC-3-Luc human pancreatic cancer cells were plated in 24 well-plates, 30000 cells/well. After 24h, cells were treated with six different formulations: free gemcitabine (GEM), free paclitaxel (PTX), micelle@linker, micelle@GEM, micelle@PTX and micelle@PTX+GEM using IC50 doses. After 72h of incubation, the cells were harvested and fixed using cold 70% ethanol. To stain DNA, the cells were incubated with a solution of propidium iodide (0.05 mg/mL) and RNase (0.02 mg/mL) in PBS for 30 min. The samples were analyzed using a FACS Canto cytometer. Data were analyzed using flowjo v10.10.0 software using the gating strategy explained at Figure S21. The phases were identified according to the mean fluorescence intensities of the propidium iodide channel using the Dean-Jett-Fox plugin. The applied plugin determines the percentage of cells in each phase, including sub-G0 to super-G2, while considering that some cells may overlap between phases. The probabilities are adjusted to preserve the proportion between the phases.

### Animal Studies

Athymic BALB/c female nude mice (6 weeks old, average weight = 20 g) were purchased from Charles River Laboratories (France) and acclimatized for 1 week before the experiments. Animals were kept under aseptic conditions with light/dark cycles of 12h. Water and standard pellet food were provided *ad libitum*. The animal procedures were performed under isoflurane anesthesia (IsoFlo 100%p/p).

### Biodistribution Studies

Mice (n = 5) received 500 μL/mouse of 4% hyaluronic acid hydrogel containing rhodamine-labeled micelles by intraperitoneal injection with a 23G needle, as following Guidelines of NIH office of Animal Care and Use. Whole-body fluorescence was measured at 540 nm after 0, 0.5, 1, 4, 9, 24, and 48 h using Newton FT500 imaging system (Vilber). Fluorescence images were acquired on a Newton FT500 (Vilber) using a rhodamine filter set (Ex 535–560 nm/Em 580–650 nm), fixed exposure, f/stop and binning; raw 16-bit histograms were checked to exclude saturation. At 24h post injection, the organs (liver, spleen, pancreas, heart, lungs, and kidneys) were harvested, and organ fluorescence was measured at 540 nm. *Ex vivo* organ signals are reported as radiant efficiency; %ID/g was not computed because organ weights were not collected.

### PDAC *In Vivo* Model

To evaluate treatment efficacy, BxPC-3-Luc human pancreatic cancer cells (2.4 × 10^6^/mouse) were injected intraperitoneally. BxPC-3-Luc cells were injected intraperitoneally to establish a peritoneal pancreatic carcinomatosis model. In this model, tumor foci located in the peritoneal cavity represent peritoneal tumor implants or dominant peritoneal tumor deposits, not orthotopic pancreatic primary tumors. Organ-associated foci detected in the liver, spleen, pancreas, lungs or peritoneal membrane were therefore described as disseminated or metastatic tumor deposits. Randomization into experimental groups (n = 5) was performed based on baseline bioluminescence signal acquired prior to formulation administration, ensuring equivalent tumor burden distribution across groups. After treatment with 6 different hydrogel-loaded formulations, we assessed tumor progression by evaluating bioluminescence signal and survival analysis, both correlating with the tumor burden. Tumor growth was monitored using bioluminescence imaging. Mice were injected intraperitoneally with D-luciferin (ABP Biosciences, 15 mg/mL in DPBS, 200 μL/mouse) and bioluminescence was measured 1 min later. At day 21 post tumor induction, mice were distributed in 6 groups (n = 5): 1) Hydrogel, 2) Hydrogel with empty micelles, 3) Hydrogel with free PTX+GEM 4) Hydrogel with micelles@PTX, 5) Hydrogel with micelles@GEM, 6) Hydrogel with micelles@PTX+GEM. Mice received 500 μL/mouse of the 4% Hyaluronic acid hydrogel containing the respective treatment (see SI - Dosing rationale section) by intraperitoneal injection with a 23G needle. Bioluminescence measurements were performed on days 0, 2, 5, 8, 12, and 19 post-treatments. At the end point (day 19), mice were euthanised by cervical dislocation. The abdominal wall and organs (the liver, spleen, pancreas, heart, and lungs) were harvested and imaged for bioluminescence. Several tumoral foci identified in the organs and abdominal wall were collected and stored in a lysis buffer containing 1% 2-Mercaptoethanol for RNA extraction. All animal experiments were approved by the Ethical Committee and the Animal Welfare and Ethics Body of Nova Medical School (21_01_ORBEA.4) and followed the Animal Research guidelines of Nova Medical School and of the Directorate-General for Food and Veterinary Medicine. Animals were humanely sacrificed upon meeting predefined end point criteria (see Table S4): 20% loss of body weight or other signs of illness (lethargy or a body condition score ≤ 2), abdominal distension from ascites fluid, tumor ulceration or compassionate euthanasia when tumor volume >1000 mm^3^ or when total tumor bioluminescence reached ≥ 1.8 × 10^8^ ph/s/sr, because this value corresponded to the tumor-burden range at which control animals began to reach terminal progression, while remaining above baseline and early treatment variability, or when the tumor prevents water and food intake, urination, defecation, or ambulation. These criteria were applied uniformly across all experimental groups and were established prior to study initiation in accordance with institutional animal ethics guidelines. The study end point was defined as the time point at which all control animals had reached humane sacrifice criteria.

### Library Construction, Quality Control, and Sequencing

Messenger RNA was purified from total RNA using poly-T oligo-attached magnetic beads. After fragmentation, first-strand cDNA was synthesized using random hexamer primers, followed by second-strand cDNA synthesis using either dUTP for a directional library or dTTP for a nondirectional library.^66^ The nondirectional library was prepared after end repair, A-tailing, adapter ligation, size selection, amplification, and purification. The directional library was prepared after end repair, A-tailing, adapter ligation, size selection, USER enzyme digestion, amplification, and purification. The library was checked with Qubit and real-time PCR for quantification and a bioanalyzer for size distribution detection. Quantified libraries were pooled and sequenced on Illumina platforms according to the effective library concentration and data amount.

### Data Quality Control

Raw data (raw reads) in fastq format were first processed using in-house Perl scripts. In this step, clean data (clean reads) were obtained by removing reads containing adapters, poly-N, and low-quality reads from raw data. At the same time, the Q20, Q30, and GC contents of the clean data were calculated. All downstream analyses were based on high-quality, clean data.

### Reademapping the Reference Genome

The reference genome and gene model annotation files were downloaded directly from the genome Web site. The index of the reference genome was built using Hisat2 v2.0.5 and paired-end clean 1 reads were aligned to the reference genome using Hisat2 v2.0.5. We selected Hisat2 ^67^ as the mapping tool because Hisat2 can generate a database of splice junctions based on the gene model annotation file, thus providing better mapping results than other nonsplice mapping tools.

### Quantification of Gene Expression

FeatureCounts^68^ v1.5.0-p3 was used to count the number of reads mapped to each gene. Then, the FPKM of each gene was calculated based on the length of the gene and the read count mapped to this gene. FPKM, the expected number of Fragments Per Kilobase of transcript sequence per million base pairs sequenced, considers the effect of sequencing depth and gene length on the read count at the same time and is currently the most used method for estimating gene expression levels.

### Differential Expression Analysis

For DESeq2 ^69^ with biological replicates, differential expression^70^ analysis of three biological replicates per condition was performed using the DESeq2Rpackage (1.20.0). DESeq2 provides statistical routines for determining differential expression in digital gene expression data using a model based on negative binomial distribution. The resulting P-values were adjusted using Benjamini and Hochberg’s approach to control the false discovery rate. Genes with an adjusted P-value of ≤ 0.05, found by DESeq2, were assigned as differentially expressed. (For edgeR^71^ without biological replicates) Prior to differential gene expression analysis, for each sequenced library, the read counts were adjusted by edgeR program package through one scaling normalized factor. Differential expression analysis of two conditions was performed using the edgeR R package (3.22.5). P values were adjusted using the Benjamini and Hochberg method. A corrected P-value of 0.05 and absolute fold change of 2 were set as the threshold for significantly differential expression.

### Enrichment Analysis of Differentially Expressed Genes

Gene Ontology^72^ (GO) enrichment analysis of differentially expressed genes was performed using the clusterProfiler R package, in which the gene length bias was corrected. GO terms with corrected P values less than 0.05 were considered significantly enriched by differentially expressed genes. KEGG is a database resource for understanding high-level functions and utilities of biological systems, such as cells, organisms, and ecosystems, from molecular-level information, especially large-scale molecular data sets generated by genome sequencing and other high-throughput experimental technologies (http://www.genome.jp/kegg/). We used the clusterProfiler R package to test the statistical enrichment of differential expression genes in the KEGG^73^ pathways. The Reactome database brings together various reactions and biological pathways of human model species. Reactome pathways with corrected P values less than 0.05 were considered significantly enriched by differentially expressed genes. The disease ontology (DO) database describes the functions of human genes and diseases. DO pathways with corrected P values less than 0.05 were considered significantly enriched by differentially expressed genes. The DisGeNET database integrates the human disease-related genes. DisGeNET pathways with corrected P values less than 0.05 were considered significantly enriched by differentially expressed genes. ClusterProfiler software was used to test the statistical enrichment of differentially expressed genes in the Reactome, DO, and DisGeNET pathways.

### Gene Set Enrichment Analysis

Gene Set Enrichment Analysis (GSEA) is a computational approach to determine if a predefined Gene Set can show a significant consistent difference between two biological states. The genes were ranked according to the degree of differential expression in the two samples, and the predefined Gene Set was tested to determine if they were enriched at the top or bottom of the list. Gene set enrichment analysis can include subtle changes in the expression levels. We use the local version of the GSEA analysis tool http://www.broadinstitute.org/gsea/index.jsp. GO, KEGG, Reactome, DO, and DisGeNET data sets were used for GSEA independently.

### RNA-seq and Statistical Analysis

RNA-seq was performed with n = 3 biological replicates per group. Differential expression was computed in DESeq2, and genes were considered significant at |log2FC| ≥ 1 with false-discovery rate (FDR, Benjamini-Hochberg) < 0.05. For ordination and clustering, raw counts were transformed using the variance-stabilizing transformation (VST) in DESeq2 and then centered and scaled across genes. Principal component analysis (PCA) was calculated on the top 500 most variable genes (unless stated otherwise). Heatmaps display row Z-scores of VST counts; sample–sample distance was defined as 1 - Pearson correlation, and hierarchical clustering used Ward.D2 linkage. Gene set enrichment analysis (GSEA) was performed on ranked gene lists, reporting normalized enrichment scores (NES) and FDR; analyses used 1000 permutations and standard gene-set databases (e.g., MSigDB Hallmark and KEGG).

### Statistics

Longitudinal comparisons were prespecified at defined imaging days; no posthoc replotting or retesting on relative-growth curves was performed.

## Results and Discussion

### Self-Immolative Tocopherol-Tailored Prodrug Micelles

A composite micelle system from common tocopherol-based constituents was developed to allow either the appendage of hydrophilic GEM at the surface and/or lipophilic compounds in its core (Figure 1a, b and Figures S1–S4). The structural design of the site-selective stable linkage of GEM on the NP surface endows efficacy and predictable behavior, protects the drug from cytidine deaminase (CDA) metabolism, the main degradation pathway for GEM, and allows GSH-mediated GEM release via a self-immolative chemical mechanism. D-α-tocopheryl polyethylene glycol (PEG) 1000 succinate (TPGS) is an FDA-approved pharmaceutical adjuvant with MDR reversal features, while α-Tocopheryl succinate has anticancer properties.^30,31^ TOS was generated in quantitative yield from α-tocopherol and functionalized with a short disulfide linker (TOS-SSOH, Figures S5–S9, S17). The subsequent formation of activated *para*-nitrophenol carbonate allowed the installation and purification of GEM to afford homogeneous and pure GEM carbamate (TOS-SS-GEM, Figures S10–S12). Product purity and *N-*selectivity were confirmed by LC-HRMS, NMR, and UV–vis spectroscopy (bathochromic shift of TOS-SS-GEM compared to GEM) (Figure 1c). Precise *N*-chemoselective engineering is key since *O*-linked products undergo quick hydrolysis to liberate GEM while leaving the cytidine moiety from CDA metabolism.^32,33^

Formulations of micelle@GEM and micelle@PTX were optimized by varying the TPGS/TOS-SS-GEM ratios under self-assembly conditions (in PBS, 25 °C; See Supporting Information) and tolerated high levels of surface modification and/or cargo encapsulation. Spherical micelles 120 ± 20 nm in size with low polydispersity were obtained at an optimal ratio of 4:6 TPGS/TOS-SS-GEM (w/w) (Figure 1d-e and Figures S13–S14). The encapsulation of hydrophobic payloads can be achieved by simply adding a payload in the organic phase during the self-assembly process. Similarly, micelle@PTX was formulated with a similar 4:6 (w/w) TPGS/TOS-SS–OH ratio and a 1:30 (w/w) PTX/NP ratio (See Supporting Information). Only GEM is stimulus-responsive (redox); PTX release is diffusion-controlled under physiological pH. This separation exploits each drug’s physicochemical compatibility with its loading environment and avoids destabilizing chemistries for PTX.

The use of chemically heterogeneous nanoparticle components contributes to intra- and interbatch variability, end-stage formulation issues (e.g., stability, aggregation, and solubility), unpredictable stimuli-responsiveness, and poor PD/PK.^34,35^ Therefore, precise chemical engineering and homogeneous and well-defined chemical systems are crucial for addressing these key problems. Micelle@GEM systems employing *N-*linked TOS-SS-GEM protect the payload from metabolic degradation and are highly stable, while showing a near-quantitative stimulus-release of GEM in GSH-rich environments. To demonstrate this, an LC-HRMS study of TOS-SS-GEM and micelle@GEM over time was carried out in different environments with different GSH concentrations (0–10 mM, Figure 1f). Less than 20% degradation was observed after 120h in PBS pH 7.4, at 37 °C. In contrast, near-quantitative GSH-dependent release of GEM (approximately 99%) was observed for high GSH levels often observed in cancer cells after 72h (PBS pH 7.4, 37 °C, 10 mM GSH)^36–38^ Importantly, minimal GEM release was detected for GSH concentration within healthy cell values,^36,38^ suggesting a potential for a tumor-specific GEM release leading to a more manageable safety profile due to limited systemic and off-target GEM cleavage (Figure 1f). Additionally, incubation of TOS-SS-GEM with CDA afforded negligible metabolite formation compared to free GEM (<5% and ∼ 99% after 90 min, respectively) (Figure 1g and Figure S16). This demonstrates that GEM is protected from plasma CDA metabolism when linked to the micelle, enabling the delivery of high concentrations of unmetabolized GEM to tumor cells, while minimizing systemic exposure, thereby maximizing therapeutic efficacy. The release of PTX from micelle@PTX was evaluated using the dialysis diffusion method and quantified by LC-HRMS, showing high stability (<20% release after 120h, pH 7.4, 37 °C, Figure 1h, Figure S15). As expected, PTX is physically encapsulated in the hydrophobic core and its release is diffusion-controlled (Figure 1h), whereas GEM is covalently linked through a redox-cleavable self-immolative linker and is therefore governed primarily by stimulus-responsive cleavage rather than simple diffusion (Figure 1f).

### *In Vitro* Mechanisms of Enhanced Anticancer Efficacy

NP-based micelles for the delivery of chemotherapeutic agents in the treatment of pancreatic cancer were evaluated *in vitro* using BxPC-3-Luc cells (i.e., pancreatic cancer cell line derived from human pancreatic adenocarcinoma expressing luciferase) in terms of cytotoxicity, cellular uptake, and cell cycle modulation. BxPC-3 cells were extensively used as an in vitro model to mimic PDAC and were used as a formulation-screening model. Thus, in vitro findings reflect formulation-dependent cytotoxicity rather than KRAS-specific signaling effects.^39^ The metabolic activity of cancer cells was measured after treatment with micelle@GEM or micelle@PTX, and then compared to their respective free drugs, Figure 2a-c and Figure S20 and Table S3. Micelle@GEM and micelle@PTX significantly reduced cell metabolic activity compared to the free drugs, enabling 50-fold and 100-fold lower encapsulated drug concentrations to achieve comparable or greater reductions to free GEM and PTX, respectively. The 50–100-fold increase in activity reported here reflects an equi-effect benchmark derived from IC_50_ ratio normalization and should be interpreted as a measure of relative potency shift, not as a pharmacokinetically normalized dose–response equivalence. Micelle formulation increased the apparent in vitro potency of the drugs in BxPC-3-Luc cells, as shown by lower 72 h IC_50_ values compared with the corresponding free drugs, with a 2.3-fold reduction for GEM and a 61.7-fold reduction for PTX. Similarly, the inhibitory concentration for the PTX/GEM combination demonstrated that drug delivery via the nanoparticles enhances metabolic activity, likely due to improved cellular uptake and sustained intracellular drug release. (Figure 2c and Figure S20c).

**Figure 2:**
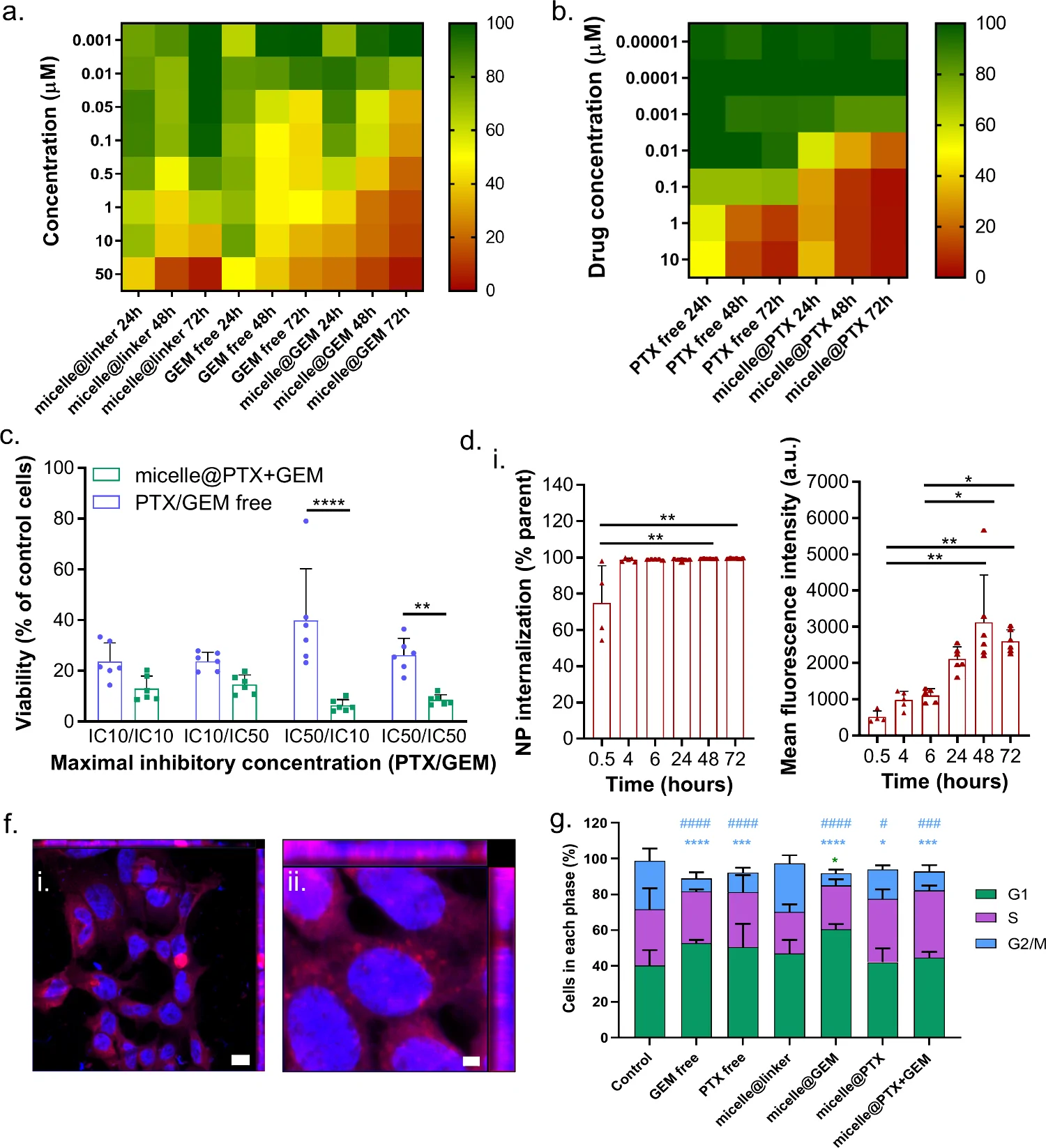
*In vitro* analysis of the prodrug micelles in BxPC-3-Luc pancreatic cancer cells. (a) Heatmap displaying the enhanced metabolic activity of micelle-encapsulated GEM (micelle@GEM) compared with free drugs in pancreatic cancer cells, over a period of 72 h, demonstrating controlled release at therapeutic levels, Figure S20a. (b) Heatmap displaying the enhanced metabolic activity of micelle-encapsulated PTX (micelle@PTX) compared with free drugs in pancreatic cancer cells over a period of 72 h, demonstrating controlled release at therapeutic levels (Figure S20b). (c) Bar graph illustrating the enhanced metabolic activity of micelle-carried PTX and GEM (micelle@PTX+GEM) compared to free drugs on pancreatic cancer cells, with significant reductions in cell metabolic activity at various drug concentrations at 72 h after NPs incubation (Figure S20c). IC50 (μM): GEM = 0.035, micelle@GEM = 0.015, PTX = 0.185, and micelle@PTX = 0.003. IC10 (μM): GEM = 0.01, micelle@GEM = 0.0035, PTX = 0.04, and micelle@PTX = 0.0007, Table S3. Doses reflect a fixed GEM:PTX ratio derived from IC_50_(IC_10_) in vitro. *N* = 3 independent biological experiments, mean ± SD, **p < 0.01, ****p < 0.0001, Kruskal–Wallis test with Dunn’s multiple comparison test. (d) (i) Bar chart showing the percentage of NP internalization over time, indicating rapid uptake by pancreatic cancer cells sustained over 72 h, *N* = 3 independent biological experiments, mean ± SD, *p < 0.05, **p < 0.01, Kruskal–Wallis test with Dunn’s multiple comparison test. (ii) Bar chart of the mean fluorescence intensity quantifying the retention of rhodamine-labeled NPs (NP-Rho) inside the cells, corroborating prolonged intracellular presence. (e) Fluorescence microscopy images (i, ii). Uptake of rhodamine-labeled NPs by pancreatic cancer cells (rhodamine-labeled NPs: red, nuclei: (blue), confirming the internalization observed in quantitative assays. Scale bar: 10 μm (e, i) and 2 μm (e, ii). (f) Bar graph summarizing the cell cycle distribution of pancreatic cancer cells after treatment with free GEM, linker-modified micelles (micelle@linker), and micelles loaded with GEM (micelle@GEM), indicating a possible G1 phase cell cycle arrest by micelle@GEM. *N* = 3 independent biological experiments, mean ± SD, *p < 0.05, vs control, ***p < 0.001 vs control, ***p < 0.0001 vs control; #*p* < 0.05 vs micelle@linker, ###*p* < 0.001 vs micelle@linker, ####*p* < 0.0001 vs micelle@linker, ordinary ANOVA test with Tukey’s multiple comparisons test.

Concerning cellular uptake and retention, we observed internalization and retention of rhodamine-labeled NPs (NP-Rho) in pancreatic cancer cells (Figure 2d, e). The data indicate that NPs are not only rapidly internalized but also retained within the cells over a 72h period. This sustained retention is essential for continuous drug action inside cancer cells, and may contribute to the increased cytotoxicity observed in Figure 2c. Confocal microscopy images (Figure 2e) visually confirmed the cellular uptake of micelles loaded with rhodamine, with a clear distinction between cells with internalized NP-Rho, highlighting the ability of NPs to penetrate and reside within cancer cells, which correlates with the quantitative data in Figure 2d. To understand whether the effects of the drugs on the cell cycle were altered when micelles were used as a delivery vehicle, cells were incubated with IC_50_ doses of each system. The samples were analyzed by flow cytometry after 72h. GEM is known to cause S phase arrest,^40^ whereas PTX arrests the cell cycle during the G2/M phase in various cancer cells, including pancreatic cells.^41,42^ In our results, micelle@GEM-treated cells showed an increase in G1 phase indicative of G1 arrest was more pronounced (approximately 20% more cells in G1) compared to the control free GEM (Figure 2f and Figure S21). Cells treated with micelle@PTX showed an increase in phase S and a decrease in G2/M phase, where they remained arrested. This effect was slightly higher in the micelles carrying PTX and GEM (micelle@PTX+GEM) group (Figure 2f). These results corroborate our previous findings that micelles potentiate the effects of these drugs (Figure 2c).

Collectively, these assays and mechanisms indicate that micelle@GEM and micelle@PTX are superior alternatives to free drugs, alone or in combination, underpinning their enhanced antitumor activity. GEM or PTX micelles have emerged as potent modalities for improving the therapeutic index of chemotherapeutic agents in vivo by enhancing drug delivery and retention, exerting potent cytotoxic effects, potentially inhibiting cell migration, and inducing cell cycle arrest.

### Mapping Prodrug Micelle Biodistribution

To map and evaluate the biodistribution of the prodrug micelles, temporal and spatial distribution profiles were analyzed. The previous optimized micelles were embedded in 4 wt % hyaluronic acid (HA)-based hydrogel (hyd) that can work as a minimally invasive local drug delivery system due to its shear thinning properties (i.e., the viscosity decreases when shear rate increases**;** Figures S22–S23). This allows the system to further maximize therapeutic efficacy and decrease systemic toxicity.^43^ Scanning electron microscopy of HA-based hydrogels revealed a porous structure in empty hydrogels and changes in surface topology and porosity, with a homogeneous distribution of micelles containing PTX, GEM, or both within the hydrogel matrix (Figure S24). Moreover, we have performed LC-HRMS analysis of drug release formulations embedded in the HA-based hydrogel using two different methods: the dialysis diffusion method and transwell cell culture inserts and nontraceable amounts of PTX or GEM were detected, which is consistent with the sustained release profile of the micelles within the gel. Micelles were characterized after hydrogel release and were stable, maintaining similar hydrodynamic size and charge, Figure S19. Rhodamine-labeled micelles embedded in HA-based hydrogel were administered intraperitoneally (500 μL/mouse). Whole-body fluorescence imaging over 24 h revealed an intense signal immediately postinjection, absent in “no inject” controls (Figure 3a). Signal intensity peaked at 0.5 h, gradually declined, and localized predominantly in the peritoneal cavity by 24 h (Figure 3a, b). This indicates hydrogel-mediated micelle retention, facilitating prolonged residence in the abdominal cavity and supporting prolonged compartmental exposure of peritoneal tissues relevant to the carcinomatosis model and clearance organs. Individual organ assessments showed greater accumulation of rhodamine-labeled micelles in the liver and pancreas (Figure 3c). This 24 h organ ranking qualitatively tracks the *in vivo* peritoneal ROI distribution observed at matched time points. This was expected because of their roles in filtration and clearance in the reticuloendothelial system. In addition, hydrogel encapsulation significantly enhanced the retention of micelles at the injection site and prolonged their localized presence in the peritoneal cavity compared to direct injection. HA-based hydrogels represent a promising strategy for the delivery of therapeutic agents, allowing prolonged contact with abdominal organs and reducing off-target effects, as can be observed by increased signal in the abdominal area at 9h (Figure 3a, b). The peak accumulation at early time points suggests a rapid initial distribution, which could provide an early exposure window in the peritoneal compartment prior to therapeutic assessment in tumor-bearing mice and minimize exposure to nontarget tissues.

**Figure 3:**
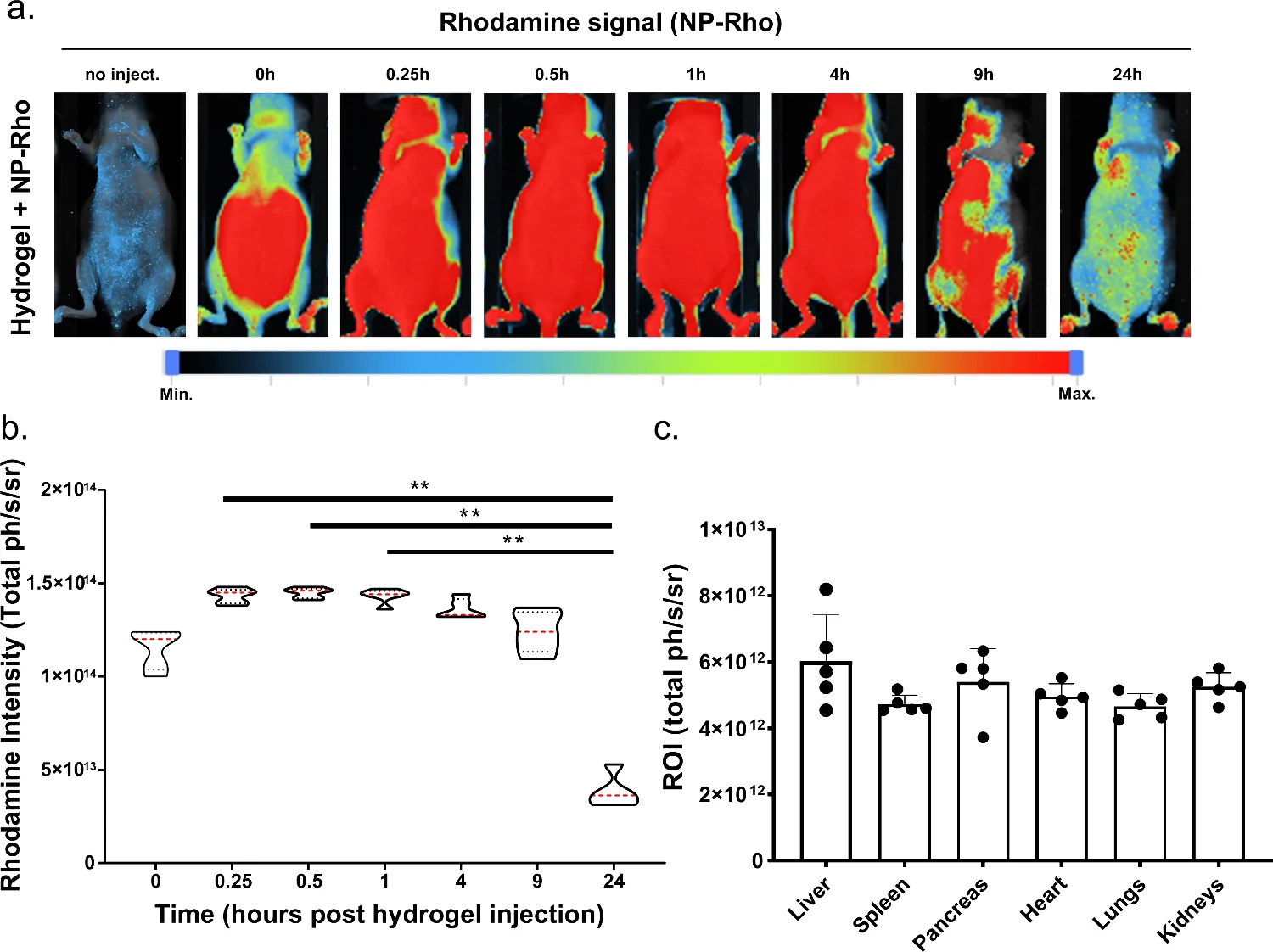
Evaluation of biodistribution of nanoparticle-based micelle delivery systems in live animals. (a) Sequential fluorescence imaging highlighting the peritoneal distribution immediately after intraperitoneal administration and subsequent abdominal localization. The images compare the signal intensity from no injection to 24 h post-injection, highlighting the systemic circulation and localization of the micelles. (b) Violin plot quantifying the fluorescence intensity of the rhodamine signal from the micelles at various time points after hydrogel injection, providing a statistical distribution of the biodistribution data over time. *N* = 5, mean ± SD, **p < 0.01, ordinary ANOVA test with Kruskal–Wallis test multiple comparisons test. (c) Bar graph depicting the accumulation of rhodamine-labeled micelles in major organs at the 24-h time point. This graph shows the relative uptake of micelles by organs such as the liver, spleen, pancreas, heart, lungs, and kidneys, which is essential for evaluating the specificity and potential off-target effects of the nanocarrier system, which affect therapeutic outcomes, depending on the balance between achieving effective drug concentrations at the tumor and metastatic sites (in this case, pancreas, liver, and spleen). *N* = 5, mean ± SD.

### Nanobooster Prodrug Micelles for *In Vivo* Pancreatic Cancer

The therapeutic efficacy of drug-loaded micelles embedded in HA hydrogel was assessed by PDAC in vivo model. The combination of the two drugs denotes coembedding of single-drug micelles within the depot. First, BxPC3-Luc were injected intraperitoneally (Figure 4a) and at day 21 post-tumor induction (Day 0, Figure 4b), the proposed in vivo model resembles PDAC late-stage at diagnosis, recapitulating rapid progression, metastasis, and tumor aggressiveness (Figure 4b).^44–46^

**Figure 4:**
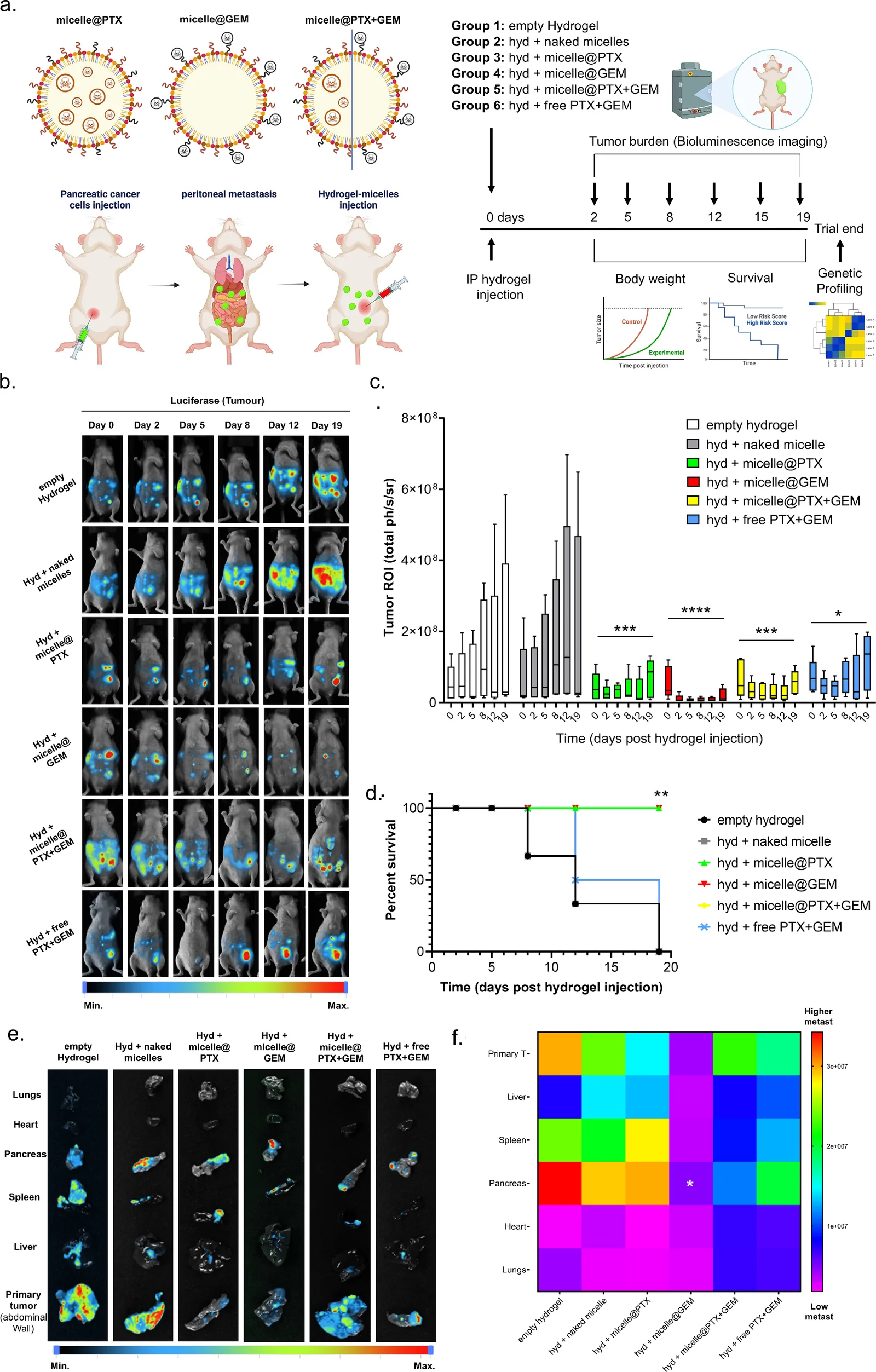
Evaluation of efficacy of nanoparticle-based micelle delivery embedded within hydrogel matrix in a preclinical model of pancreatic cancer. (a) Schematic representation of the NP formulations micelle@PTX, micelle@GEM, and micelle@PTX+GEM, depicting their structural composition. Below is an illustrative timeline of the experimental workflow, including the injection of 2.4 × 10^6^/mouse BxPC3-Luc pancreatic cancer cells intraperitoneally. At day 21 post tumor induction, mice were distributed in 6 groups (*n* = 5): (1) Hydrogel, (2) Hydrogel with empty micelles, (3) Hydrogel with free PTX+GEM, (4) Hydrogel with micelles@PTX, (5) Hydrogel with micelles@GEM, (6) Hydrogel with micelles@PTX+GEM; the formulations were administered intraperitoneally. The subsequent monitoring stages included bioluminescence measurements on days 0, 2, 5, 8, 12, and 19 post-treatments and body weight. The end of the trial (day 19) finished with the collection of several tumoral foci identified in the organs and abdominal wall for genetic profiling. (b) Longitudinal bioluminescence imaging of mice bearing luciferase-tagged pancreatic tumors treated with different hydrogel formulations for 19 days. The images show the progression and response of the tumors to each treatment over time. (c) Quantitative analysis of tumor growth as measured by the bioluminescent signal intensity from the tumors in mice. The bar chart shows signal quantification at various time points post-treatment, with significant growth inhibition observed in the hyd + micelle@PTX+GEM group. Statistical analysis was performed using a one-way analysis of variance (Brown-Forsythe) test, followed by Holm-Šídák’s posthoc multiple comparisons test for pairwise group differences. ****P < 0.0001, ***P < 0.001, *P < 0.05). Group means at days 0, 2, 5, 8, 12, 19; variance homogeneity was assessed using Bartlett’s test, confirming significant heteroscedasticity (*p* = 0.0036); bars show mean ± SD. (d) Kaplan–Meier survival curve illustrating the survival probability of mice following treatment with different hydrogel formulations. *N* = 5 animals, **p < 0.005 empty hydrogel vs micelle@PTX, micelle@GEM, and micelle@PTX+GEM, **p < 0.005 hyd + naked micelles vs micelle@PTX, micelle@GEM, and micelle@PTX+GEM, **p < 0.005 hydrogel + free PTX+GEM vs micelle@PTX, micelle@GEM, and micelle@PTX+GEM, log-rank (Mantel-Cox) test. Table S5 reports animals at risk at each time interval and median survival. (e) *Ex vivo* bioluminescence imaging of organs extracted from treated mice was performed to assess metastatic spread. The images show the presence or absence of metastases in the lungs, heart, pancreas, spleen, liver, and the primary tumor location in the abdominal wall. (f) Heatmap representing the metastatic burden across different organs in each treatment group. The color gradient indicates the relative intensity of the metastases, with cooler colors denoting lower levels of metastatic cells. *N* = 5, mean ± SD, * *p* < 0.05, vs control, two-way ANOVA test with Tukey’s multiple comparisons test.

A single injection of hyd + micelle@GEM induced highly significant tumor regression (Figure 4b, c), with duration of response for more than 10 days across all tumor loci as observed by a sustained negligible luminescence signal. Similarly, hyd + micelle@PTX and hyd + micelle@PTX+GEM inhibited tumor growth (Figure 4b, c), corroborating the *in vitro* results, and indicating a potent *in vivo* antitumor effect. At the tested fixed-ratio and total payload, the GEM exposure in micelle@GEM exceeded that of the combination arm by design, which, together with faster GEM release relative to slower PTX diffusion, accounts for the stronger inhibition observed with micelle@GEM. These differences reflect intended dosing and kinetics rather than antagonism. A single injection of hyd + drug-carrying micelles resulted in prolonged survival compared with the control groups (Figure 4d), supporting the higher effective delivery of therapeutic agents. One hundred percent of the animals treated with hyd + drug-carrying micelles were alive during the entire experiment with a single dose of treatment; however, no animals have survived in the other groups for the exact same period (Figure 4d). At the final end point, the tissues and organs were assessed for the presence of pancreatic cancer metastasis. In addition to the abdominal wall, the abdominal organs, such as the liver, pancreas, and spleen, had the highest number of metastases (Figure 4e, f). As previously observed via live imaging, animals treated with hyd + micelle@GEM exhibited markedly less luminescence, indicating reduction of the metastatic implants, which was closely followed by animals that received a single injection of hyd + micelle@PTX+GEM (Figure 4e, f). The heatmap that quantifies the metastatic burden in different organs corroborates the findings from bioluminescence imaging, with the lowest metastatic burden observed in the combination treatment group (Figure 4f). Throughout the experiment, no significant body weight loss was observed (Figure S25), suggesting the absence of acute systemic toxicity at the doses and time points evaluated. Accordingly, conclusions regarding systemic or local biocompatibility are preliminary and confined to the absence of acute weight-based toxicity. The study was not designed for long-term comparative toxicology versus free drugs; such chronic safety evaluation will be addressed in a dedicated follow-up. Long-term toxicology will require repeat-dose studies, hematology, serum biochemistry (hepatic and renal function markers), histopathological examination of key organs (liver, kidney, spleen, lung), local peritoneal reaction assessment, and delayed organ analysis.

The data from these experiments provide strong evidence for the potential of micelle-carried chemotherapy in treating pancreatic cancer. The use of micelles as a delivery system embedded within the hydrogel seems to enhance the efficacy of PTX and GEM, likely due to improved drug solubility, and sustained release of drugs in the tumor microenvironment. This approach may help to overcome the challenges of poor drug stability associated with conventional chemotherapy. The reduced tumor burden decreased metastatic spread and extended survival times in the GEM alone or in the combination treatment group suggest a promising therapeutic strategy. When delivered in a targeted fashion, the synergy between PTX and GEM has the potential to maximize the therapeutic outcomes. The therapeutic conclusions drawn here are specific to advanced, metastatic PDAC with peritoneal dissemination and should not be extrapolated to earlier-stage, localized, orthotopic, or postsurgical disease settings, which will require additional validation in dedicated in vivo models. Indeed, cross-model validation in KRAS mutant cell lines, orthotopic models, organoids, and patient-derived systems will be necessary before broader generalization.

### Tumor and Metastasis Genetic Profile in Response to Prodrug Micelles

To explore the genetic profiling of peritoneal tumor implants versus metastatic tumor deposits under different treatment conditions using the tested prodrug micelles, we extracted RNA from the different peritoneal tumor implants and their metastatic tumor deposits in the spleen and liver of the peritoneal pancreatic carcinomatosis mouse model. Whole-body imaging (Figure 5a) confirmed the presence of metastatic sites and peritoneal tumor implants, which is crucial for understanding the spread of this type of cancer. The differential accumulation of micelles in peritoneal tumor implants versus metastatic tumor deposits raises the possibility of tailoring nanoparticle properties to enhance delivery to specific tumor niches, a critical consideration given the notorious challenges of drug delivery in PDAC due to its dense stromal barrier and immunosuppressive microenvironment.^17^ We performed a genetic analysis of peritoneal tumor implants, specifically examining the impact of various micelle-based delivery systems on gene expression profiles (Figures S26–S32). This analysis falls at the intersection of oncogenomics, cancer therapeutics, and nanotechnology, offering insights into the precise targeting of oncogenic pathways through advanced drug delivery mechanisms. The stromal penetration capacity of this delivery system in primary desmoplastic PDAC tumors remains to be established.

**Figure 5:**
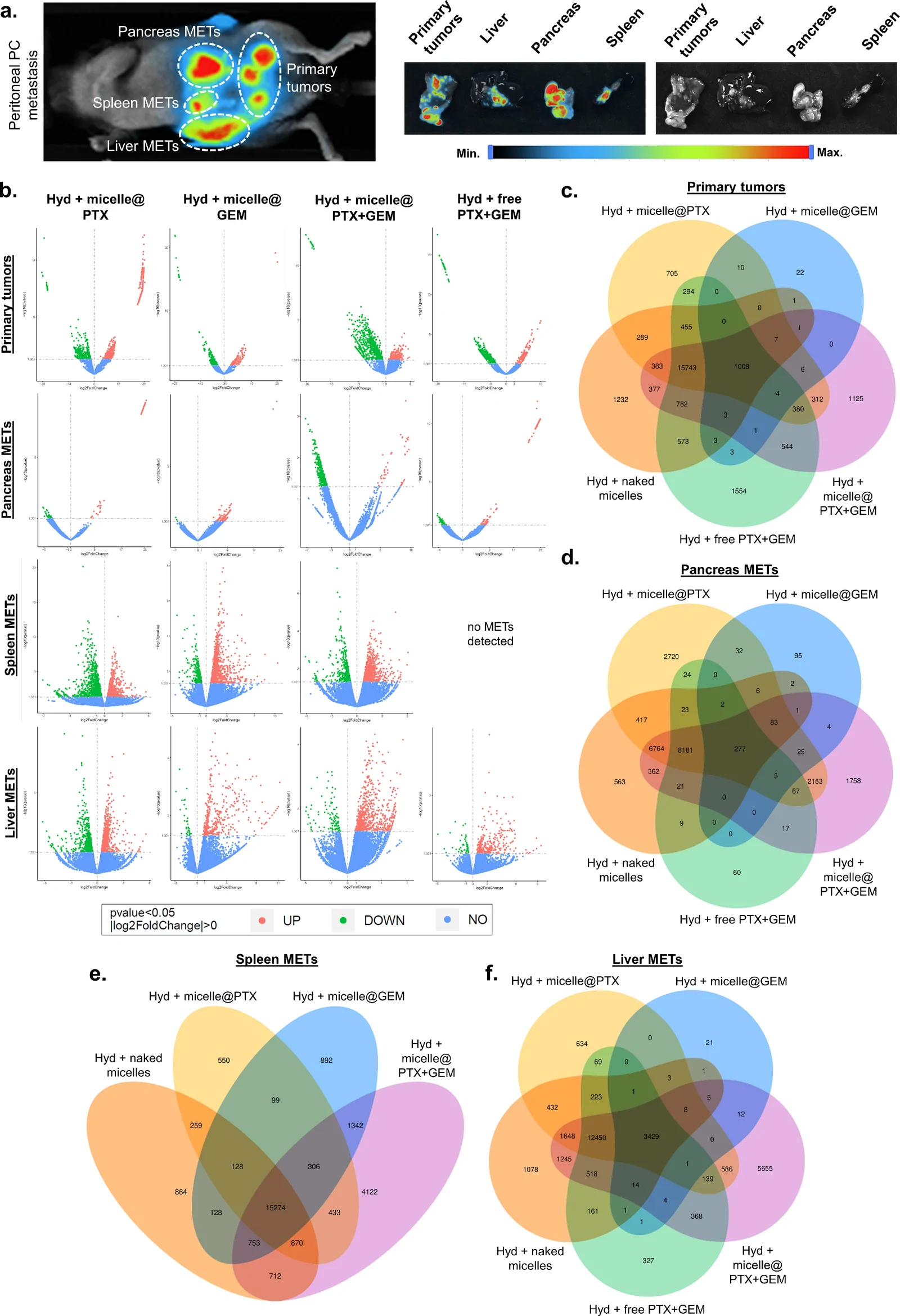
Genetic responses of peritoneal tumor implants versus metastatic tumor deposits to different prodrug micelles and their impact on gene expression profiles. (a) Multimodal imaging showing the localization of peritoneal tumor implants versus metastatic tumor deposits. The left image employs live bioluminescence imaging scanning to highlight areas of high metabolic activity indicative of tumor sites, whereas the right image displays the respective organ-specific distribution of metastases. (b) Volcano plots representing differential gene expression in peritoneal tumor implants versus metastatic tumor deposits treated with different formulations: hyd + micelle@PTX, hyd + micelle@GEM, hyd + micelle@PTX+GEM, and hyd + free PTX+GEM. Each plot contrasts the log2 fold change against statistical significance (p-value), with upregulated genes in red, downregulated genes in green, and nonsignificant changes in blue. Spleen METs in the free PTX+GEM group were not present in any animal. (c) Venn diagram depicting the overlap of differentially expressed genes in peritoneal tumor implants treated with hyd + micelle@PTX, hyd + micelle@GEM, hyd + naked micelles, and hyd + free PTX+GEM, highlighting the shared and unique gene expression changes induced by each treatment. (d) Venn diagram illustrating shared and unique differentially expressed genes in pancreatic metastases (Pancreas METs) treated with the same four formulations as in panel. (e) Venn diagram showing the overlap of differentially expressed genes in spleen metastases (Spleen METs) across the four treatment groups. (f) Venn diagram representing the differentially expressed genes in liver metastases (Liver METs) for each treatment condition, demonstrating the distinct genetic response of liver metastatic sites to micelle-based therapies. *n* = 3 per group; DE cut-offs |log2FC| ≥ 1, FDR < 0.05; heatmap values are row Z-scores; distance/linkage as in Experimental Section.

To provide a clear visualization of the transcriptomic changes induced by treatments, volcano plots were drawn (Figure 5b), showing that the magnitude and significance of gene expression shifts underscored the pharmacogenomic impact of micelle-carried PTX and GEM. Notably, these plots underscore the differential pharmacogenomic responses of peritoneal tumor implants versus metastatic tumor deposits, indicating spatial heterogeneity in therapeutic susceptibility. The differential gene expression between peritoneal tumor implants versus metastatic tumor deposits suggests that micelle-mediated delivery may influence tumor behavior in a site-dependent manner, offering potential for targeted therapeutic interventions that address the heterogeneity within the tumor microenvironment.^47^

To reveal the complexity of treatment responses, Venn diagrams (Figure 5c-f) show substantial overlap in altered gene expression, suggesting common pathways affected by micelle-encapsulated drugs. Concerning peritoneal tumor implants, the largest unique set of genes affected was the combination of free PTX+GEM, followed by hyd + micelle@PTX. Only 3 genes were affected across all treatments, suggesting that each treatment mostly affected distinct sets of genes (Figure 5c). Regarding pancreatic METs, a significant number of genes were uniquely affected by the hyd + micelle@PTX treatment. The overlaps were small compared to unique gene alterations, indicating that different mechanisms might be involved in each treatment in pancreatic METs (Figure 5d). Regarding spleen METs, the hyd + micelle@PTX+GEM combination affected the largest unique number of genes. The central overlap, in which all treatments affected gene expression, included 306 genes, indicating common targets or pathways (Figure 5e). In liver METs, hyd + micelle@GEM showed the largest unique set of altered genes, which suggests its potent effect in the liver METs context. Interestingly, no genes were commonly altered by all treatments, highlighting the specificity of each treatment in liver METs (Figure 5f). This comparative genomic approach is instrumental in determining the unique and shared pathways modulated by individual and combined treatments. The intersectional subsets could suggest convergent mechanisms of action or compensatory pathways activated in response to the treatment. The unique gene expression changes induced by the combination of PTX and GEM may reflect a synergistic mechanism, potentially providing a broader blockade of oncogenic signaling pathways than either agent alone. Concerning peritoneal tumor implants (Figure 5c), the central intersection elucidates a core set of genes that are commonly influenced across all treatment modalities, potentially implicating fundamental processes in tumor survival and resistance. For pancreatic METs (Figure 5d), the significant overlap between PTX and GEM treatments suggests a shared therapeutic impact on metastatic tumor deposits, whereas unique gene sets might reflect drug-specific actions. Both spleen METs (Figure 5e) and liver METs (Figure 5f) highlighted the site-specific genomic response to treatment, which is critical given the organ-specific microenvironmental influences on metastatic cancer cell behavior.

These findings prompt reconsideration of combination chemotherapies, where the role of drug formulation and delivery method becomes as critical as the agents chosen. The overall implications of this analysis highlight the transformative potential of nanoparticle technology in PDAC treatment. The ability of micelles embedded within hydrogel matrix to alter gene expression profiles suggests that the efficacy of chemotherapy can be enhanced beyond the broad action of the drugs themselves. These insights into gene expression modulation offer a roadmap for further investigation into specific pathways, with the aim of developing more effective and personalized therapeutic strategies. Our data emphasize the need to consider micelle formulation as an integral component of the therapeutic regimen, with the potential to alter the pharmacokinetics and pharmacodynamics of chemotherapeutic agents, thereby redefining the standard of care for patients with PDAC.

### Modulation of Genomic Pancreatic Cancer Signaling Pathways in Peritoneal Tumor Implants versus Metastatic Tumor Deposits

Detailed transcriptomic analysis of peritoneal tumor implants versus metastatic tumor deposits treated with different drug formulations encapsulated within micelles is essential to understand the molecular dynamics induced by nanocarrier-mediated drug delivery and its implications for targeted cancer therapy. Using gene ontology (GO) analysis (Figure 6a) for differentially expressed genes, categorized by biological processes, cellular components, and molecular functions, allows for the classification of genes into hierarchies based on their associated biological processes and functions, offering a macroscopic view of the cellular activities impacted by the treatments. This analysis is key for identifying overarching biological themes and cellular machineries that are perturbed by treatment, potentially unveiling new targets for therapeutic intervention. The GO heatmap offers a granular view of the modulation of critical signaling pathways in response to treatment, showing how micelle drug delivery reprograms the cancer cell transcriptome. We observed that all prodrug micelles significantly altered GO pathways, mainly within pancreatic METs and peritoneal tumor implants, compared to free drugs. This is clear when observing a higher incidence of upregulating processes for micelle@PTX and hyd + micelle@PTX+GEM in both peritoneal tumor implants and pancreatic METs (Figure 6a). This upregulation suggests that the drug formulations actively influence key cellular mechanisms, potentially leading to increased stress responses, apoptosis, and immune activation within the tumor microenvironment. The capacity of these treatments to modulate gene expression can be attributed to the enhanced cellular uptake and sustained release properties of the micelles, ensuring that the chemotherapeutic agents maintain prolonged contact with their intracellular targets, thereby amplifying their therapeutic effects. In contrast, a higher incidence of down-regulating processes was observed when using hyd + micelle@GEM in pancreatic METs and upregulated in peritoneal tumor implants (Figure 6a). Here, upregulation could indicate suppression of genes associated with metastatic progression, angiogenesis, or other prosurvival pathways. Interestingly, the same treatment upregulates processes in peritoneal tumor implants, which might reflect a context-dependent response, where peritoneal tumor implants cells and metastatic cells exhibit differential sensitivity to GEM when delivered via micelles.

**Figure 6:**
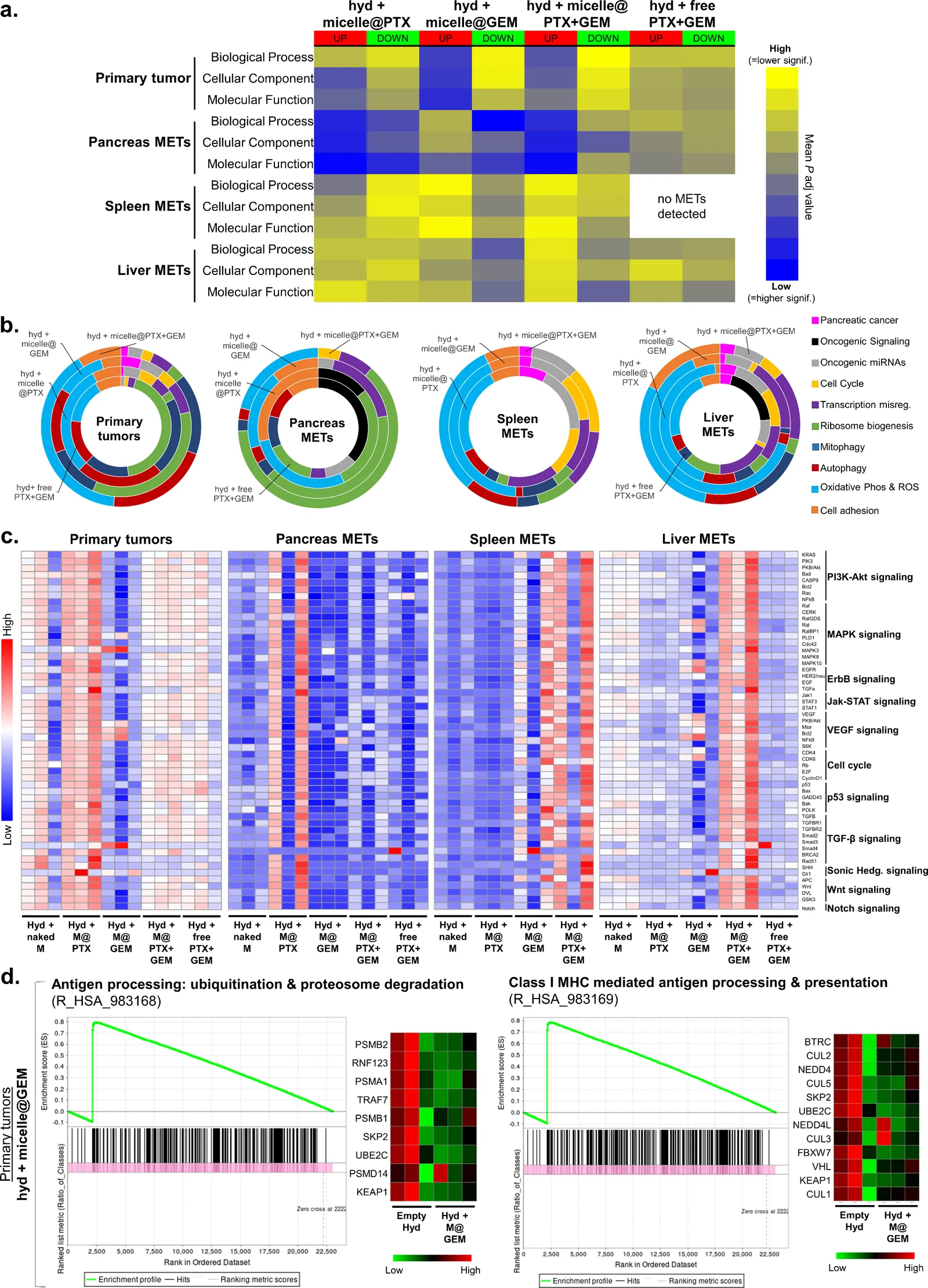
Analysis on the genetic and molecular responses of pancreatic cancer to prodrug micelles. (a) Heatmap summarizing the gene ontology (GO) terms affected by different treatment regimens in peritoneal tumor implants and metastatic tumor deposits. The heatmap depicts the level of gene expression associated with biological processes, cellular components, and molecular functions, with color intensity indicating the degree of up-regulation or down-regulation. Spleen METs in the free PTX+GEM group were not present in any animal. (b) Circular bar plots indicating the pathway enrichment analysis for peritoneal tumor implants andmetastatic tumor deposits (pancreas, spleen, liver) treated with different drug formulations. Each ring represents a specific treatment, and the bars correspond to the level of pathway activity categorized by cancer-associated processes and signaling mechanisms. (c) Heatmap displaying the expression profile of genes involved in key signaling pathways across different treatments in peritoneal tumor implants and metastatic tumor deposits in and pancreatic, splenic, and liver. The color gradient from blue to red indicates the expression levels from low to high, respectively, highlighting the treatment-induced modulation of the signaling pathways critical to cancer progression. (d) Gene Set Enrichment Analysis (GSEA) plot highlighting the enrichment of the ‘Antigen Processing: Ubiquitination & Proteasome Degradation’ and ‘Class I MHC Mediated Antigen Processing & Presentation’ gene sets related to pancreatic cancer, suggesting an involvement of hyd + micelle@GEM group for peritoneal tumor implants in the disease’s molecular mechanisms, as well as in immune response mechanisms in the disease. The analysis revealed differential enrichment of genes involved in antigen processing through ubiquitination and proteasome degradation (left), and class I MHC-mediated antigen processing and presentation (right). Key genes, such as *PSMB2*, *RNF123*, *PSMA1*, *TRAF7*, and *SKP2*, showed notable differences in expression between treatments, highlighting the impact of micelle-mediated delivery on these pathways. The enrichment plots (green lines) indicate the distribution of the ranked metric scores for the genes in each set, with the vertical black lines representing the position of each gene in the ranked list. GSEA shows NES and FDR for highlighted pathways (e.g., PI3K-Akt, MAPK); thresholds and databases as in Experimental Section. GSEA enrichment plots and corresponding heatmaps for peritoneal tumor implants comparing Hyd + M@GEM versus Empty Hyd. Antigen processing: ubiquitination and proteasome degradation showed positive enrichment in Hyd + M@GEM-treated tumors (R_HSA_983168; ES = 0.7979; NES = 1.3211; nominal *p* < 0.001; FDR q = 1.000). Class I MHC-mediated antigen processing and presentation showed a similar enrichment pattern (R_HSA_983169; ES = 0.7821; NES = 1.2996; nominal *p* < 0.001; FDR q = 1.000). Heatmaps display representative pathway-associated genes and show treatment-associated expression differences between Empty Hyd and Hyd + M@GEM tumors. These analyses are interpreted as exploratory pathway-level evidence supporting activation of antigen-processing and MHC-I presentation programmes.

To display the enrichment of the different oncogenic pathways and processes,^48^ including signaling, transcriptional misregulation, ribosome biogenesis, and others across the peritoneal tumor implants and metastatic sites, circular bar plots of KEGG analysis are shown (Figure 6b). Each concentric ring represents a specific treatment condition, and the length of the bar correlates with the significance of enrichment of the connected pathways. This analysis helps visualize the extent to which each pathway is influenced by different treatment conditions, revealing critical insights into how micelle drug delivery can modulate cancer pathophysiology. We observed a broader impact of prodrug micelles on the tumor microenvironment, affecting processes such as cell adhesion, autophagy, and oxidative stress response, which are crucial for tumor growth and metastasis.

To explore the molecular pathways of the pancreatic cancer response to treatment,^49^ gene differential analysis clustering was performed (Figure 6c). Our data suggest differential gene expression profiles in response to treatment with PTX and GEM, either as a single agent or in combination, encapsulated within micelles. These transcriptomic changes were not observed for the same free drugs. This likely reflects weaker and more heterogeneous effective intratumoral exposure in the free-drug condition, whereas the micelle platform generated a stronger and more synchronized pharmacological perturbation that became statistically detectable. This is significant as it could indicate that micelle encapsulation not only enhances drug solubility and delivery efficiency but also may alter the drug’s interaction with cellular targets, thus affecting gene expression uniquely. In our system, this occurs through several converging mechanisms, that is protection of GEM from CDA-mediated degradation, GSH-triggered intracellular GEM release, improved PTX solubilization, prolonged intracellular retention, and the known MDR-reversal and P-gp inhibitory properties of TPGS. Together, these effects are expected to increase the magnitude and duration of intracellular drug exposure, which in turn can produce stronger downstream suppression signatures in canonical survival, proliferation, and angiogenic pathways than the free-drug condition. The heatmap elucidates the expression patterns of genes within pivotal signaling axes, such as PI3K-Akt, MAPK, and VEGF. These pathways are cornerstones of tumor survival, proliferation, and angiogenesis, and their dysregulation is often implicated in pancreatic cancer resistance to conventional therapies. The PI3K-Akt pathway is a key regulator of cell survival and proliferation, often hyperactivated in pancreatic cancer.^50^ Aberrations in this pathway can lead to enhanced growth, survival, and metabolism of cancer cells, and are associated with resistance to chemotherapy. Our analysis revealed significant modulation of this pathway by micelle@PTX and micelle@GEM, suggesting that nanoparticle delivery can potentially enhance the drug’s inhibitory effects on this pathway, which may result in reduced cell metabolic activity and increased apoptosis of pancreatic cancer cells. Additionally, the MAPK pathway plays a critical role in the transduction of extracellular signals in cellular responses, including proliferation, differentiation, and migration. Its involvement in pancreatic cancer has been linked to aggressive tumor behavior and metastasis. The treatment-specific changes in gene expression within the MAPK pathway depicted in the heatmap could be indicative of the effectiveness of the micelle-based formulations in curtailing these processes.^51^ This is especially relevant for metastatic disease, where inhibiting MAPK signaling can significantly impact tumor spread and patient outcomes. This activation is in accordance with previous studies, which showed the upregulation of genes that regulate networks involved in KRAS activation.^52,53^ The VEGF signaling pathway is vital in angiogenesis, which is essential for tumor growth and metastasis by providing the necessary blood supply.^54^ Pancreatic tumors often exhibit high levels of VEGF, promoting a robust angiogenic network. The observed expression patterns (Figure 6c) suggest that micelle encapsulation of PTX and GEM can affect this angiogenic signaling, potentially leading to reduced neovascularization and, consequently, limited tumor nourishment and growth. By affecting these pathways, nanoparticle-based micelles could not only impede peritoneal tumor implants growth but also target the metastatic cascade at various stages, from local invasion to distant colonisation. This is in agreement with the *in vivo* efficacy results (Figure 4). This targeted molecular disruption, combined with the advantages of micelle delivery, such as improved drug stability and nonacute weight loss, marks a significant advancement in the therapeutic strategy against pancreatic cancer by recalibrating critical signaling networks that are often hijacked by pancreatic cancer cells.

Moreover, Gene Set Enrichment Analysis (GSEA) showed significant enrichment of the gene sets the ‘Antigen Processing: Ubiquitination & Proteasome Degradation’ and ‘Class I MHC Mediated Antigen Processing & Presentation’ gene sets related to pancreatic cancer, suggesting an involvement of hyd + micelle@GEM group for peritoneal tumor implants (Figure 6d). This suggests that hyd + micelle@GEM alters processes crucial for protein homeostasis in cells and can be involved in the regulation of various cellular processes, including the cell cycle, apoptosis, and DNA repair mechanisms.^7,55^ Dysregulation of this pathway has been implicated in cancers, including pancreatic cancer, as it may affect the stability and degradation of oncoproteins and tumor suppressors.^56^ Specifically, the enrichment plots and heatmap (Figure 6d, right panel) suggest that the treatment modulates the ubiquitination and proteasome degradation pathways, which are crucial for protein turnover and antigen processing in cancer cells. The genes highlighted, including *PSMB2*, *RNF123*, *PSMA1*, *TRAF7*, and *SKP2*, mechanistically plausible mediators of treatment response because they converge on ubiquitination, proteostasis, stress adaptation, and cell-cycle control. *PSMB2* and *PSMA1* are part of the proteasome complex and are essential for the degradation of ubiquitinated proteins, which can include misfolded or damaged proteins, as well as regulators of the cell cycle and apoptosis. Altered expression of these genes can influence the sensitivity of cancer cells to chemotherapeutic agents such as GEM by affecting protein homeostasis and cell survival pathways. *RNF123*, *TRAF7*, and *SKP2* encode E3 ubiquitin ligases that tag proteins with ubiquitin to mark them for degradation. Changes in their expression levels may reflect alterations in the ubiquitination process, potentially affecting a wide range of cellular processes including cell signaling, DNA repair, and immune response. The enrichment of this gene set in pancreatic cancer suggests that there may be significant changes in the expression or activity of these genes, which could contribute to the development or progression of the disease. Micelles can enhance the delivery of GEM to peritoneal tumor implants, potentially overcoming some drug resistance mechanisms by ensuring that higher concentrations of the drug reach the tumor cells, thereby amplifying its effects on gene expression related to ubiquitination and proteasome-mediated degradation.^57^ This could be particularly important for genes involved in protein degradation pathways, which may contribute to drug resistance. The gene set of class I MHC Mediated antigen processing and presentation involves genes that are crucial for presenting peptide fragments derived from pathogens or cancer cells to T cells, which is an essential part of the immune response to cancer.^58^ From the heatmap (Figure 6d, left panel), it is evident that several key genes involved in this pathway exhibit varying expression levels following treatment, such as *BTRC*, *NEDD4*, *CUL2*, *SKP2*, *UBE2C*, and *NEDD4L*. Alterations in these genes may reflect a shift in the cellular machinery responsible for antigen processing, potentially affecting the visibility of cancer cells to the immune system. *CUL1*, *FBXL3*, and *KEAP1* were also depicted on a heatmap with marked expression differences. *CUL1* is part of the SCF complex (SKP1-CUL1-F-box protein), an E3 ubiquitin ligase complex that regulates protein degradation and is crucial for controlling the levels of various cellular proteins, including those involved in the immune response. *FBXL3* is involved in the regulation of circadian rhythms and cellular oxidative stress responses. *KEAP1*, which regulates the activity of *NRF2* in response to oxidative stress, can influence the presentation of antigens on MHC class I molecules by modulating the intracellular redox state and degradation of *NRF2*.^59^ The enrichment of this gene set suggests that there may be an active immune response to pancreatic cancer cells, or that pancreatic cancer cells may alter this pathway to evade immune detection.^60^ Enhancing antigen presentation could increase the efficacy of immunotherapies in metastatic settings, possibly in combination with micelle-carried GEM, to reduce tumor burden and stimulate immune clearance of cancer cells.

To correlate clinical data from GEO, GTex, TCGA, and TARGET databases (which includes >15 K normal, >40 K tumor, and ∼1 K metastasis patient samples) with our results, an in-depth examination of pancreatic cancer progression and treatment was conducted. These clinical data highlight the significant role of key genes in ubiquitination and proteasome degradation pathways. Oncoprint visualization (Figure S33) revealed the frequent occurrence of genetic alterations in genes such as *RNF123* and *PSMA1*, which are integral to these pathways. This genetic context aligns with the differential gene expression profiles presented in Figure S34, particularly the pronounced upregulation of the ubiquitin-conjugating enzyme *UBE2C* in metastatic tumor samples, indicating its potential as an indicator of disease progression. When correlating these results with previous analyses (Figure 6d), it is evident that the genes highlighted here are not only frequently mutated in pancreatic cancer but also exhibit altered expression levels in tumor and metastatic contexts. To complement these findings, the survival analysis shown in Figure S35 confirms the clinical impact of these gene expressions, where elevated levels of *UBE2C* correlated with reduced survival rates. This suggests that the overexpression of these genes, which are involved in the ubiquitin-proteasome system, might contribute to more aggressive cancer behavior and poorer prognosis. In addition, the consistent upregulation of *PSMA1* and *UBE2C* across multiple analyses suggests an association with pancreatic cancer progression, positioning these genes as hypothesis-generating candidates for prognostic and therapeutic targets. Their association with poor survival outcomes warrants further investigation into how they can be targeted to improve treatment strategies, namely additional validation across independent patient cohorts, assay standardization, clinically useful cutoff definition, and multivariable testing against established clinicopathologic predictors. Overall, the therapy proposed here could offer a two-pronged approach to treating pancreatic cancer: direct cytotoxicity through improved delivery of GEM and potential immunomodulatory effects that could enhance defense mechanisms against both peritoneal tumor implants and metastatic tumor deposits.^61^ This could represent a significant advancement in the management of difficult-to-treat cancers, such as pancreatic cancer. Our data provide a molecular basis for the observed clinical efficacy of this therapy and further our understanding of its mechanisms of action, however further validation with functional assays is needed to confirm their direct contribution to therapeutic efficacy.

## Conclusion

Despite the advancements in cancer therapeutics, the outlook for pancreatic cancer remains poor. The complexity of the tumor microenvironment, coupled with the strong desmoplastic reaction, has historically rendered systemic chemotherapy regimens only modestly effective.^19^ However, nanotechnology and materials science in drug delivery have opened promising paths to bypass these obstacles.^62^ Our current research underscores the potential of self-immolative micelles as a versatile platform that can transform pancreatic cancer treatment, enhancing both efficacy and tolerability of chemotherapeutics.^63^

The synergistic combination of PTX and GEM within a single self-assembled micelle carrier within a hydrogel not only amplifies the cytotoxic effects against peritoneal tumor implants but also exhibits pronounced activity against metastatic tumor deposits. This dual-drug strategy leverages the unique pharmacological profiles of each agent, with the nanocarrier ensuring harmonized delivery to the tumor site. We did not include a micelle-only control group, conclusions are therefore framed around hydrogel-enabled local delivery. As a result, the independent contribution of the micellar carrier to the observed therapeutic effects cannot be fully isolated from the retention and release properties conferred by the hydrogel matrix. Our findings is that the micelles primarily contribute payload protection, triggered intracellular release, cellular uptake, and intracellular retention, whereas the hydrogel contributes locoregional retention, prolonged peritoneal residence, and depot-mediated exposure. A prospectively powered micelle-only comparison will be needed in future work to isolate the incremental contribution of the hydrogel depot. The modular nature of these nanocarriers holds the potential for encapsulating an array of drugs, including novel small-molecule inhibitors and emerging targeted therapies. Such versatility could enhance personalized medical applications in pancreatic cancer, where treatment regimens are fine-tuned to the patient’s specific genetic and molecular tumor profile. Moreover, the EPR effect observed with our NP system combined with a hydrogel scaffold^29^ as a micelle depot through locoregional intraperitoneal delivery, together with prolonged hydrogel-mediated residence, improves exposure of peritoneal tumor foci and metastatic implants. Once in contact with tumor cells, the micellar formulation supports intracellular delivery and retention. This is particularly beneficial in pancreatic cancer, given the dense stromal environment that often impedes drug penetration.^64^ Future studies employing genetically engineered PDAC models. Orthotopic, or patient-derived xenograft models with stromal characterization will be necessary to assess whether the platform can overcome the ECM-rich microenvironment that constitutes a central barrier to drug delivery in pancreatic cancer. The true demonstration of stromal barrier overcoming would require dedicated penetration studies and ECM analyses.Future iterations of these prodrug micelles might incorporate stroma-modulating agents that could further augment drug delivery efficacy. Also, because the combination arm partitions payload between drugs at a fixed ratio while holding total depot constant, it is not an equi-dose comparison versus monotherapies; ratio/schedule optimization will be addressed in future work. Our comparison is not designed to enforce equi-dose in a pharmacokinetic sense, but rather equi-effect at the single-agent level, which is a meaningful and commonly used benchmark in early stage evaluation. The combination responses are inherently dose-ratio dependent and nonlinear, and that IC_50_ normalization does not completely eliminate these complexities. Direct equi-mass dose comparisons and full pharmacokinetic profiling represent important directions for future investigation to further characterize the quantitative contribution of each agent within the combination platform. Future work is focused on expanding the analysis provided in a more comprehensive and balanced assessment of combination behavior as well as expanding on the learnings from the genomic analysis of this study.

The genetic profiling analysis in our study offers valuable insights into the molecular mechanisms of the tumor response to micelle embedded hydrogel therapy. Integrating these findings with global gene expression databases could help identify biomarkers predictive of treatment response and novel targets for codelivery with existing chemotherapeutic agents. In fact, we observed a modulation of critical pathways such as PI3K-Akt, MAPK, and VEGF signaling by these treatments, highlighting the potential for targeted therapies that can disrupt the signaling networks necessary for tumor survival, proliferation, and dissemination of cancer cells and their dysregulation as a hallmark of cancer progression and chemoresistance.^65^ The integration of our findings with clinical data from GEO, GTex, TCGA, and TARGET databases has provided valuable insights into the role of ubiquitination and proteasome degradation pathways in pancreatic cancer. It is important to note that the integration of publicly available transcriptomic databases provides cross-data set associative corroboration of the gene expression patterns observed in our model but does not establish mechanistic causality within our specific experimental system. The frequent genetic alterations in key genes such as *RNF123* and *PSMA1*, as revealed by oncoprint visualization, offer a genetic backdrop to disease progression. Moreover, the substantial upregulation of *UBE2C* in metastatic tumors suggests that this gene may be a potential biomarker for advanced disease stages. Survival analyses further established the clinical significance of these pathways, linking high expression levels of genes such as *UBE2C* to poorer patient outcomes. Our current data set supports association, not formal causality. The transcriptomic analysis presented here should be interpreted as exploratory, identifying candidate pathways and gene targets for hypothesis-driven follow-up investigation. To establish causality more rigorously, future work would require earlier sampling before major tumor regression, direct pathway-level protein or phospho-protein analyses, and perturbation experiments testing whether reversing these gene changes alters therapeutic response. Moreover, independent validation of key DEGs by orthogonal methods such as RT-PCR, and replication in an independent in vivo cohort, represent important priorities for future work to confirm and extend these findings.

These collective insights underscore the ubiquitination and proteasome pathways as key to the understanding and treatment of pancreatic cancer, highlighting their relevance in the development of targeted therapies and prognostic evaluations. The modulation of these genes and pathways by micelle-encapsulated drugs presents a case for the development of targeted therapies in pancreatic cancer, aiming not only to inhibit peritoneal tumor implant tumor growth but also to prevent metastatic spread. Understanding these alterations at the genomic level is key to advancing therapeutic strategies and improving the prognosis of patients with pancreatic cancer. In terms of clinical translation, these findings serve as a preclinical foundation, warranting progression to clinical trials.

## Supplementary Material



## Data Availability

Data are available in the Supporting Information and on request from the authors.

## Notes

General synthetic procedures and characterization of intermediates and final compounds (NMR and LC-MS); micelles and hydrogel fabrication protocols including preparation of micelle@GEM, micelle@PTX, rhodamine-loaded and naked micelles; surface morphology, size, stability, and UV–vis quantification; LC-HRMS analysis of GSH-triggered release, CDA metabolism, and drug release from hydrogel formulations; rheological behavior and microstructure of hyaluronic acid hydrogel; dosing rationale. Additional results and discussion of micelles characterization, in vitro cell studies, hyaluronic acid hydrogel characterization, and in vivo study. (PDF)
