# Supplementary material for: In Vivo Multimodal Self-Immolative Prodrug Nanomicelle-Hydrogel Modulate Key Intratumoral and Metastatic Genomic Signaling Pathways in Pancreatic Cancer
Source: ACS Nano Med. 2026 Jul 7;1(7):1640–58. doi: 10.1021/acsnanomed.5c00174 (PMC13501496; doi:10.1021/acsnanomed.5c00174)
Supplement: Supplementary file 1 [file nm5c00174_si_001.pdf]

SUPPORTING INFORMATION

In vivo multimodal self-immolative prodrug nanomicelle-hydrogel modulate key intratumoral and metastatic genomic signalling pathways in pancreatic cancer

João M. J. M. Ravasco[1,2], Jhenifer Oliveira[1,2], João Conniot[1,2], Bárbara B. Mendes[1,2], Catarina Leal[3,4], Susete N. Fernandes[3], Carlos A. M. Afonso[5], João Conde[1,2,*]

[1] NOVA Medical School|Faculdade de Ciências Médicas, NMS|FCM, Universidade NOVA de Lisboa, 1169-056, Lisbon, Portugal.

[2] Comprehensive Health Research Centre (CHRC), NOVA Medical School|Faculdade de Ciências Médicas, NMS|FCM, Universidade NOVA de Lisboa, 1169-056 Lisbon, Portugal

[3] i3N/CENIMAT, Department of Materials Science, NOVA School of Science and Technology, Universidade NOVA de Lisboa, Campus de Caparica, 2829-516, Caparica, Portugal.

[4] Departamento de Física, Instituto Superior de Engenharia de Lisboa, Instituto Politécnico de Lisboa, 1549-020, Portugal.

[5] Instituto de Investigação do Medicamento (iMed), Faculdade de Farmácia, Universidade de Lisboa, Av. Prof. Gama Pinto, 1649-003 Lisboa, Portugal.

* Corresponding author: joao.conde@nms.unl.pt

1. General synthetic procedures

All solvents were of analytical reagent grade. DCM, Toluene, methanol, and ethyl acetate were of analytical reagent grade and were purchased from Carlo Erba and distilled prior to used. DMF (anhydrous, ≥99.8%; Product Code 5895651000, CAS 68-12-2) was purchased from Sigma Aldrich and used as is.

Gemcitabine hydrochloride (98% pure; Product Code F079926; CAS 122111-03-9), alpha-tocopherol (98% pure; Product Code F094655; CAS 10191-41-0), 4-Nitrophenyl chloroformate (90.0% purity; Product Code F038189; CAS 7693-46-1) were purchased from Fluorochem. DMAP 4-(Dimethylamino)pyridine (≥ 99,0 % purity; 107700, CAS 1122-58-3), Succinic anhydride (≥ 98,0 % purity; 8006830500; CAS 108-30-5), 2,2'-disulfanediylbis(ethan-1-ol) (technical grade; 380474; CAS 1892-29-1), EDC hydrochloride (≥98.0% Product Code 03450, CAS 25952-53-8) were purchased from Sigma-Aldrich. Triethylamine (99% purity; Product Code A12646, CAS 121-44-8) was purchased from Alfa Aesar and distilled prior to use.

Liquid nuclear magnetic resonance spectroscopy (NMR). NMR spectra were recorded on a Bruker Fourier 300 spectrometer using CDCl3, (CD3)2CO, D2O or (CD3)2SO as deuterated solvents. All coupling constants are expressed in Hz, and chemical shifts (δ) are expressed in ppm. Multiplicities are given as: s (singlet), d (doublet), dd (double doublet), dt (double triplet), t (triplet), td (triple triplet), tt (triple triplet), q (quartet), quint (quintuplet) and m (multiplet). IR spectra were recorded using a Bruker FTIR-ATR Alpha II spectrometer. The UV spectra were recorded using a Thermo Scientific Evolution 201 UV-visible spectrophotometre. Fluorescence measurements were performed using a SHIMADZU spectrofluorophotometer RF-6000 instrument. Low-resolution mass spectra were recorded using an LCQ Fleet Ion Trap Mass Spectrometer (Thermo Fisher Scientific, Germany). High-resolution mass spectra were recorded on a Thermo Scientific Q Exactive hybrid quadrupole-Orbitrap mass spectrometer (Thermo Scientific^{TM} Q Exactive^{TM} Plus).

Liquid chromatography–mass spectrometry (LC-MS). LC-MS runs were performed using a Dionex Ultimate 3000 UHPLC+ system equipped with a Multiple-Wavelength detector, using an imChem Surf C18 TriF100A 3 μm 100×2,1 mm column connected to a Thermo Scientific Q Exactive hybrid quadrupole-Orbitrapmass spectrometer (Thermo ScientificTM Q ExactiveTM Plus).

Synthesis of α-tocopherol succinate (TOS).

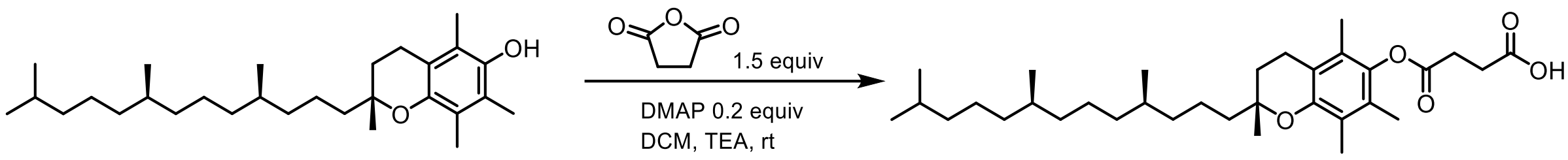

Fig. S1. Synthesis of TOS.

In a round-bottomed flask, α-tocopherol (4.01 g, 9.31 mmol, 1 equiv), was dissolved in in anhydrous DCM (60 mL), to which was sequentially added triethylamine (1.94 mL, 13.97 mmol), DMAP (0.28 g, 2.83 mmol) and succinic anhydride (1.40 g, 13.99 mmol). The mixture was stirred at room temperature for 2 h under a nitrogen atmosphere, past which time was washed with 5% HCl solution (3×20mL) and brine (1×20mL), dried over anhydrous Na_2SO_4, filtered, and concentrated in vacuum. The residue was redissolved in 5.0 mL cold DCM, after which the solution was filtered to remove excess SA and concentrated to provide α-tocopherol succinate (α-tocopherol succinate) (4.31 g, ~99% yield), which was used without any further purification.

^{1}H NMR (300 MHz, Chloroform-*d*) δ 2.95 – 2.91 (m, 2H), 2.85 – 2.80 (m, 2H), 2.63 – 2.55 (m, 2H), 2.09 (s, 3H), 1.99 (d, *J* = 12.8 Hz, 6H), 1.87 – 1.69 (m, 2H), 1.60 – 1.47 (m, 3H), 1.46 – 1.33 (m, 5H), 1.32 – 1.20 (m, 9H), 1.19 – 1.00 (m, 7H), 0.94 – 0.81 (m, 12H).

^{13}C NMR (75 MHz, Chloroform-*d*) δ 178.14, 170.82, 149.48, 140.45, 126.69, 124.94, 123.06, 117.42, 75.07, 39.39, 37.55, 37.47, 37.40, 37.30, 32.75, 28.97, 28.59, 27.99, 24.82, 24.45, 22.72, 22.63, 21.04, 20.60, 12.87, 12.02, 11.81.

HRMS: calcd $[M+H]^+$ = 531.4044; found $[M+H]^+$ = 531.4051

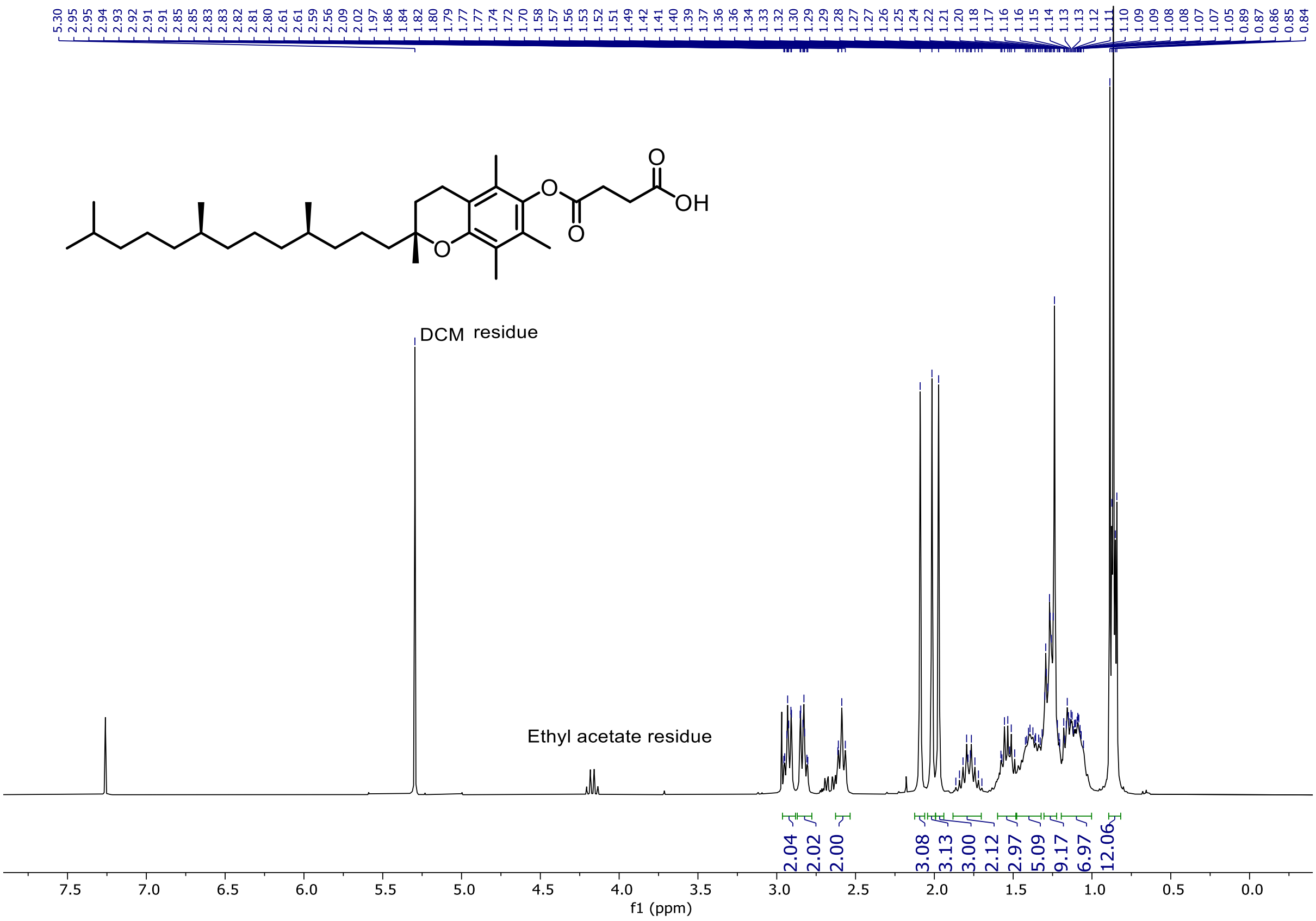

Fig. S2. ^{1}H NMR spectrum of TOS.

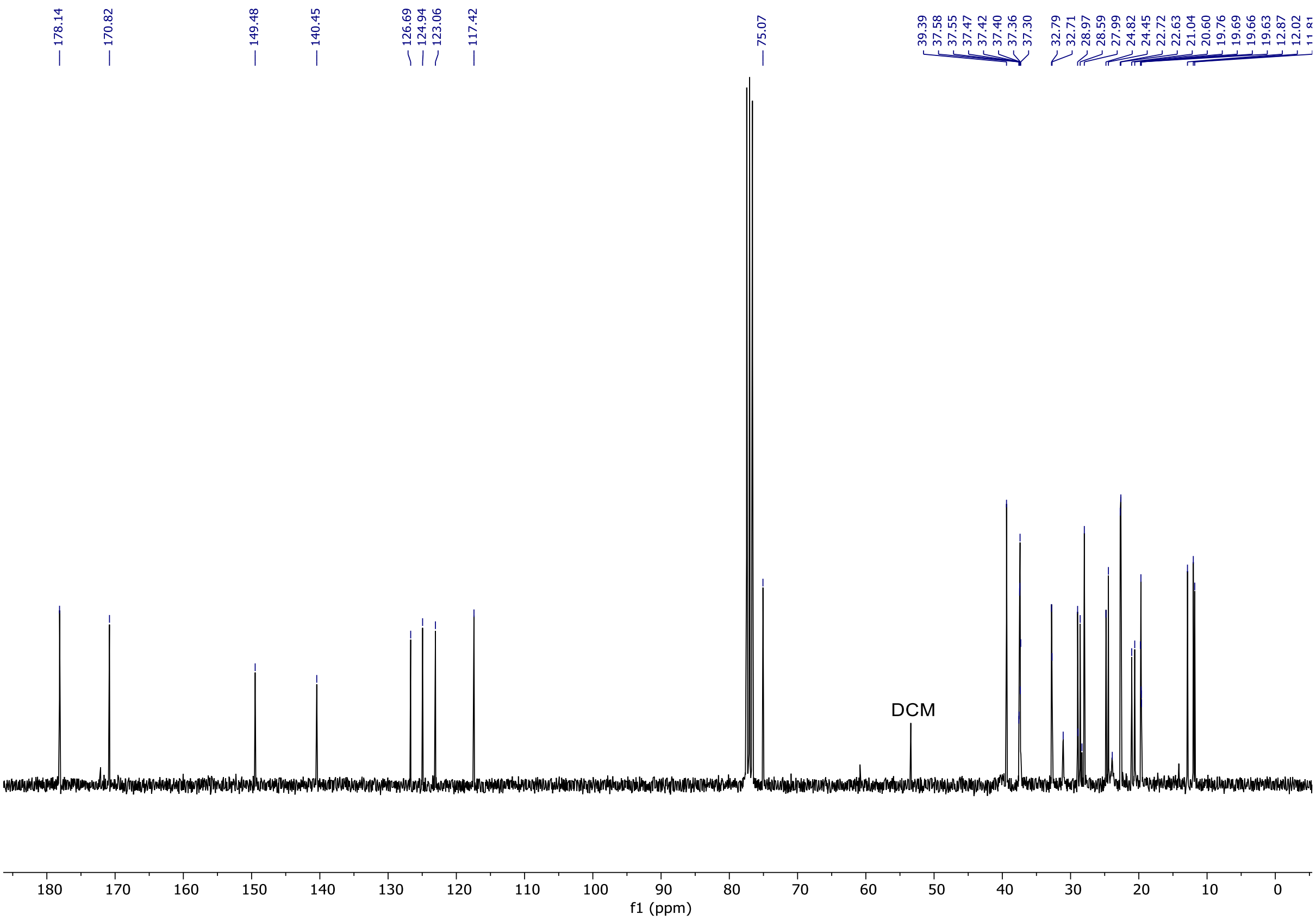

Fig. S3. ^{13}C NMR spectrum of TOS.

Synthesis of TOS-SSOH.

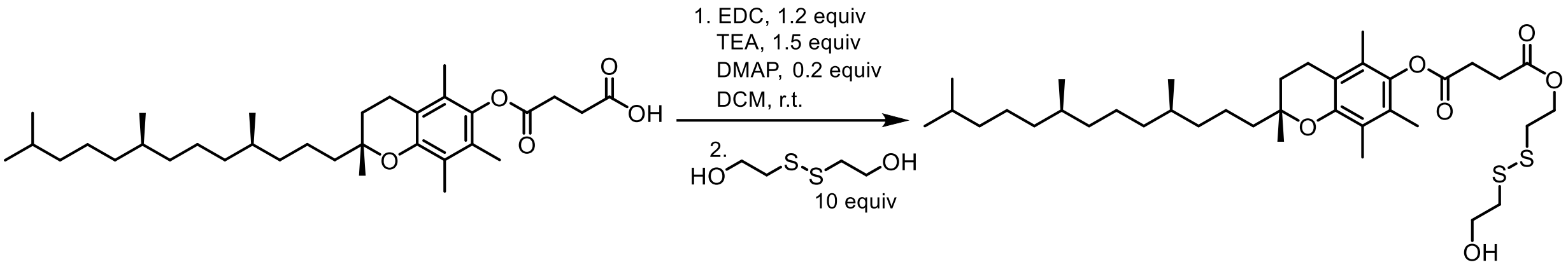

Fig. S4. Synthesis of TOS-SSOH.

In a round-bottomed flask, α-TS (4.31 g, 7.31 mmol, 1 equiv), was dissolved in in anhydrous DCM (75 mL), to which was sequentially added EDC hydrochloride (1.59 g, 8.29 triethylamine (2.97 mL, 11.3 mmol), and DMAP (0.184 g, 1.5 mmol). The mixture was stirred at room temperature for 30 min under a nitrogen atmosphere, and 2,2'-disulfanediylbis(ethan-1-ol) (5.81 g, 37.68 mmol) was added. The mixture was stirred overnight, past which time was washed with a saturated $NaHCO_3$ solution (2×20mL) and brine (1×20mL), dried over anhydrous Na_2SO_4, filtered, and concentrated under vacuum. The residue was purified by column chromatography using a gradient of 8:2 to: 7:3 hexane/ethyl acetate to obtain the product as a pale-yellow oil (3.57g, 71% yield).

^{1}H NMR (300 MHz, Chloroform-*d*) δ 4.41 – 4.36 (m, 2H), 3.88 – 3.78 (q, *J* = 5.8 Hz, 2H), 3.03 – 2.90 (m, 4H), 2.86 (t, *J* = 5.9 Hz, 2H), 2.81 – 2.74 (m, 2H), 2.61 – 2.53 (m, 2H), 2.08 (s, 3H), 1.99 (d, *J* = 12.1 Hz, 7H), 1.86 – 1.69 (m, 2H), 1.62 – 1.47 (m, 2H), 1.46 – 1.33 (m, 5H), 1.32 – 1.20 (m, 9H), 1.20 – 0.99 (m, 7H), 0.93 – 0.78 (m, 12H).

^{13}C NMR (75 MHz, Chloroform-*d*) δ 172.10, 171.01, 149.47, 140.43, 126.64, 124.90, 123.06, 117.43, 75.08, 62.68, 60.34, 41.57, 39.38, 37.55, 37.46, 37.39, 37.29, 36.94, 32.79, 31.07, 29.05, 28.76, 27.98, 24.81, 24.44, 22.73, 22.63, 21.04, 20.60, 19.76, 19.70, 19.66, 19.63, 19.60, 12.94, 12.10, 11.81.

HRMS: calcd $[M+H]^+$ = 667.4061; found $[M+H]^+$ = 667.4067

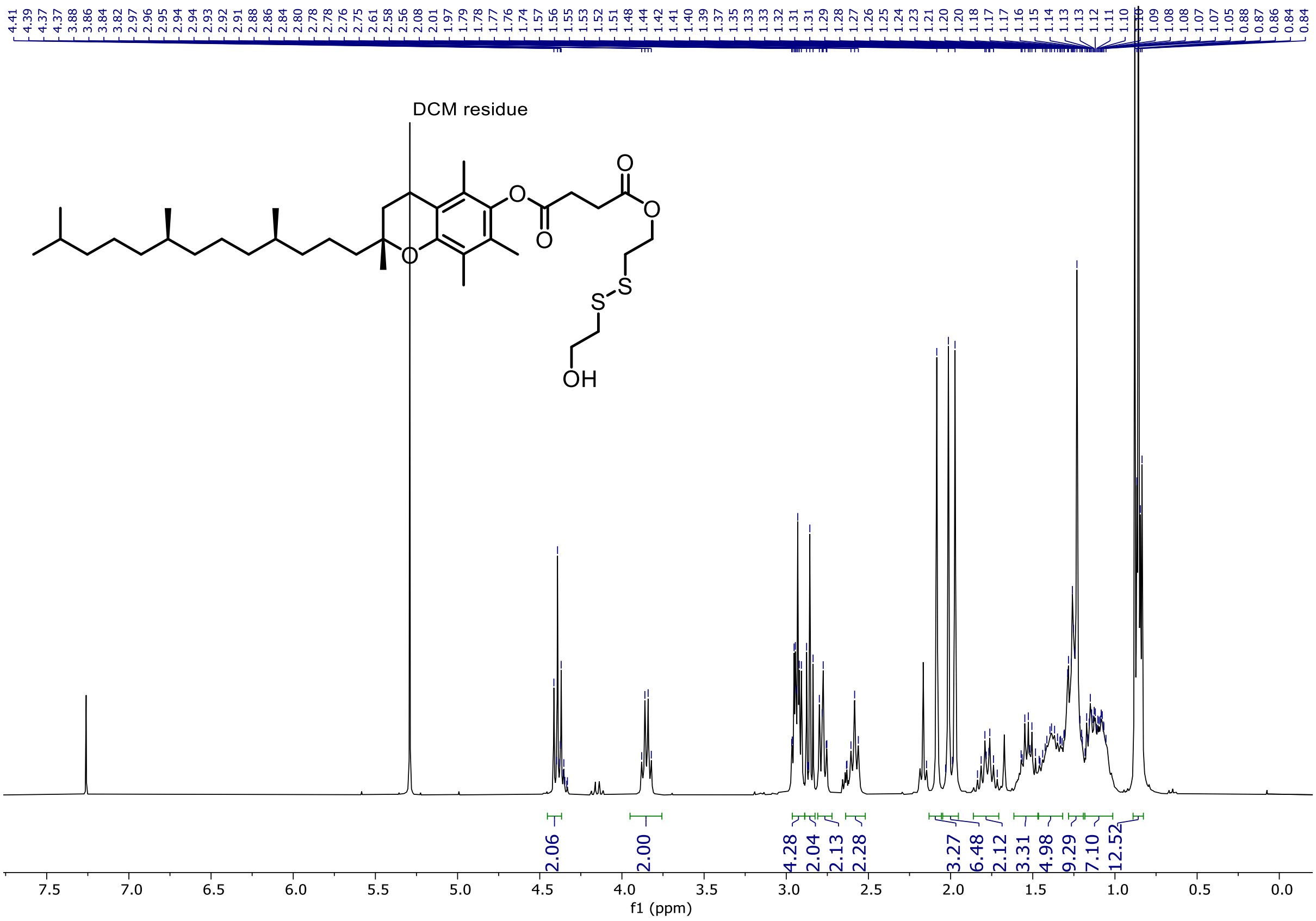

Fig. S5. ^{1}H NMR spectrum of TOS-SSOH.

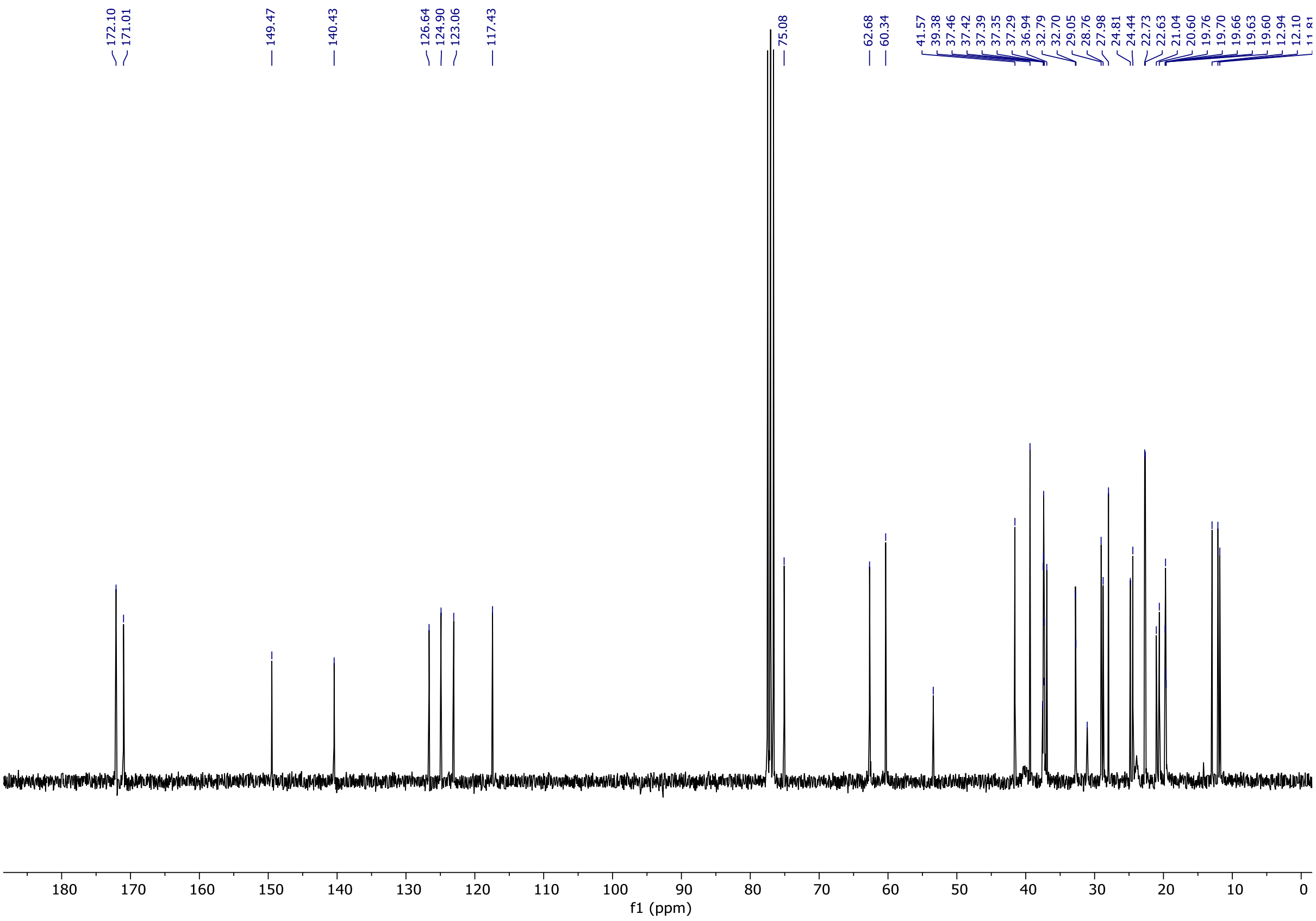

Fig. S6. ^{13}C NMR spectrum of TOS-SSOH.

Synthesis of Toco_OSSOH_PNB.

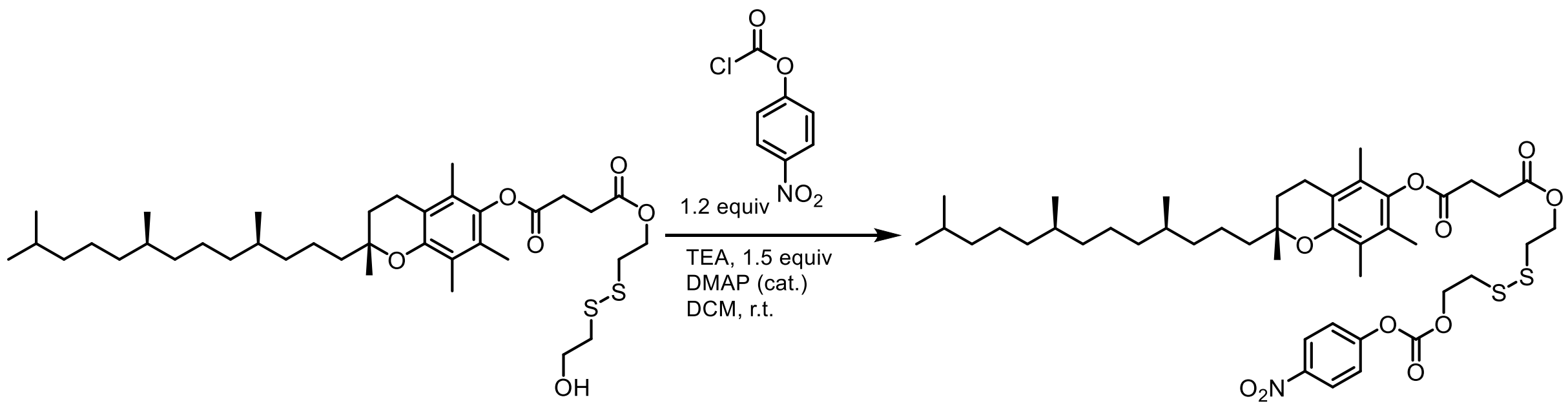

Fig. S7. Synthesis of Toco_OSSOH_PNB.

In a round-bottomed flask, α-TSOH (500 mg, 0.7 mmol, 1 equiv), was dissolved in in anhydrous DMF (8 mL), to which was sequentially added 4-Nitrophenyl chloroformate (181 mg, 0.9 mmol) and triethylamine (157 µL, 1.13 mmol) and catalytic DMAP. The mixture was stirred at room temperature for 3h under nitrogen atmosphere, past which time was washed with brine (3×20mL), dried over anhydrous Na_2SO_4, filtered, and concentrated under vacuum. The residue was purified by column chromatography using 98:2 toluene/ethyl acetate as eluent to afford the product as a pale-yellow solid (573 mg, 92% yield).

Note: The product is unstable in water and acidic environments and should be stored under a dry nitrogen atmosphere.

^{1}H NMR (300 MHz, Chloroform-*d*) δ 8.27 (d, *J* = 9.1 Hz, 2H), 7.38 (d, *J* = 9.1 Hz, 2H), 4.54 (t, *J* = 6.5 Hz, 2H), 4.40 (t, *J* = 6.6 Hz, 2H), 3.03 (t, *J* = 6.5 Hz, 2H), 2.95 (q, *J* = 6.9 Hz, 4H), 2.85 – 2.74 (m, 2H), 2.58 (t, *J* = 6.8 Hz, 2H), 2.08 (s, 3H), 1.99 (d, *J* = 12.3 Hz, 6H), 1.84 – 1.67 (m, 2H), 1.60 – 1.46 (m, 3H), 1.47 – 1.32 (m, 5H), 1.31 – 1.19 (m, 11H), 1.19 – 1.00 (m, 7H), 0.92 – 0.79 (m, 12H).

^{13}C NMR (75 MHz, Chloroform-*d*) δ 172.03, 171.02, 155.40, 152.30, 149.49, 145.48, 140.42, 126.62, 125.32, 124.89, 123.09, 117.44, 115.51, 75.10, 66.82, 62.56, 39.38, 37.57, 37.46, 37.39, 37.29, 37.08 (d, *J* = 2.0 Hz), 36.76, 32.79, 32.69, 29.04, 28.76, 27.98, 24.82, 24.44, 22.73, 22.63, 21.04, 20.59, 19.76, 19.69, 19.65, 19.62, 19.59.

HRMS: calcd $[M+H]^+$ = 832.4123; found $[M+H]^+$ = 832.4121

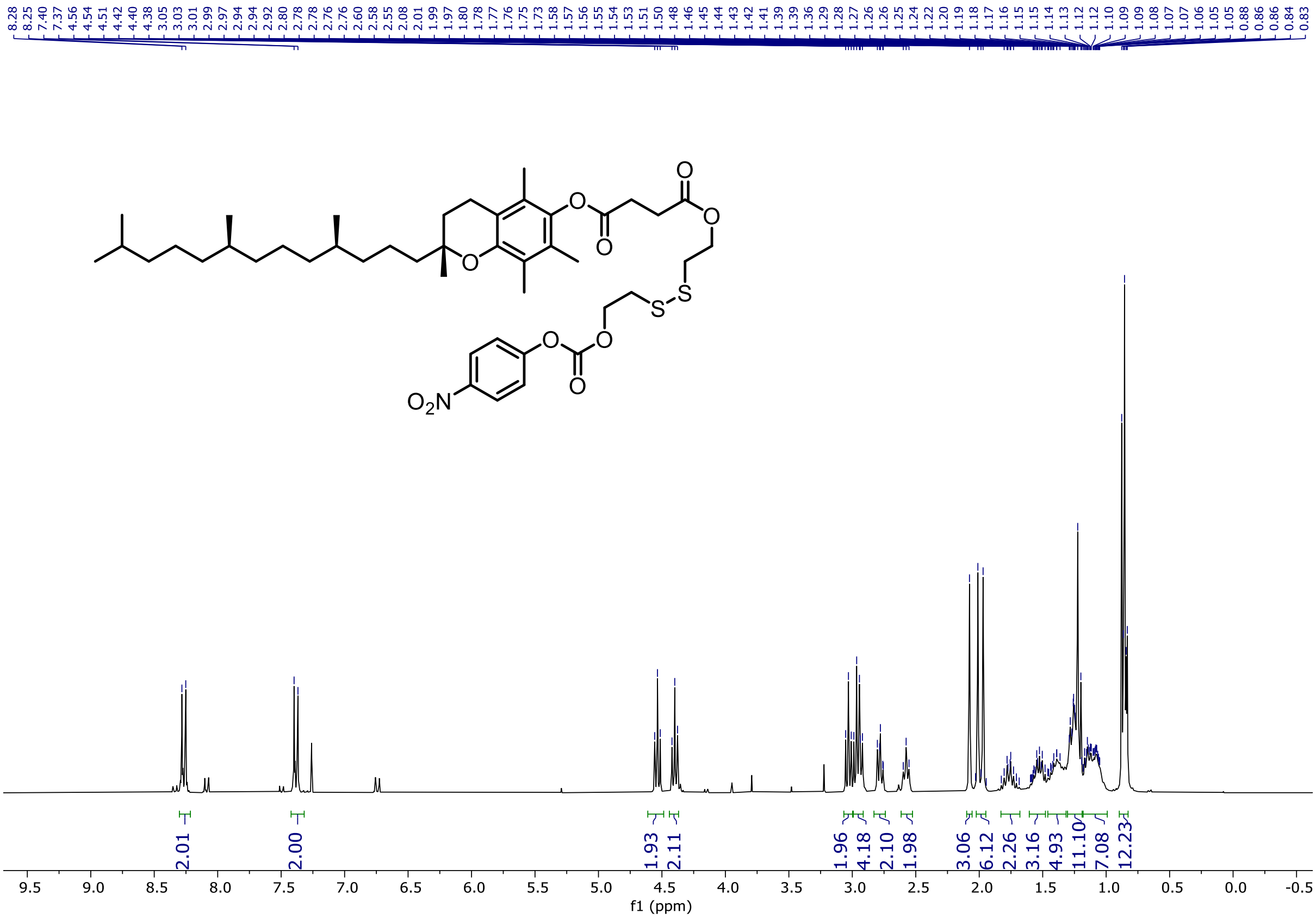

Fig. S8. ^{1}H NMR spectrum of Toco_OSSOH_PNB.

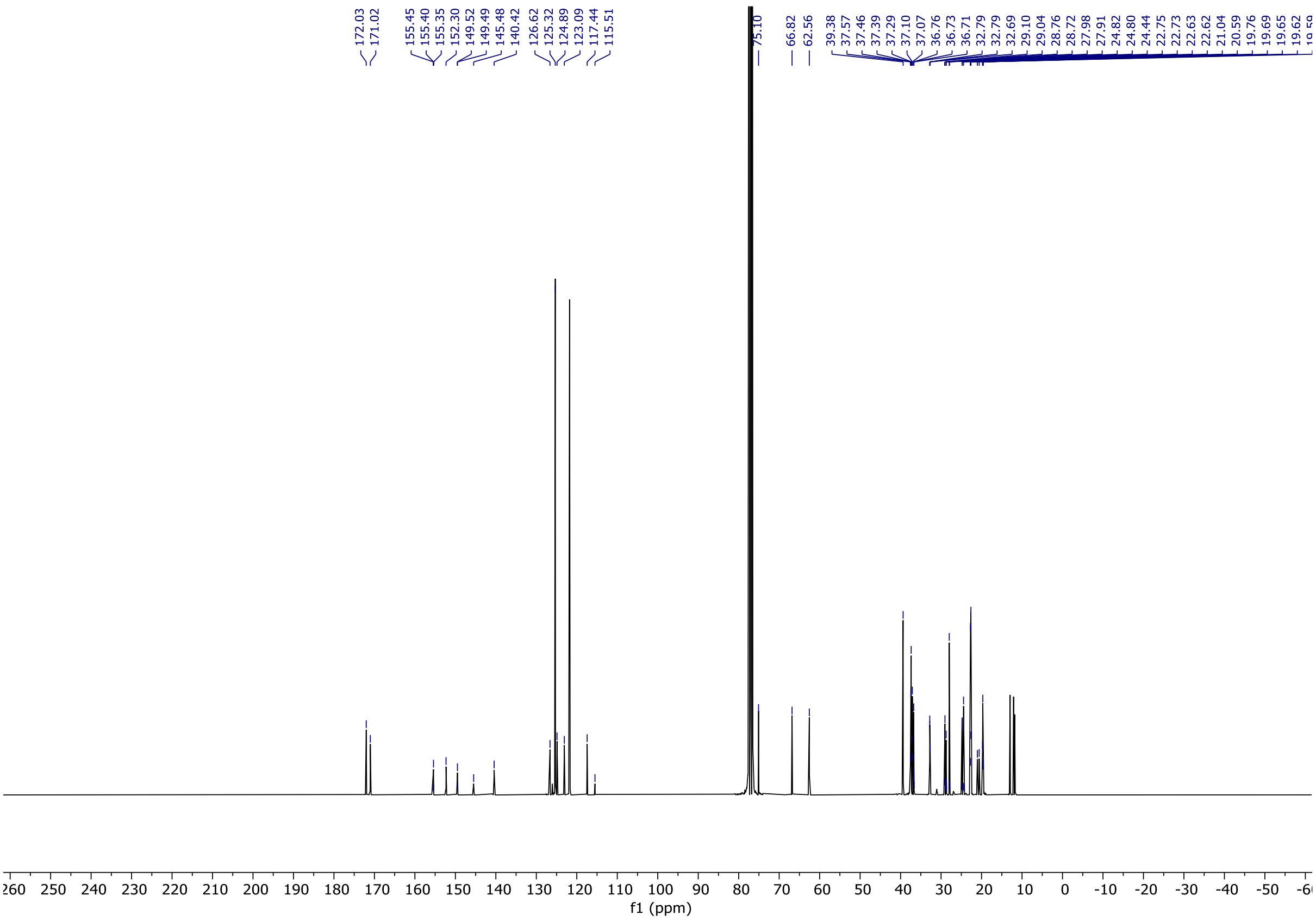

Fig. S9. ^{13}C NMR spectrum of Toco_OSSOH_PNB.

Synthesis of TOS-SS-GEM.

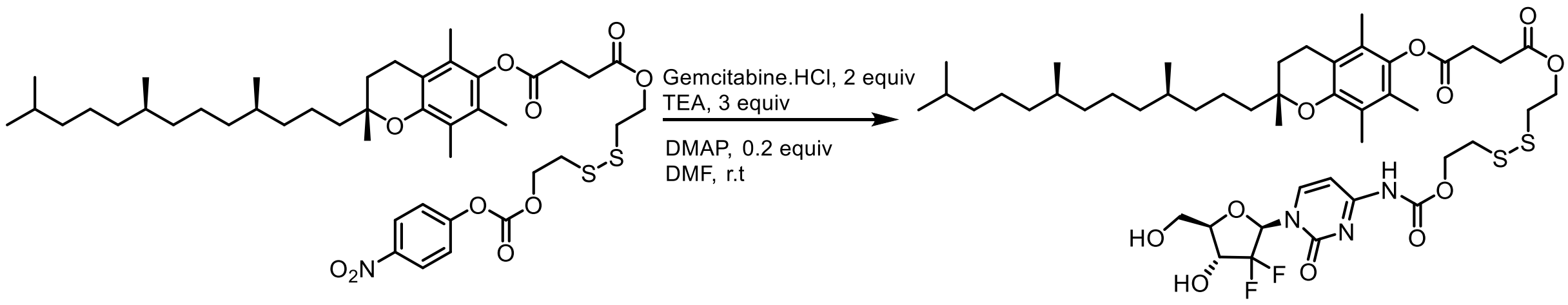

Fig. S10. Synthesis of TOS-SS-GEM.

In a round-bottomed flask, α-TSOPNB (360 mg, 0.43 mmol, 1 equiv), was dissolved in anhydrous DMF (2.8 mL), to which was sequentially added gemcitabine hydrochloride (181 mg, 0.87 mmol), triethylamine (300 µL, 2.16 mmol) and catalytic DMAP. The mixture was stirred at room temperature for 24h under a nitrogen atmosphere. Prior to the evaporation of DMF under vacuum, the crude product was diluted in water and extracted with DCM (3×15 mL). The organic phase was dried over anhydrous Na_2SO_4, filtered, and concentrated in vacuum. The residue was purified by column chromatography using 100:0 to 87.5:12.5 DCM/methanol as eluent to afford the product as a pale yellow solid (219 mg, 53% yield).

^{1}H NMR (300 MHz, Chloroform-*d*) δ 7.48 (d, *J* = 7.5 Hz, 1H), 6.20 (t, *J* = 8.6 Hz, 1H), 5.79 (d, *J* = 7.5 Hz, 1H), 5.36 (q, *J* = 9.1 Hz, 1H), 4.45 (t, *J* = 6.5 Hz, 2H), 4.36 (t, *J* = 6.5 Hz, 2H), 4.12 (d, *J* = 7.5 Hz, 1H), 4.03 (d, *J* = 12.4 Hz, 1H), 3.79 (d, *J* = 12.4 Hz, 1H), 3.02 – 2.86 (m, 6H), 2.77 (m, 2H), 2.57 (t, *J* = 6.7 Hz, 2H), 2.07 (s, 3H), 1.98 (d, *J* = 12.0 Hz, 6H), 1.83 – 1.68 (m, 2H), 1.60– 1.46 (m, 3H), 1.44– 1.30 (m, 5H), 1.29– 1.15 (m, 11H), 1.16 – 1.02 (m, 7H), 0.88 – 0.81 (m, 12H).

^{13}C NMR (75 MHz, Chloroform-*d*) δ 172.12, 171.08, 166.00, 155.94, 153.56, 149.45, 140.43, 126.65, 124.94, 123.03, 117.45, 75.11, 66.86, 62.58, 50.68, 39.37, 37.42, 37.40, 37.28, 36.98, 36.58, 32.78, 32.70, 31.07, 29.03, 28.76, 27.97, 24.80, 24.44, 21.03, 20.58, 19.75, 19.68, 19.64, 12.93, 12.08, 11.80.

HRMS: calcd $[M+H]^+$ = 956.4571; found $[M+H]^+$ = 956.4580

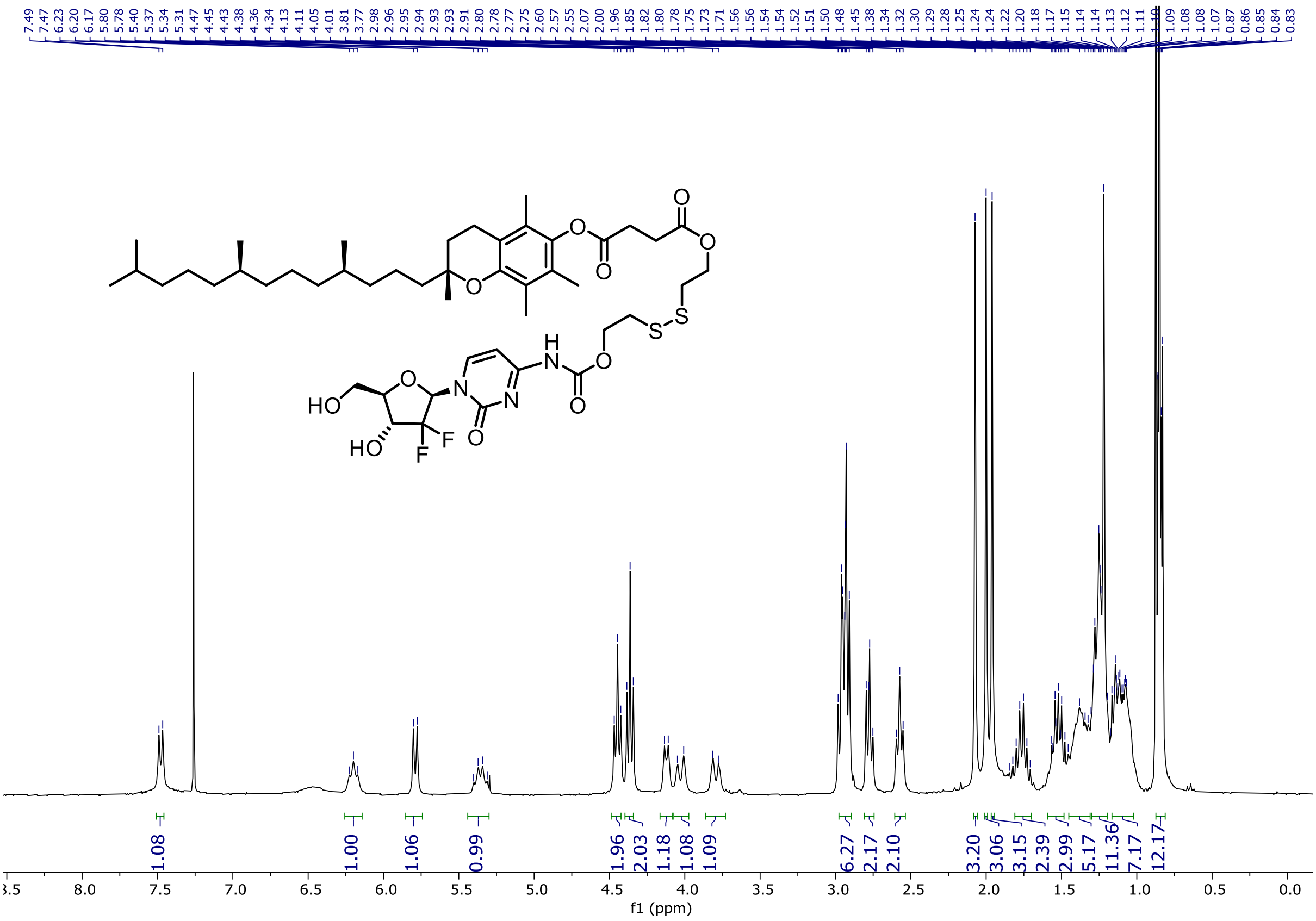

Fig. S11. ^{1}H NMR spectrum of TOS-SS-GEM.

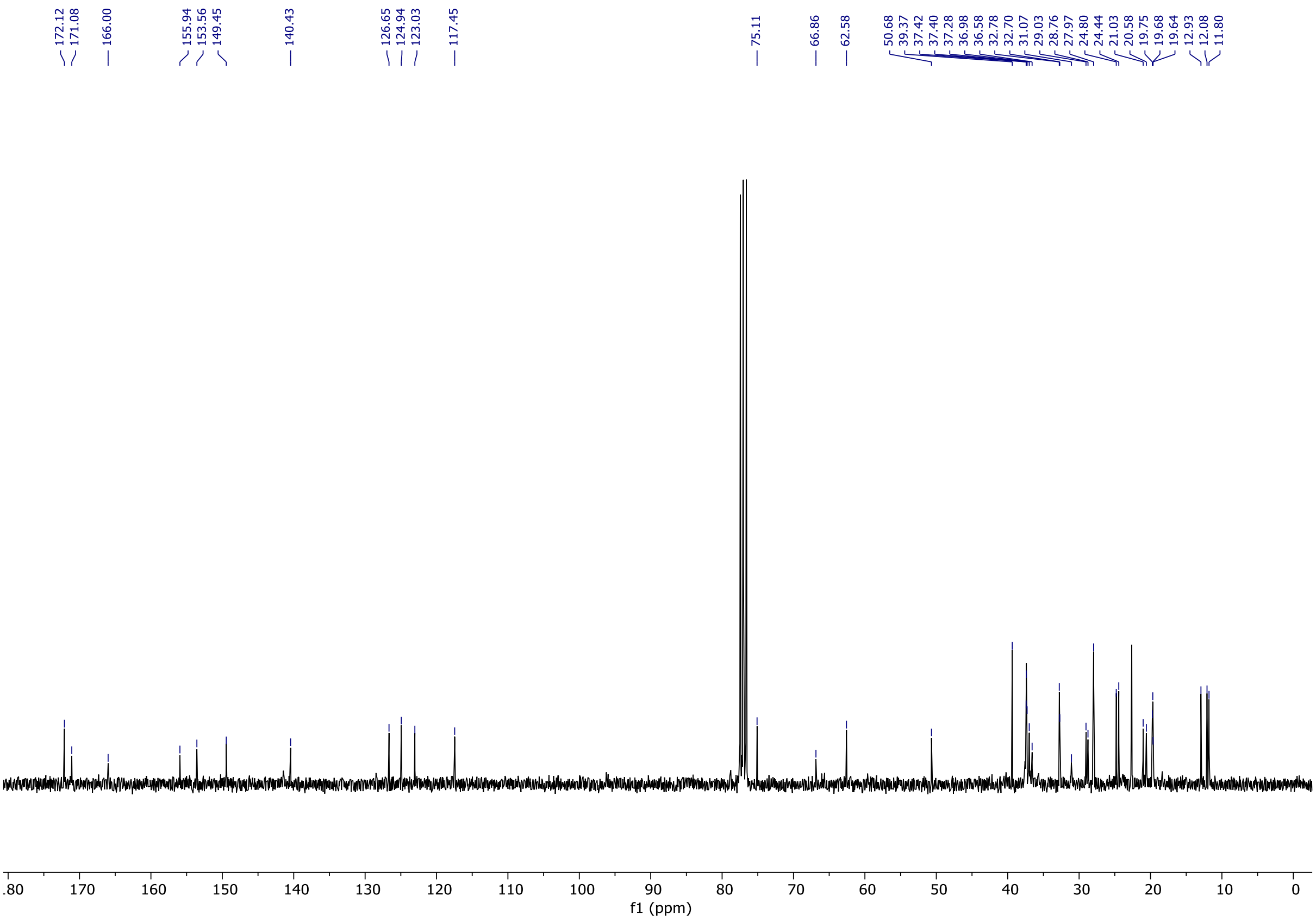

Fig. S12. ^{13}C NMR spectrum of TOS-SS-GEM.

2. Micelles and hydrogel fabrication and characterization.

Preparation of micelle@GEM. Micelle@GEM was prepared using a simple solvent evaporation method with minor modifications. Briefly, 12 mg of TPGS was solubilized in 1.5 ml of ethanol and used to dissolve 18 mg of TOS-SS-GEM. The 1.5 mL solution of both components was added dropwise to 15 ml of ultra-purified water or PBS buffer under stirring (600 rpm). The solution was stirred on a magnetic stirrer overnight to evaporate the organic solvent and characterised by DLS, TEM, and SEM. For storage, micelles were kept refrigerated at 4°C until further use. Each batch was characterised using DLS. Physicochemical stability was assessed by DLS analysis of 6 independent batches, 3 kept at room temperature, and 3 at 4°C. The particles were stable for at least 4 weeks under either condition.

Preparation of rhodamine-loaded micelle@GEM. Similarly, rhodamine-loaded micelle@GEM was prepared using a simple solvent evaporation method, with minor modifications. Briefly, 12 mg of TPGS was solubilized in 1.5 ml of ethanol and used to dissolve 18 mg of TOS-SS-GEM and 1 mg of rhodamine (30% w/w). The 1.5 mL solution of all components was added dropwise to 15 ml of ultra-purified water or PBS buffer under stirring (600 rpm) was left evaporating overnight. For storage, micelles were kept refrigerated at 4°C until further use. Each batch was characterised using DLS. Physicochemical stability was assessed by DLS analysis of 6 independent batches, 3 kept at room temperature, and 3 at 4°C. The particles were stable for at least 4 weeks under either condition.

Preparation of PTX-loaded micelle (micelle@PTX). Similarly, PTX-loaded micelle was prepared using a simple solvent evaporation method, with minor modifications. Briefly, 12 mg of TPGS was solubilized in 1.5 ml of ethanol and used to dissolve 18 mg of TOS-SS-GEM and 1 mg of PTX (30% w/w). The 1.5 mL solution of all components was added dropwise to 15 ml of ultra-purified water or PBS buffer under stirring (600 rpm) was left evaporating overnight. For storage, micelles were kept refrigerated at 4°C until further use. Each batch was characterised using DLS. Physicochemical stability was assessed by DLS analysis of 6 independent batches, 3 kept at room temperature, and 3 at 4°C. The particles were stable for at least 4 weeks under either condition.

Preparation of naked micelles. Similarly, naked micelles were prepared using a simple solvent evaporation method, with minor modifications. Briefly, 12 mg of TPGS was solubilized in 1.5 ml of ethanol and used to dissolve 18 mg of TOS-SS-GEM. The 1.5 mL solution of all components was added dropwise to 15 ml of ultra-purified water or PBS buffer under stirring (600 rpm) and ethanol was left evaporating overnight. For storage, micelles were kept refrigerated at 4°C until further use. Each batch was characterised using DLS. Physicochemical stability was assessed by DLS analysis of 6 independent batches, 3 kept at room temperature, and 3 at 4°C. The particles were stable for at least 4 weeks under either condition.

Surface morphology, size, and size distribution. Micelles were characterised using a Malvern Zetasizer Nano ZSP, Malvern Panalytical Ltd (Malvern, United Kingdom), which utilises dynamic light scattering with a non-invasive backscatter (NIBS) optics technique, to determine the particle size and zeta potential of the prepared micelles. Measurements for each sample were performed in triplicate.

Particle stability. For storage, micelles were kept refrigerated at 4°C until further use. Each batch was characterised using DLS. Physicochemical stability was assessed by DLS analysis of 6 independent batches, 3 kept at room temperature, and 3 at 4°C. The particles were stable for at least 4 weeks under either condition.

LC-HRMS analysis of GSH-triggered release. From 2 mg/mL micelle@GEM, solutions with a final concentration of 50 μM TOS-GEM were incubated in PBS (pH 7.4, 37°C) with different concentrations of GSH (ranging from 0 to 10mM). The progression of each reaction solution was followed by LC- HRMS in Positive Mode. The HPLC runs were carried out with a gradient of A (Milli Q water containing 0.1% v/v Formic acid, FA) and B (acetonitrile containing 0.1% v/v FA, Honeywell HPLC-grade). The mobile phase was t = 0 min, 5% B; t = 5 min, 5% B; t = 18 min, 95% B; t = 22 min, 95% B; t = 24 min, 5% B; t = 25 min stop, at a flow rate of 0.3 mL/min.

LC-HRMS analysis of CDA metabolism. From a 2 mg/mL Micelle@GEM, a solution with a final concentration of 50μM of TOS-GEM was incubated in PBS buffer to which 1 μL of CDA (0.5 mg/mL) was added. The reaction was quenched with ACN, centrifuged and the supernatant analyzed by LC- HRMS in Positive Mode. Similar assays with 50μM GEM + 1 μL of CDA and PBS + 1 μL of CDA were performed. The HPLC runs were carried out with a

gradient of A (Milli Q water containing 0.1% v/v Formic acid, FA) and B (acetonitrile containing 0.1% v/v FA, Honeywell HPLC-grade). The mobile phase was t = 0 min, 5% B; t = 5 min, 5% B; t = 18 min, 95% B; t = 22 min, 95% B; t = 24 min, 5% B; t = 25 min stop, at a flow rate of 0.3 mL/min. Quantification was performed based on the EIC intensity (AUC) within the δ 6ppm range. **Gemcitabine**: RT 1.7 min; **GEM metabolite**: RT: 2.49 min; **TOS-GEM**: RT: 22.10 min, detection by TIC.

UV-Vis quantification of cargo-loaded micelles. A dialysis diffusion method was used to quantify the internal cargo release kinetics. Rhodamine-loaded micelle@GEM in a 1:30 w/w ratio (5 mL solution, 2 mg micelle per mL) was loaded into dialysis bags (3KDa cut-off) and dialysed against PBS buffer (250 mL, pH 7.4, 37°C) under continuous gentle stirring. Aliquots were taken at 0h, 24h, and 120h both from inside micelle@GEM/Rho solution (50 µL) and PBS buffer (3 mL), and Rho absorption was monitored and quantified by UV-Vis. A similar procedure was performed for micelle@PTX, which was quantified using LC-HRMS.

Quantification of cargo loading. PTX and Rho concentration in the supernatant after micelle formulation was quantified using LC-HRMS.

LC-HRMS analysis of drug release hydrogel formulations.

Method A: Using dialysis diffusion method, 0.5mL of hyd + micelle@GEM; hyd + micelle@PTX; hyd + micelle@PTX + GEM or hyd + free GEM + PTX was taken for dialysis using a MWCO 3.5 kDa Pur-A-Lyzer™ Maxi Dialysis Kit and dialyzed for 24h, 48h and 72h against PBS (pH 7.4, 37°C). The progression of each dialysis was followed by LC- HRMS in Positive Mode. The HPLC runs were carried out with a gradient of A (Milli Q water containing 0.1% v/v Formic acid, FA) and B (acetonitrile containing 0.1% v/v FA, Honeywell HPLC-grade). The mobile phase was t = 0 min, 5% B; t = 5 min, 5% B; t = 18 min, 95% B; t = 22 min, 95% B; t = 24 min, 5% B; t = 25 min stop, at a flow rate of 0.3 mL/min.

Method B: Transwell cell culture inserts with a 0.4-mm pore membrane (Corning, USA) were used to study the release profile from the hydrogel. Hyd + micelle@GEM; hyd + micelle@PTX; hyd + micelle@PTX + GEM or hyd + free GEM + PTX (0.5mL each) were deposited in the upper part of the Transwell membrane, 0.5mL of Milli-Q water was added to the well, and fresh PBS (pH 7.4, 37°C) was added to the well. At different time points, supernatants were collected and monitored by LC- HRMS in Positive Mode. The HPLC runs

were carried out with a gradient of A (Milli Q water containing 0.1% v/v Formic acid, FA) and B (acetonitrile containing 0.1% v/v FA, Honeywell HPLC-grade). The mobile phase was t = 0 min, 5% B; t = 5 min, 5% B; t = 18 min, 95% B; t = 22 min, 95% B; t = 24 min, 5% B; t = 25 min stop, at a flow rate of 0.3 mL/min.

Rheological behaviour of hyaluronic acid hydrogel. Rheological characterisation of HA mixtures was performed using a Modular Compact Rheometer MCR 502 (Anton Paar, Madrid, Spain), equipped with an electric plate for temperature control (± 0.01 ˚C). A cone-plate geometry with a diameter of 50 mm and 1º was used. Steady-shear flow measurements were performed to characterise the viscosity flow curve in the shear-rate range of 0.01 to 100 $1.s^{-1}$. Oscillatory shear measurements were performed to evaluate the storage G' and loss G" moduli within the linear viscoelastic regime (LVR) to ensure that the moduli are strain-independent. The amplitude strain sweep was performed at 1 Hz in the range of 0.001 to 1%, and the angular frequency sweep measurements were carried out under a constant strain of 1% in the frequency range of 0.1 to 10 Hz. All measurements were carried out at 25 °C on fresh samples. The experiments were performed in triplicate.

Hyaluronic acid hydrogel microstructure. After casting into the molds, hydrogel or hydrogel containing PTX, GEM or both micelles were frozen at −80 °C and subsequently freeze-dried. Moreover, the samples were freeze-fractured after immersion in liquid nitrogen to expose their inner structures and sputter-coated (10 nm, Quorum) with iridium prior to observation under a scanning electron microscope (Hitachi, Japan).

Dosing rationale. Treatments were administered at a fixed total depot/polymer load and constant injection volume across groups. For the combination, the GEM:PTX ratio was prespecified from in-vitro potency (IC_{50} for GEM; IC_{10} for PTX) and applied to the nanoparticle payload; thus, the per-drug dose (mg/kg) in the combination is lower than in the corresponding monotherapies. Exact target doses for each group and the fixed ratio are reported in Table S1 and the relevant figure legends. IC50 (µM): GEM=0.035, micelle@GEM=0.015, PTX=0.185, and micelle@PTX=0.003. IC10 (µM): GEM=0.01, micelle@GEM=0.0035, PTX=0.04, and micelle@PTX=0.0007, see Table S3.

Table S1. Dosing Rationale of the *in vivo* PDAC treatment

	Cytotoxic drug (mg) / kg of the animal	TOS-SSOH/ kg of the animal	TOS-GEM/ kg of the animal	TPGS/ kg of the animal	Micelle/ kg of the animal
Micelle@GEM	2.23 mg covalently bonded into TOS-GEM	0	6.73 mg	4.5 mg	11.23 mg
Micelle@PTX	0.73 mg	13 mg	0	8.56 mg	21.65 mg
Free PTX+GEM	2.23 mg (GEM) and 0.73 mg (PTX)	Not apply	Not apply	Not apply	Not apply

3. Results and Discussion

The progression of each reaction solution was followed by LC- HRMS in Positive Mode. Release of GEM and PTX were measured by quantification of their EIC chromatograms (within δ 6 ppm range; Figs S13 and S15), allowing to stablish GSH-dependent GEM release, of GEM from CDA-mediated degradation, PTX diffusion profile from micelle@PTX (for methods see 2.

Micelles characterisation

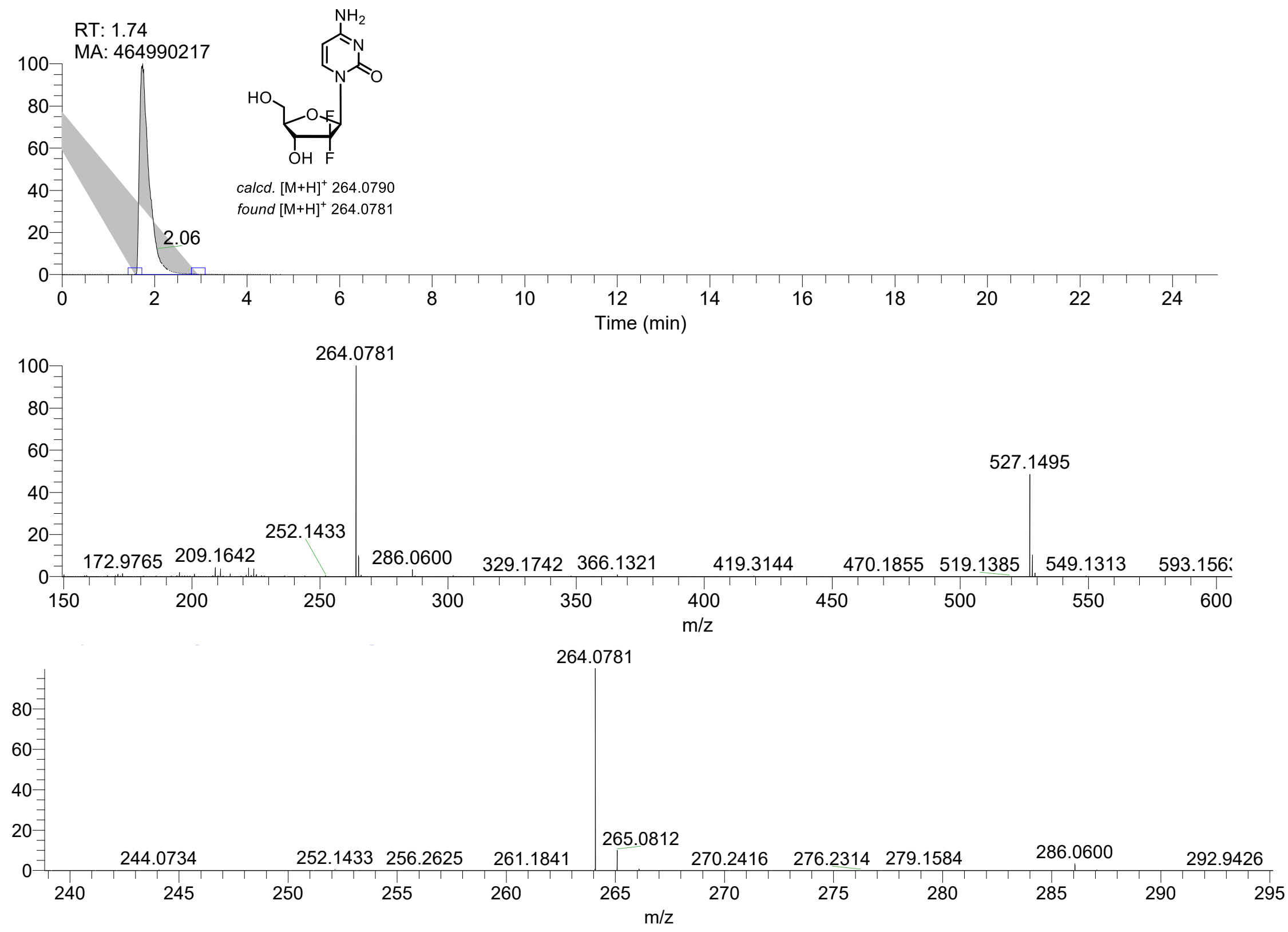

Fig. S13. LC-HRMS analysis of gemcitabine.

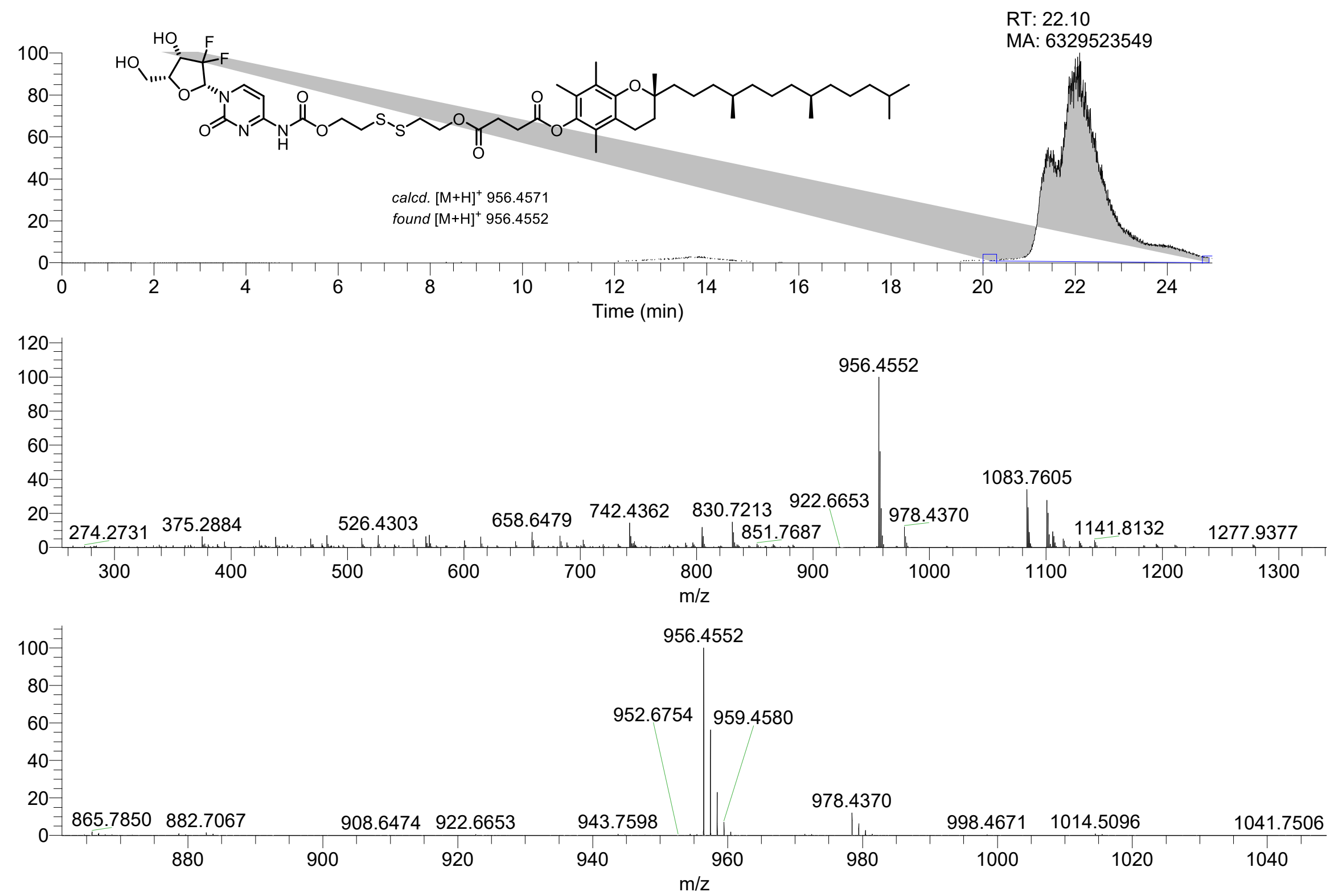

Fig. S14. LC-HRMS analysis of TOS-GEM.

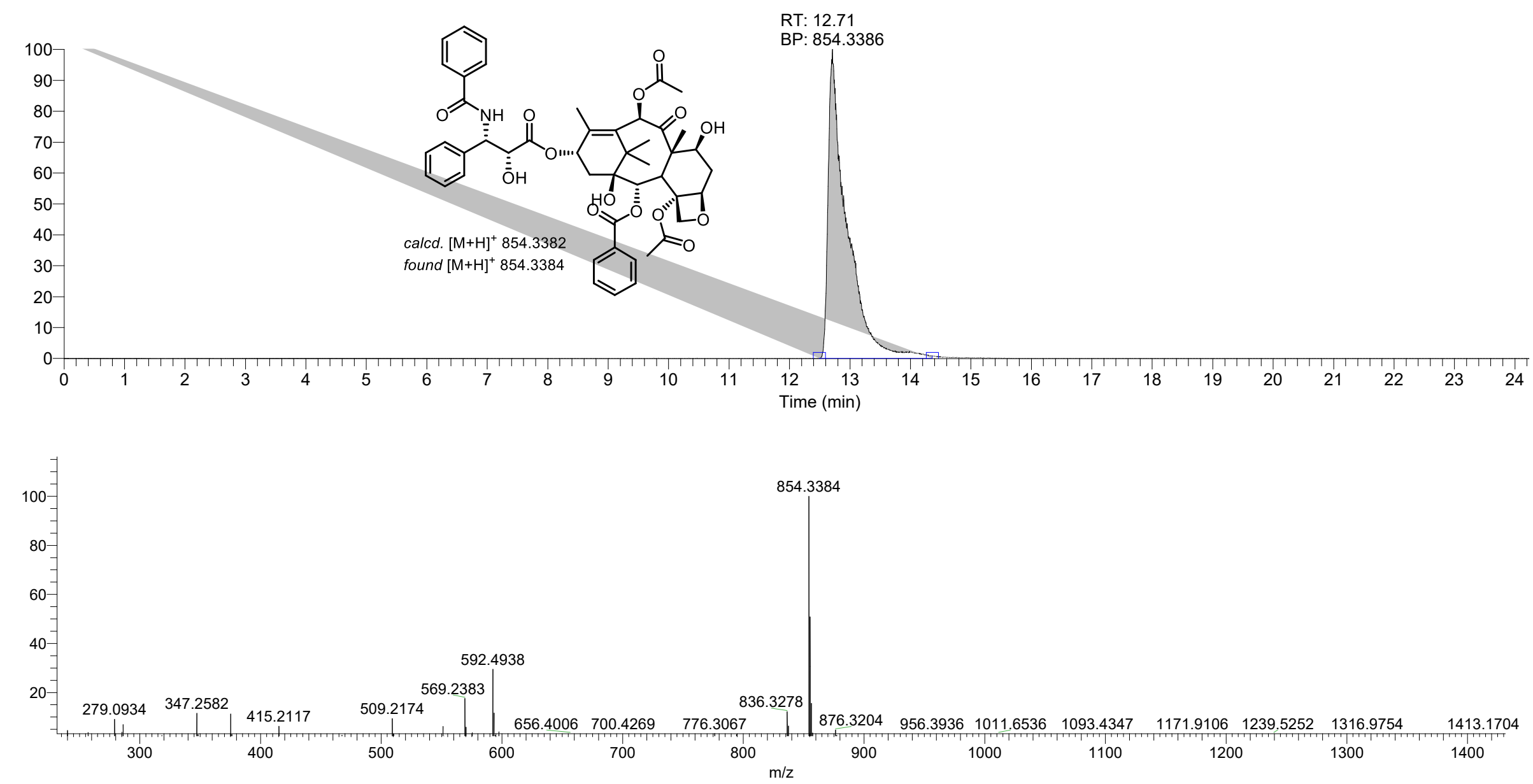

Fig. S15. LC-HRMS analysis of Paclitaxel.

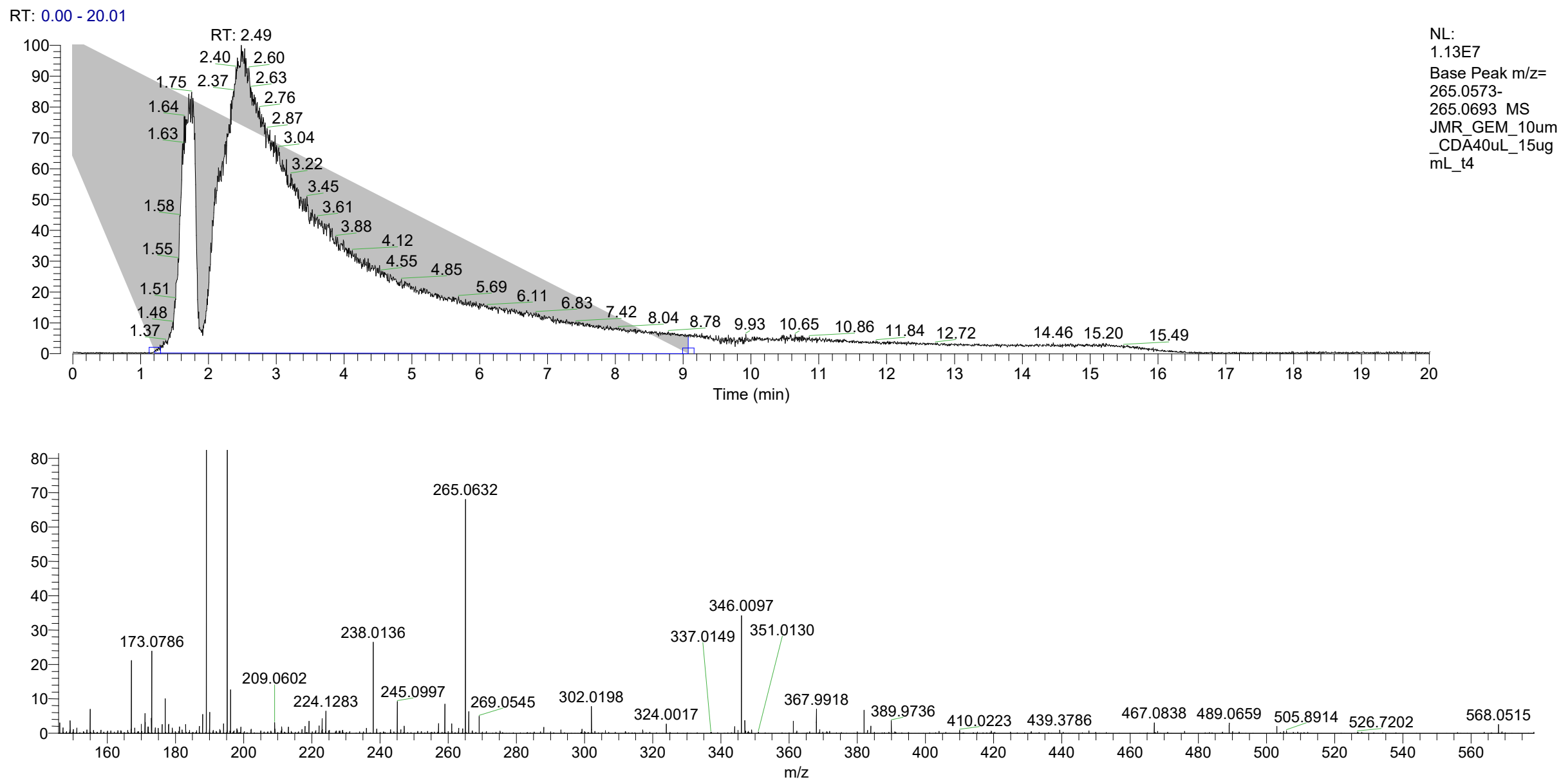

Fig. S16. LC-HRMS analysis of GEM CDA metabolite.

Formulation optimization was performed by varying the TPGS/TOS-SS-GEM ratios under self-assembly conditions until achieving the optimal ratio which allowed the highest incorporation of TOS-SSOH (TOS-SSOH, 40%; TPGS, 60%; Fig. S17). Formulations of micelle@GEM and micelle@PTX followed this ratio according to the procedure described in

section 2 and tolerated high levels of TOS-SSOH exchange with TOS-SS-GEM and/or cargo encapsulation. Spherical micelles 120±20 nm in size with low polydispersity were obtained at the optimized ratio (Fig. S18) and with good encapsulation efficiency and cargo loading (Table S2). Micelles also suffered little hydrodynamic post drug release (Fig. S19).

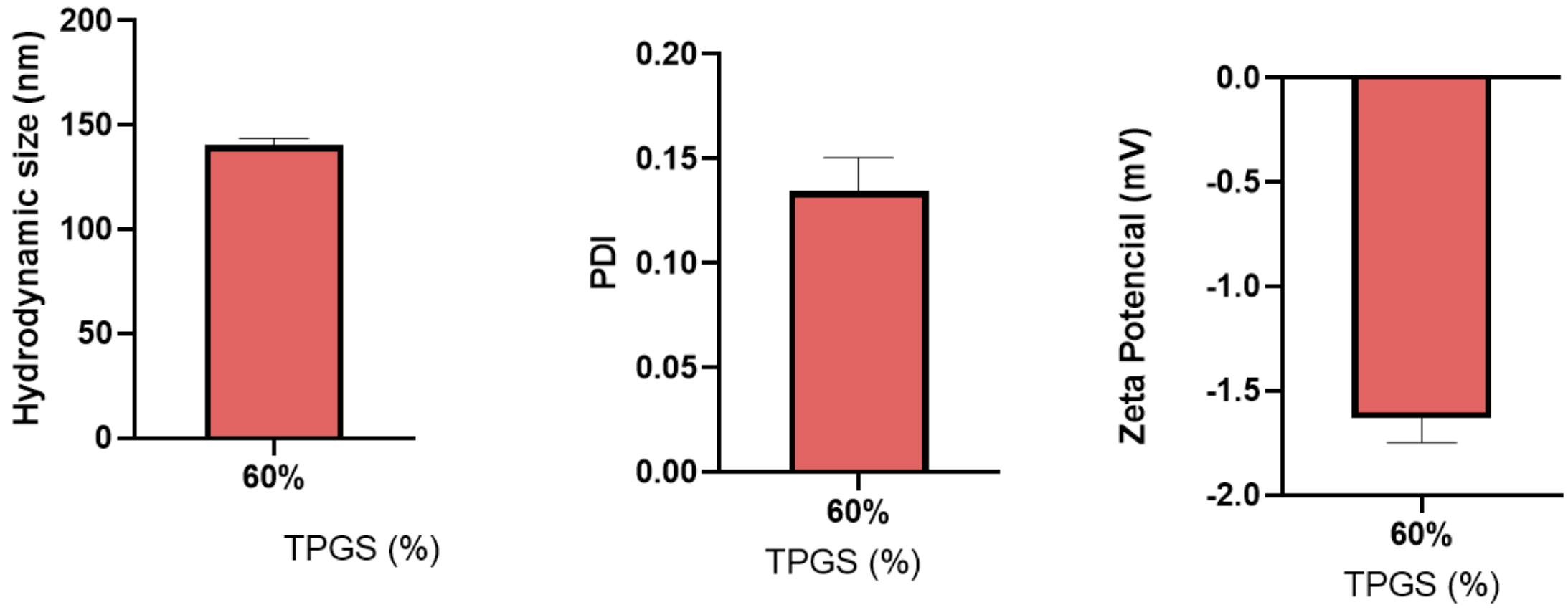

Fig. S17. Hydrodynamic size by DLS, polydispersity index, and iii) and charge based on zeta potential of naked micelles (TOS-SSOH, 40%; TPGS, 60%). N = 3 independent experiments, mean ± SD

a) micelle@GEM **B) micelle@PTX** **c) naked micelles**

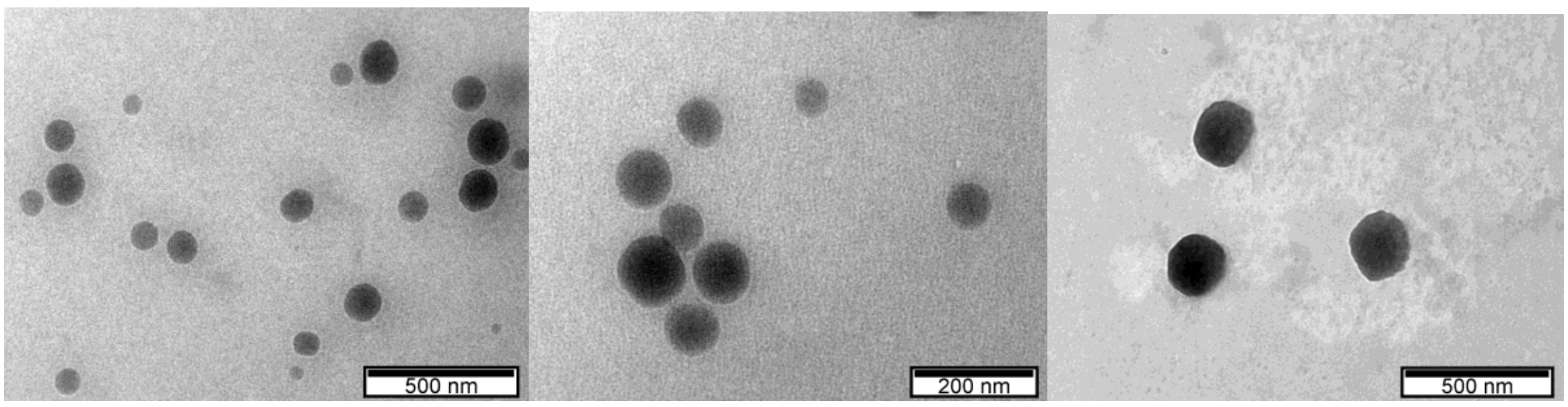

Fig. S18. a-c) TEM images of micelle@GEM, micelle@PTX and naked micelles, respectively.

Table S2. Cargo loading and encapsulation efficiency, as measured by LC-HRMS

	**Encapsulation efficiency (%)**	**Cargo loading (w/w;%)**
**PTX**	94.2%	**30.4%**
**Rho**	93.3%	**30.1%**

The calculation of encapsulation efficiency, cargo loading (%w/w), and DL (mg/mg) was based on the following formulas:

$$Encapsulation\ efficiency = \frac{Amount\ of\ cargo\ loaded\ (mg)}{Amount\ of\ cargo\ added\ (mg)}$$

$$Cargo\ Loading = \frac{Amount\ of\ cargo\ loaded\ (mg)}{Amount\ of\ micelle\ constituents\ (mg) + amount\ of\ cargo\ loaded\ (mg)} \times 100$$

Micelle characterization post hydrogel release

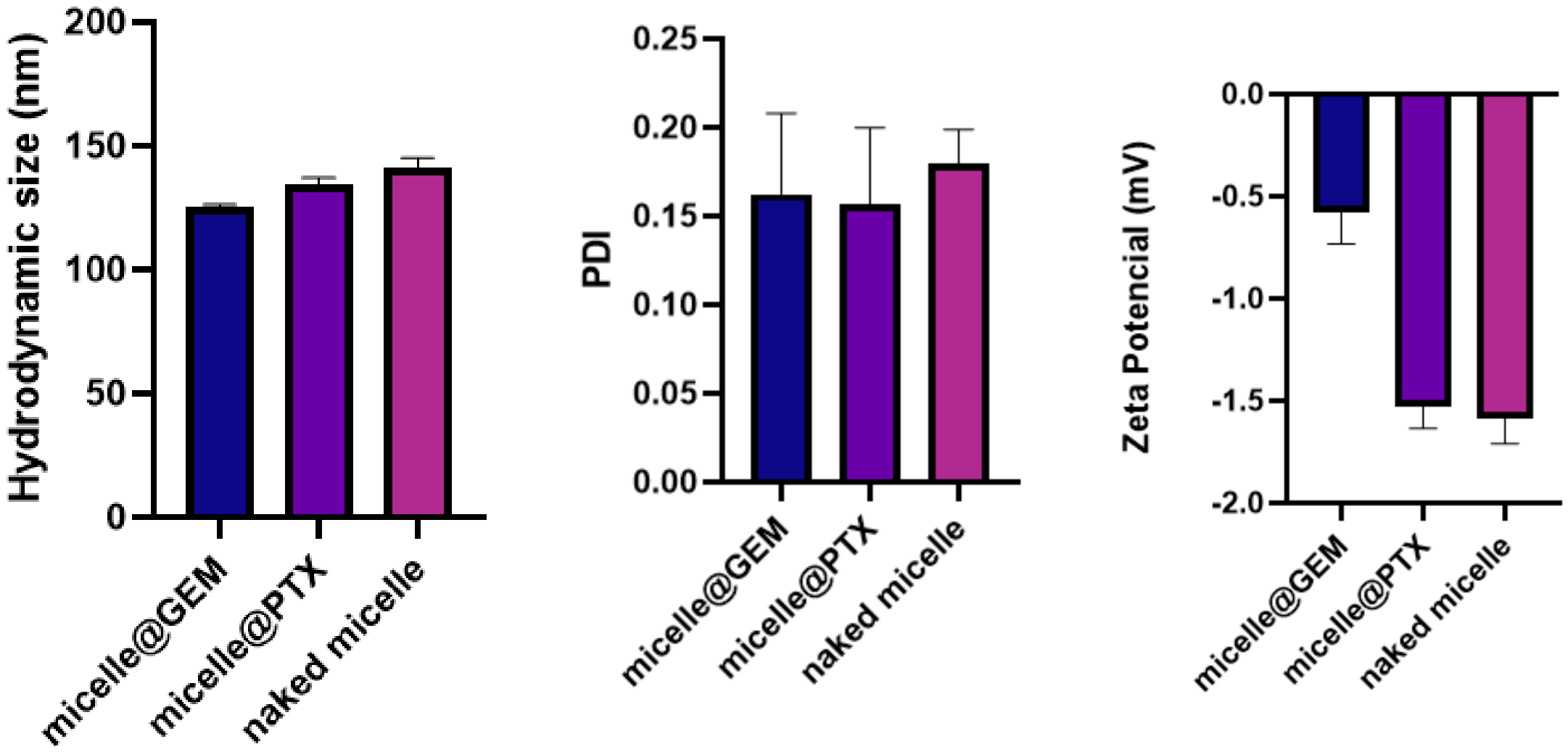

Fig. S19. Hydrodynamic size by DLS, polydispersity index, and charge based on zeta potential of micelles after 24h transwell release assay (method B) from hyd + micelles. N = 3 independent experiments, mean ± SD.

***In vitro* cell studies**

Table S3. In vitro cell viability assays. IC50 (µM) of pancreatic cell line treated with different compounds for 72 h. (Mean).

Drug/Micelles	BxPC-3 (IC_{50})
GEM	0.035
Micelle@GEM	0.015
PTX	0.185
Micelle@PTX	0.003

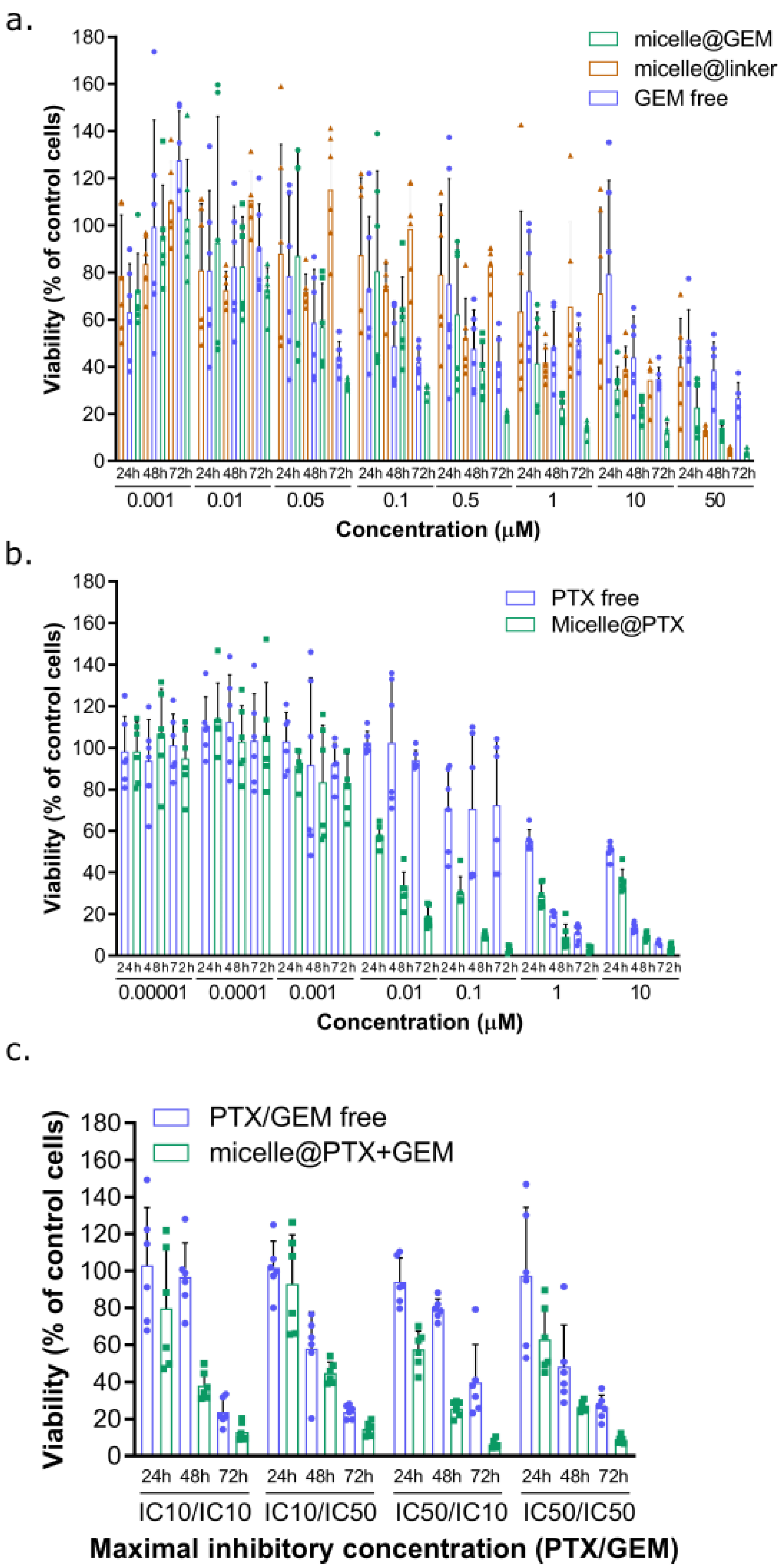

Fig. S20. In vitro cell viability assays. a-c) Bar graph illustrating the enhanced metabolic activity of micelle-encapsulated PTX and GEM (micelle@PTX/micelle@GEM) compared with free drugs in pancreatic cancer cells, with significant reductions in cell metabolic activity at various drug concentrations. N = 3 independent biological experiments.

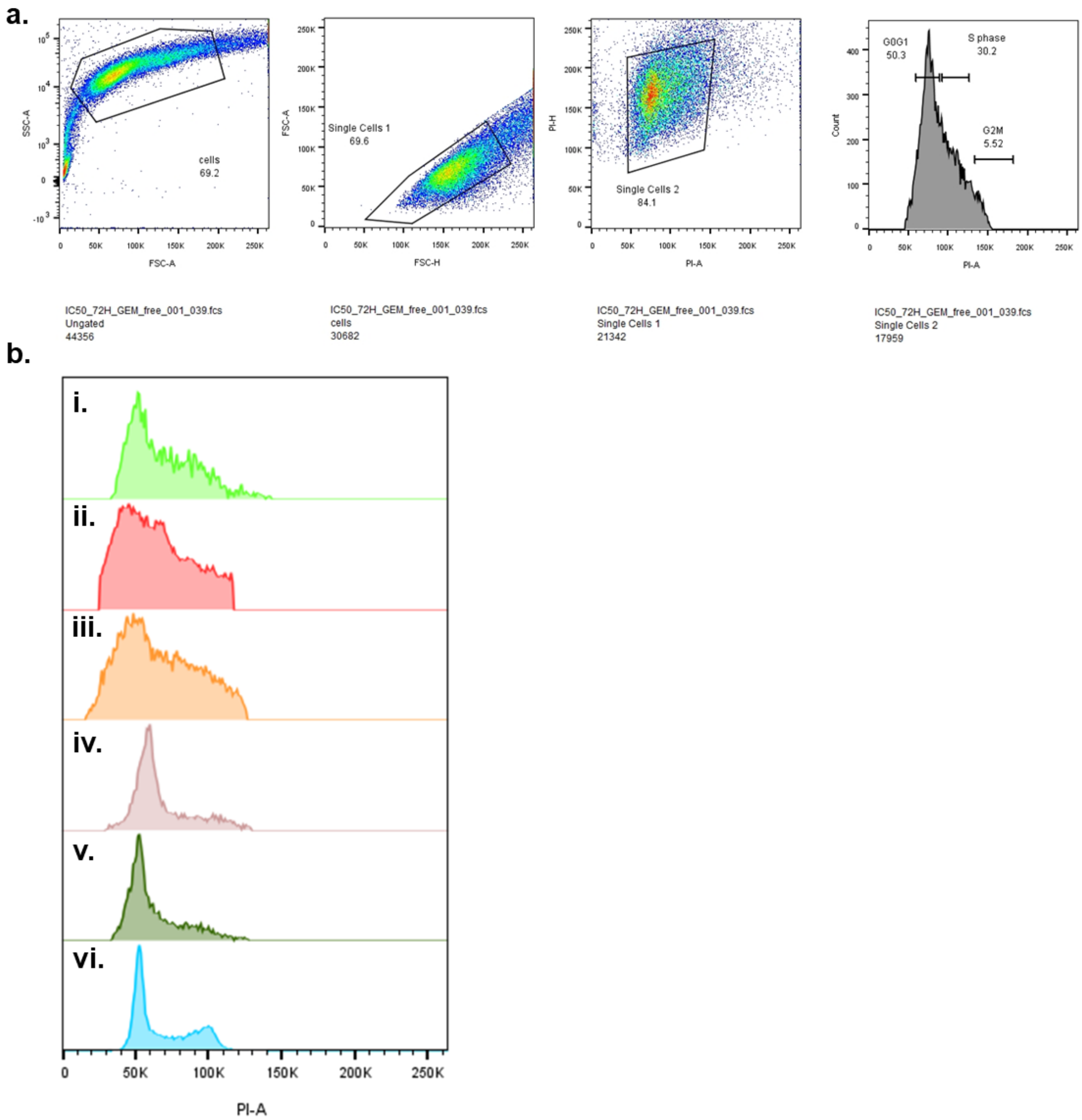

Fig. S21. Cell cycle evaluation. A. Gating strategy applied to control and treated samples. Debris and doublets were discarded. PI was used to identify the phases according to the mean fluorescence intensities. **B.** Histograms of the cell cycle distribution of pancreatic cancer cells after treatment with **i)** micelle@PTX/micelle@GEM, **ii)** micelle@PTX, **iii)** free PTX, **iv)** micele@GEM, **v)** free GEM, and **vi)** linker-modified micelles (micelle@linker), indicating possible G1 phase cell cycle arrest by micelle@GEM. N = 3 independent experiments.

Hyaluronic acid hydrogel characterization

The observed shear-thinning behaviour **(Fig. S22)** supports the intended route of administration by allowing injection through a narrow-gauge needle while reducing viscosity under shear. After injection, recovery of the viscoelastic network is expected to favour local depot formation and prolonged retention in the peritoneal cavity **(Fig. S23)**. This interpretation is consistent with the established design principles of injectable HA hydrogels and with our biodistribution data, which show prolonged abdominal retention of the micelle-loaded depot.

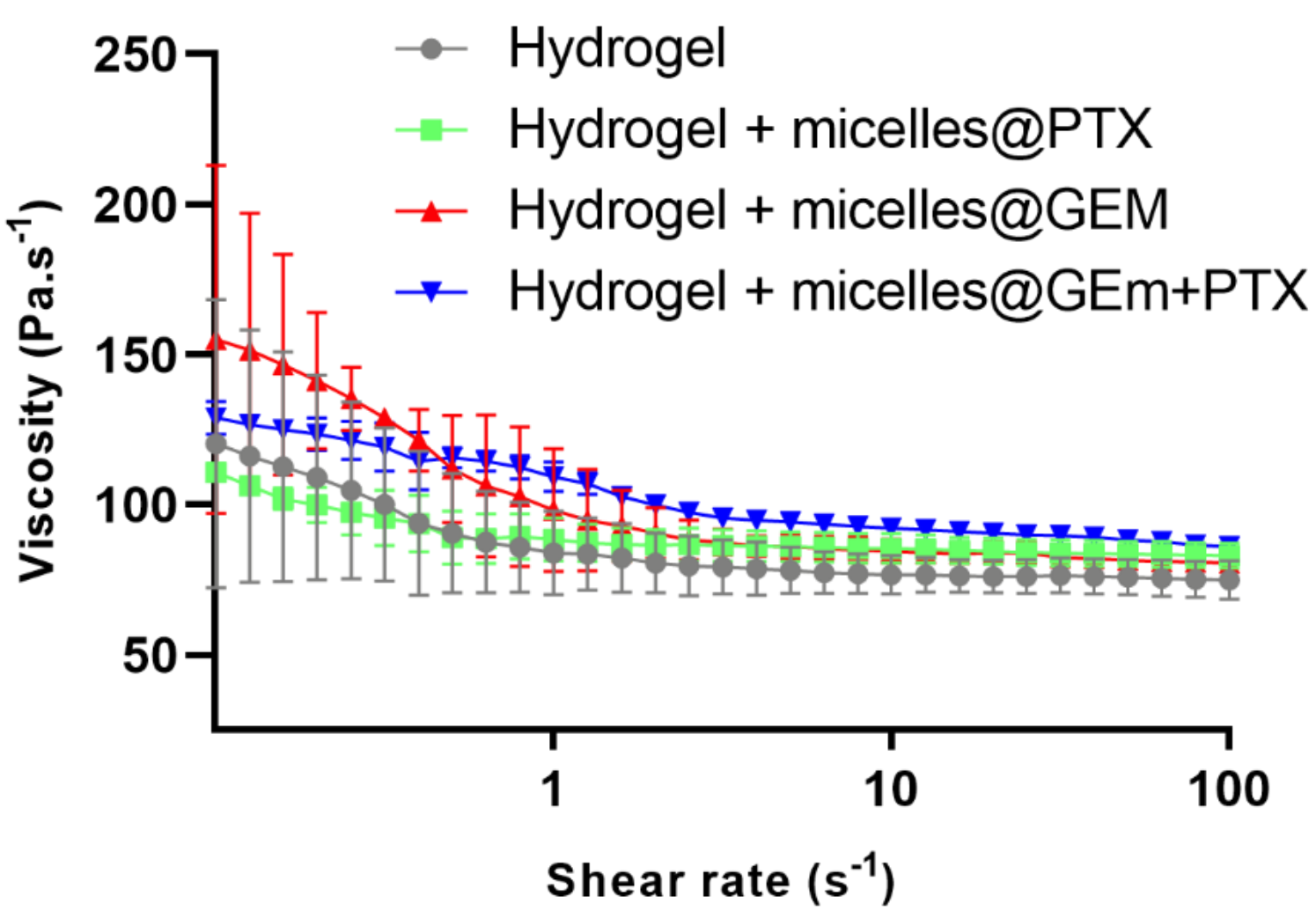

Fig. S22. Rheological properties of hydrogel formulations. Viscosity of hydrogel formulations as a function of shear rate.

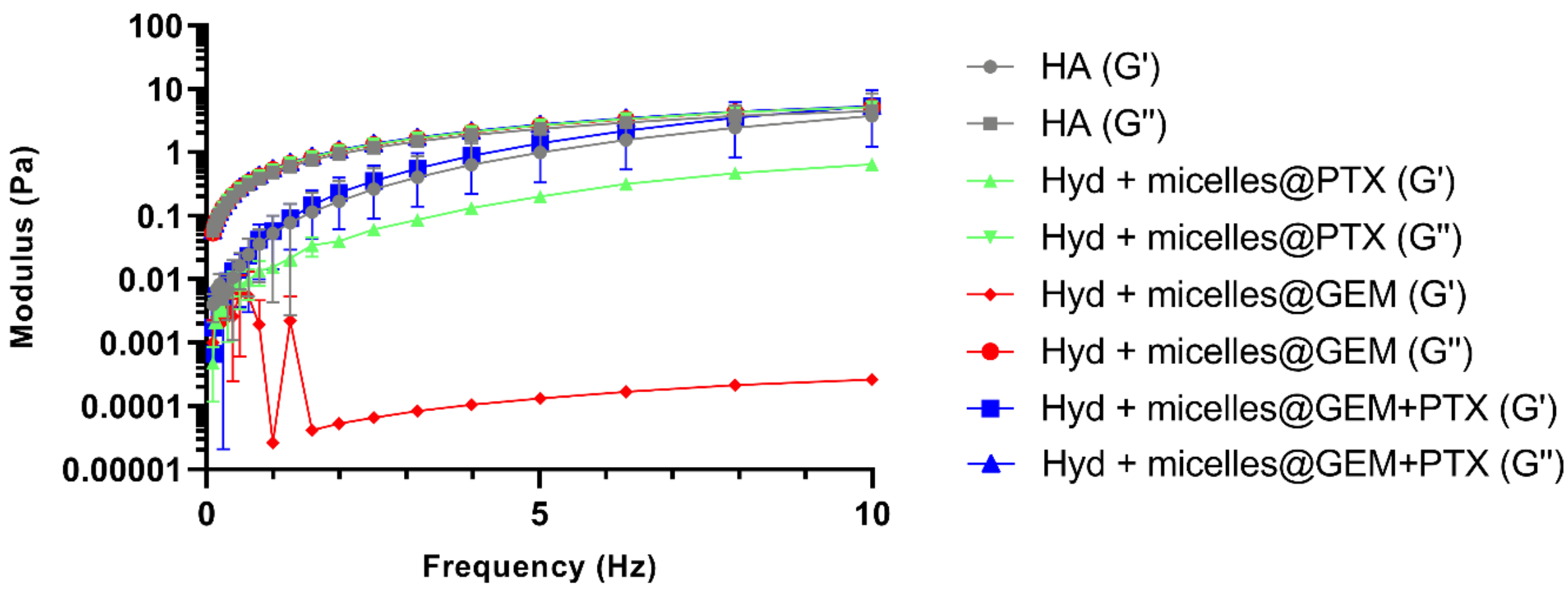

Figure S23. Rheological properties of hydrogel formulations. Viscoelastic properties at 1 Hz.

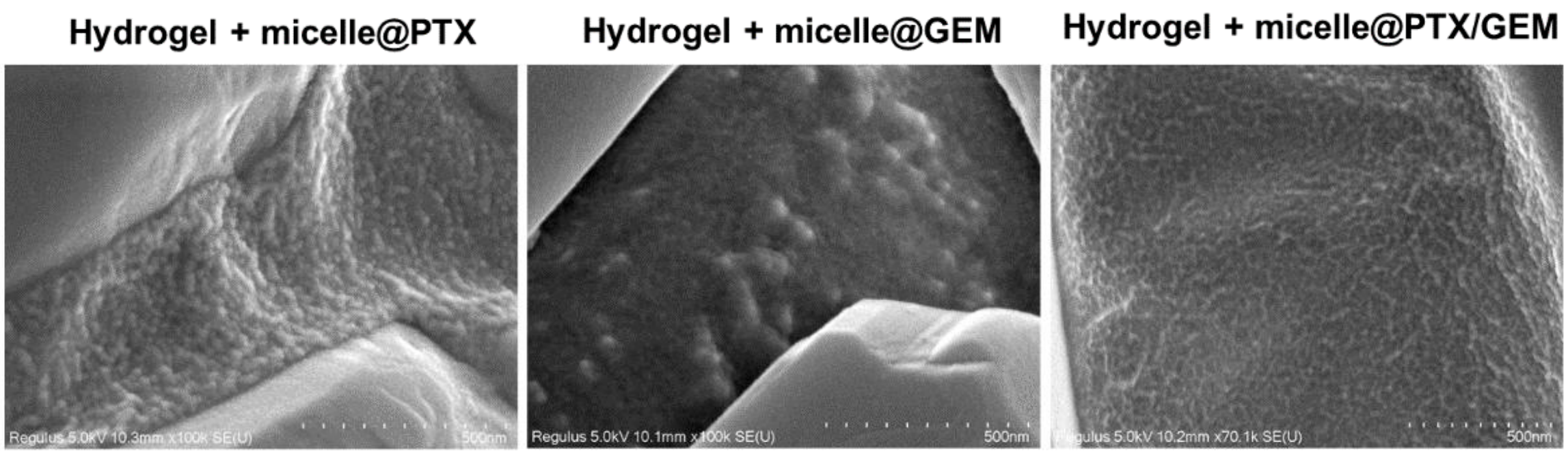

Fig. S24. Scanning Electron Microscopy (SEM) images revealed the morphological characteristics of hyaluronic acid-based hydrogels. The pannel shows hydrogels loaded with micelles containing paclitaxel (PTX), gemcitabine (GEM), or a combination of both (PTX/GEM). The images reveal the changes in surface topology and porosity upon micelle encapsulation, illustrating a homogeneous micelle distribution within the hydrogel matrix. Scale bar: 10 µm (top and middle panels) and 500 nm (bottom panel).

***In vivo* study**

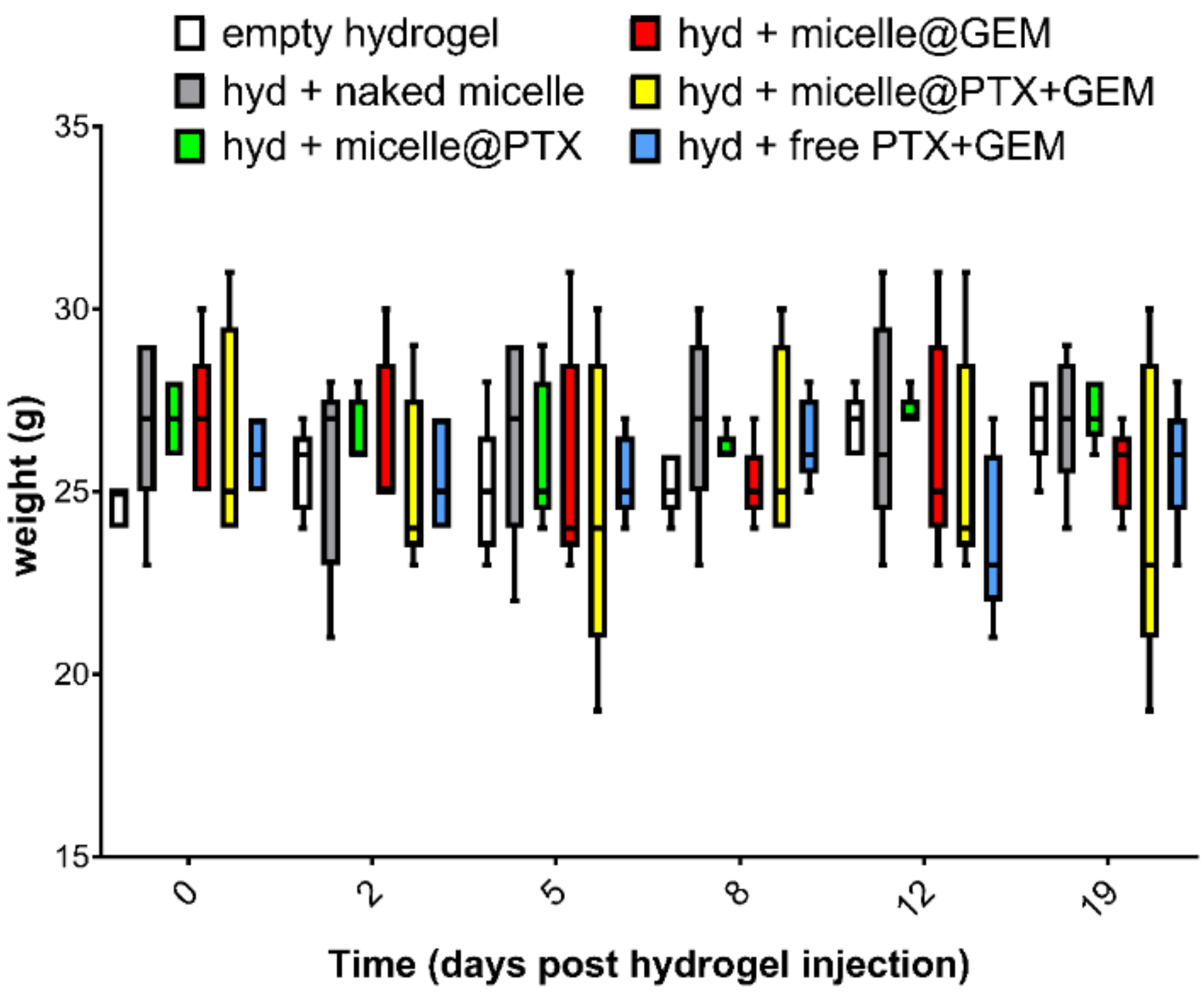

Fig S25. **Body weight monitoring of mice subjected to different PDAC treatments over time.** Boxplot representing body weight changes in mice over 19 days following the injection of various formulations: empty hydrogel, hyd-conjugated with naked micelles, hyd-conjugated micelles with paclitaxel (PTX), hyd-conjugated micelles with gemcitabine (GEM), a combination of hyd-conjugated micelles with PTX and GEM, and free combination of PTX and GEM.

Table S4. Humane Endpoint Criteria Applied to Individual Animals Throughout the Study.

Animal ID	Group	Humane Endpoint	Days
1	empty hydrogel	BLI ROI	2
2		BLI ROI	8
3		BLI ROI + Abdominal distension	19
4		Abdominal distension	19
5		Abdominal distension	19
6	hyd + naked micelle	BLI ROI	12
7		Abdominal distension	19
8		BLI ROI + Abdominal distension	19
9		Abdominal distension	19
10		BLI ROI	5
11	hyd + micelle@PTX	N/A	No
12		N/A	No
13		N/A	No
14		N/A	No
15		N/A	No
16	hyd + micelle@GEM	N/A	No
17		N/A	No
18		N/A	No
19		N/A	No
20		N/A	No
21	hyd + micelle@PTX+GEM	N/A	No
22		N/A	No
23		N/A	No
24		N/A	No
25		N/A	No
26	hyd + free PTX+GEM	BLI ROI	12
27		BLI ROI + Abdominal distension	19

28		Abdominal distension	19
29		Abdominal distension	19
30		Abdominal distension	19

Abbreviations: BLI ROI: bioluminescence region of interest.

Table S5. Number of animals at risk table in Kaplan-Meier survival analysis.

Time (days post hyd inject)	empty hydrogel	hyd + naked micelle	hyd + micelle@PTX	hyd + micelle@GEM	hyd + micelle@PTX+GEM	hyd + free PTX+GEM
0	5	5	5	5	5	5
2	5	5				
5		4				
8	4					
12						5
19	3	3	5	5	5	4
Median Survival	19	19	Undefined	Undefined	Undefined	19

Intratumoral and metastatic genetic profiling

The transcriptomic analysis begins with the validation of data structure and sample comparability. **Supplementary Fig. S26** shows the Pearson correlation matrix across all experimental samples, providing an essential quality-control framework for the downstream RNA-seq interpretation. The presence of strong within-dataset correlations supports the consistency of the sequencing data, while the detectable differences between sample groups indicate that the transcriptomic variation is biologically informative rather than dominated by technical noise. This is important because the study relies on comparing primary tumours and metastatic lesions after hydrogel-based delivery of micelle@GEM, micelle@PTX and micelle@PTX+GEM. Therefore, **S26** establishes that the subsequent gene-expression and pathway analyses are built on a coherent dataset capable of resolving treatment- and site-specific molecular responses. This is further supported by **Supplementary Figs. S27 and S28**, which show the distribution of log-transformed FPKM values and log2-transformed gene-expression levels across the different groups. The broadly comparable distribution profiles indicate that the sequencing libraries and expression-normalisation strategy are suitable for comparative analysis. At the same time, the observed spread in expression values captures the expected biological heterogeneity of advanced pancreatic cancer, especially when primary peritoneal tumour deposits are compared with metastatic lesions in the pancreas, liver and spleen. Together, **S26 to S28** provide the technical foundation for interpreting the RNA-seq results as true pharmacogenomic responses to the micelle-hydrogel formulations rather than artefacts of uneven sequencing depth or poor sample quality.

After establishing dataset robustness, **Supplementary Fig. S29** introduces the global transcriptional architecture of the treated tumours. The PCA plot shows that the first two components explain a substantial fraction of the observed variation, with PC1 accounting for 36.04% and PC2 for 8.63%. This separation indicates that treatment condition and anatomical

tumour site impose measurable transcriptional structure. This is particularly relevant for this study because pancreatic cancer metastases are not molecularly identical to primary tumour deposits; each metastatic site is shaped by a distinct microenvironment, vascular exposure, stromal composition and drug-accessibility profile. Thus, **S29** supports the central concept that intraperitoneal hydrogel-micelle delivery generates spatially heterogeneous molecular responses, with primary tumours and organ-specific metastases responding differently to GEM, PTX or their combination.

This global separation is then expanded in **Supplementary Figs. S30 and S31**, which show heatmap-based clustering of differentially expressed genes across treatment conditions, gene categories and chromosomal locations. These figures are important because they show that the response to micelle-carried chemotherapy is not limited to isolated marker genes but involves coordinated transcriptional programmes. The presence of red and green expression blocks across different sample groups indicates that the formulations reshape gene networks in a treatment-dependent and site-dependent manner. This supports the main manuscript's interpretation that the hydrogel-micelle platform does more than improve drug exposure; it also modulates oncogenic and stress-adaptation pathways, including PI3K-Akt, MAPK, VEGF-related signalling, ubiquitination, proteasome degradation and antigen-processing pathways. In this context, **S30 and S31** provide the bridge between the phenotypic efficacy data, reduced tumour burden and metastatic spread, and the molecular mechanism of action proposed for the micelle-based delivery system.

Supplementary Fig. S32 gives quantitative weight to this interpretation by showing the number of differentially expressed genes across the different treatment comparisons. The figure reveals a strong organ- and formulation-dependent DEG burden. In primary tumours, hyd + micelle@GEM and hyd + micelle@PTX+GEM induced a stronger transcriptional response than hyd + micelle@PTX, consistent with the prominent antitumour activity of GEM-containing micelles in the primary tumour compartment. In pancreatic metastases, the number of DEGs increased markedly, particularly after hyd + micelle@PTX and hyd + micelle@PTX+GEM, indicating that metastatic pancreatic lesions may be especially sensitive to micelle-mediated transcriptional remodelling. In liver and spleen metastases, hyd + micelle@PTX induced particularly large DEG changes, while hyd + micelle@GEM and the combination also produced substantial but distinct profiles. This is important because it shows that each organ niche has a specific pharmacogenomic response. The therapeutic effect of the platform should therefore be interpreted not as a uniform drug effect, but as a compartmentalised response shaped by the interaction between formulation, payload-release kinetics and metastatic microenvironment. This strengthens the rationale for using local hydrogel depots and nanoscale drug carriers to modulate metastatic pancreatic cancer at multiple anatomical sites.

The final thematic block, **Supplementary Figs. S33 to S35**, connects the mouse treatment-response data with human pancreatic cancer biology. **Supplementary Fig. S33** uses TCGA-derived oncoprint analysis to show genetic alterations in ubiquitination and proteasome pathway genes, including RNF123, PSMA1, TRAF7, BTRC, NEDD4L, NEDD4, CUL2 and SKP2. This is important because the same biological axis emerges from the treatment-response analysis, particularly in relation to ubiquitination, proteasome degradation and MHC class I antigen processing. The presence of somatic mutations and copy-number alterations in these genes in human pancreatic cancer indicates that the pathways modulated by micelle-delivered therapy are not incidental experimental readouts, but clinically relevant components of PDAC

biology. **S33** therefore strengthens the translational relevance of the study by showing that the molecular programmes affected by the nanotherapy overlap with pathways altered in patient tumours.

Supplementary Fig. S34 then moves from genomic alteration to expression during disease progression. The figure shows that ubiquitination and proteasome-related gene signatures are increased in tumour and metastatic tissues compared with normal tissue. The ridge and radar plots further highlight pronounced expression shifts for genes such as PSMA1 and PSMB2, suggesting enhanced proteasome activity and protein-turnover dependence during pancreatic cancer progression. This is mechanistically relevant because aggressive pancreatic cancer cells experience high proteotoxic, metabolic and therapeutic stress. Increased dependence on protein degradation machinery may help cancer cells maintain survival, remove damaged proteins, adapt to stress and regulate immune visibility through antigen processing. Therefore, **S34** supports the interpretation that micelle-based GEM delivery affects a biologically vulnerable axis in PDAC: proteostasis coupled to immune antigen processing. This provides a plausible mechanistic link between improved intracellular drug delivery, transcriptional remodelling and reduced tumour progression.

Supplementary Fig. S35 provides the strongest clinical prioritisation of candidate genes emerging from this axis. The survival and expression analyses of PSMA1, SKP2 and UBE2C show that these genes are upregulated in pancreatic tumour and metastatic contexts, but their clinical implications differ. PSMA1 and SKP2 appear mainly as progression-associated proteostasis markers, with increased expression in cancer tissues relative to normal tissues. UBE2C shows the clearest association with aggressive disease, with high expression linked to poorer overall survival and higher expression in advanced-stage disease. This distinction is important for the manuscript because it avoids overclaiming that all genes have equivalent prognostic value. Instead, the data show that PSMA1, SKP2 and related proteasome/ubiquitination genes define a clinically relevant stress-adaptation programme, while UBE2C emerges as the strongest prognostic candidate within this set. In combination with **S33** and **S34, S35** therefore reinforces the idea that the therapeutic benefit of the hydrogel-micelle system is associated with modulation of clinically meaningful pathways involved in protein degradation, tumour progression and metastatic adaptation.

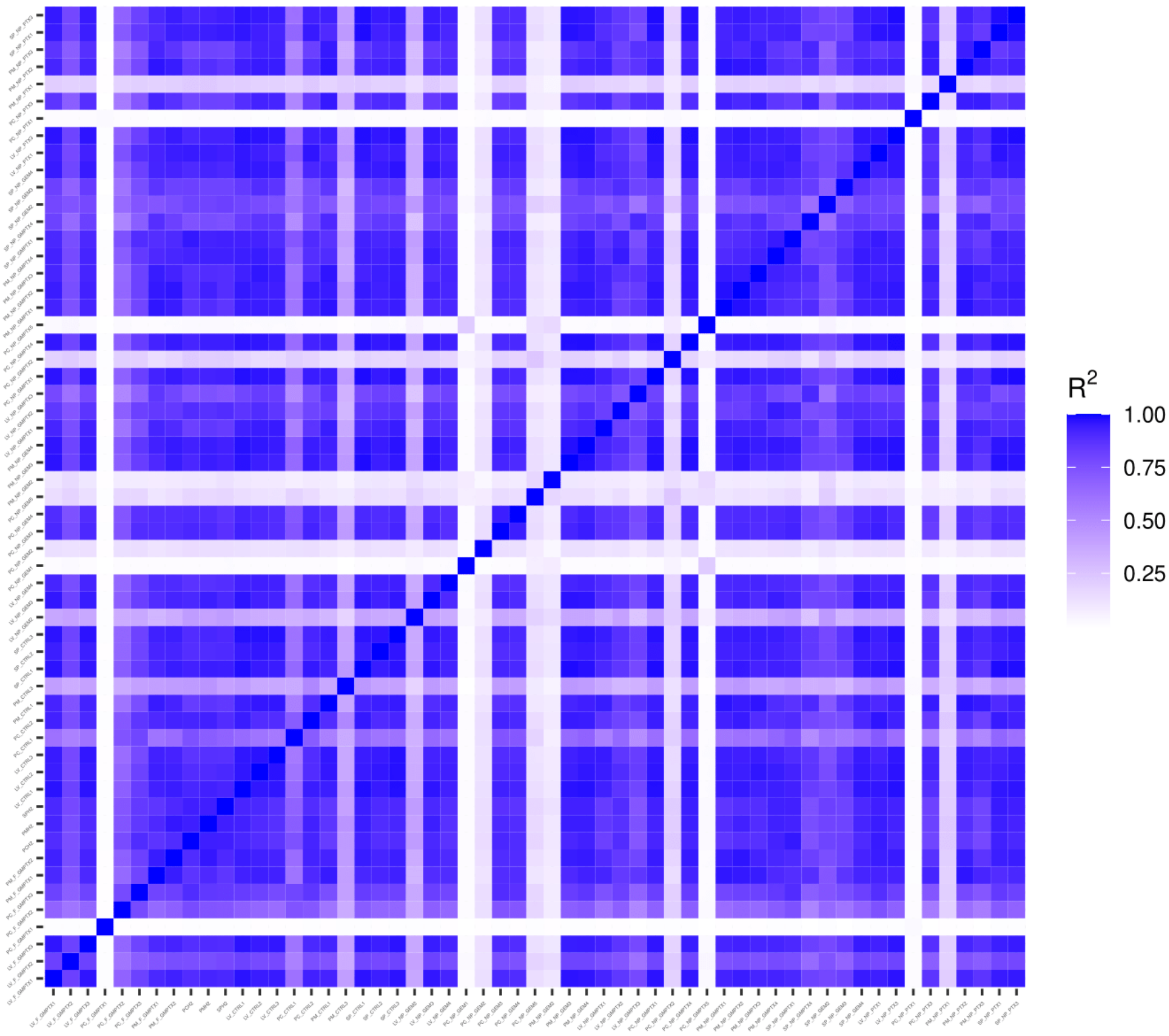

Fig S26. Correlation matrix displaying Pearson correlation coefficients between all experimental samples, with intensity of blue indicating strength of correlation, ranging from R^2 = 0.25 (lightest) to R^2 = 1.00 (darkest).

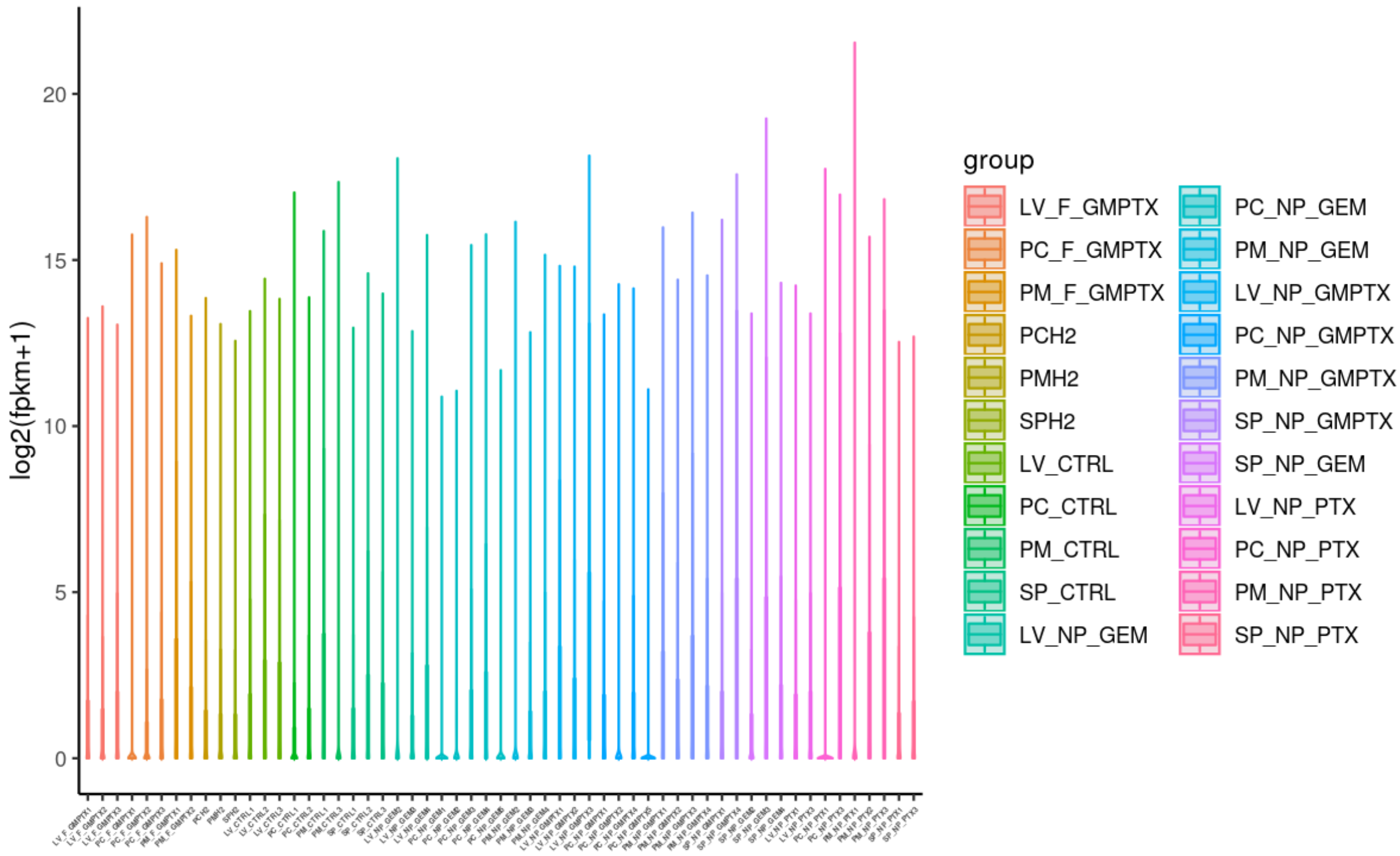

Fig S27. Violin plots displaying the distribution of log-transformed FPKM values across all sample groups indicate the variability and density of gene expression levels within each group. Each colour represents a different treatment or control group, allowing for a comparative overview of the expression profiles.

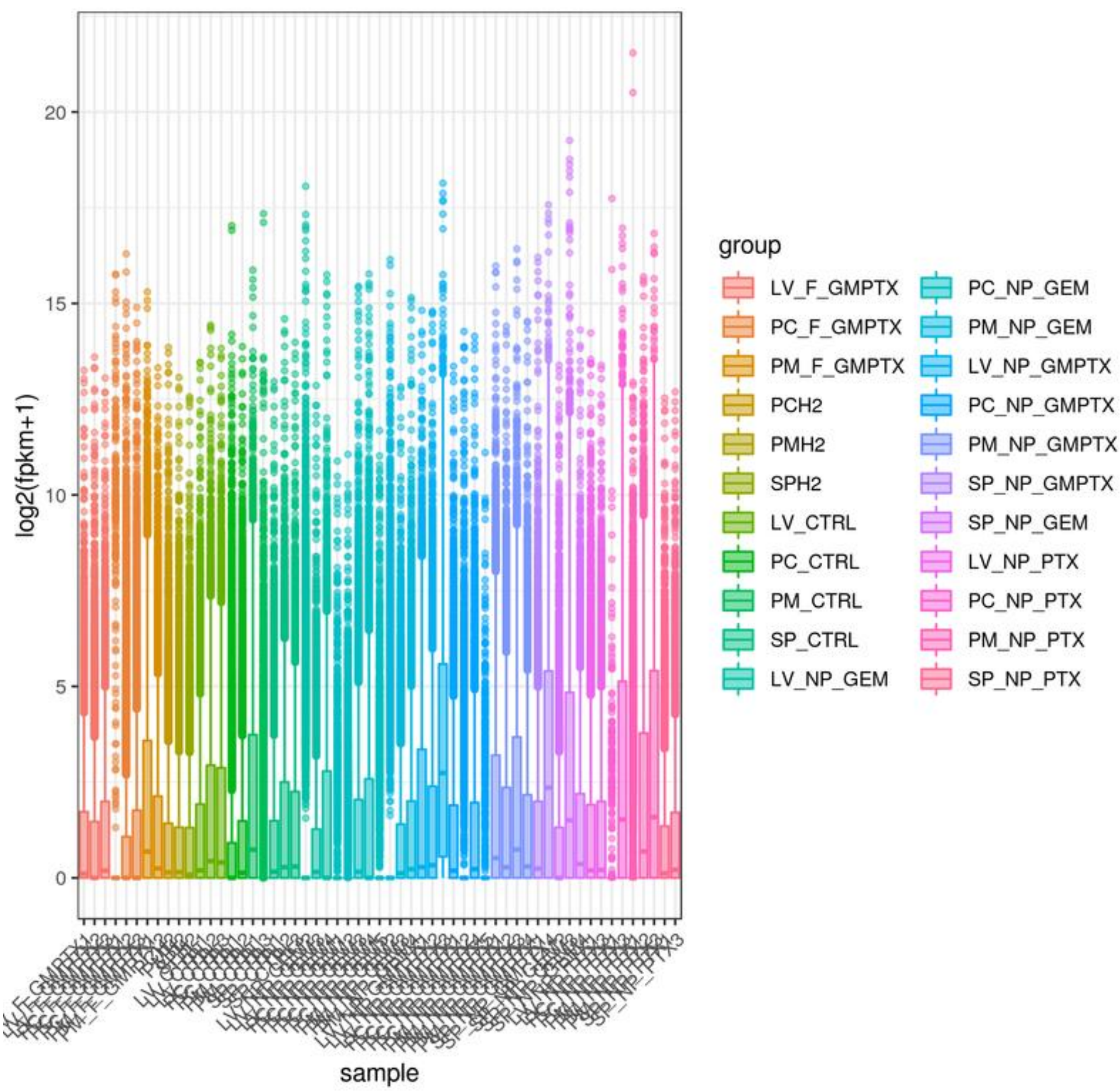

Fig S28. Boxplot showing the distribution of log2-transformed gene expression levels across different sample groups. Each colour represents a unique treatment or control group, with the vertical spread of each boxplot indicating the interquartile range of expression within each group.

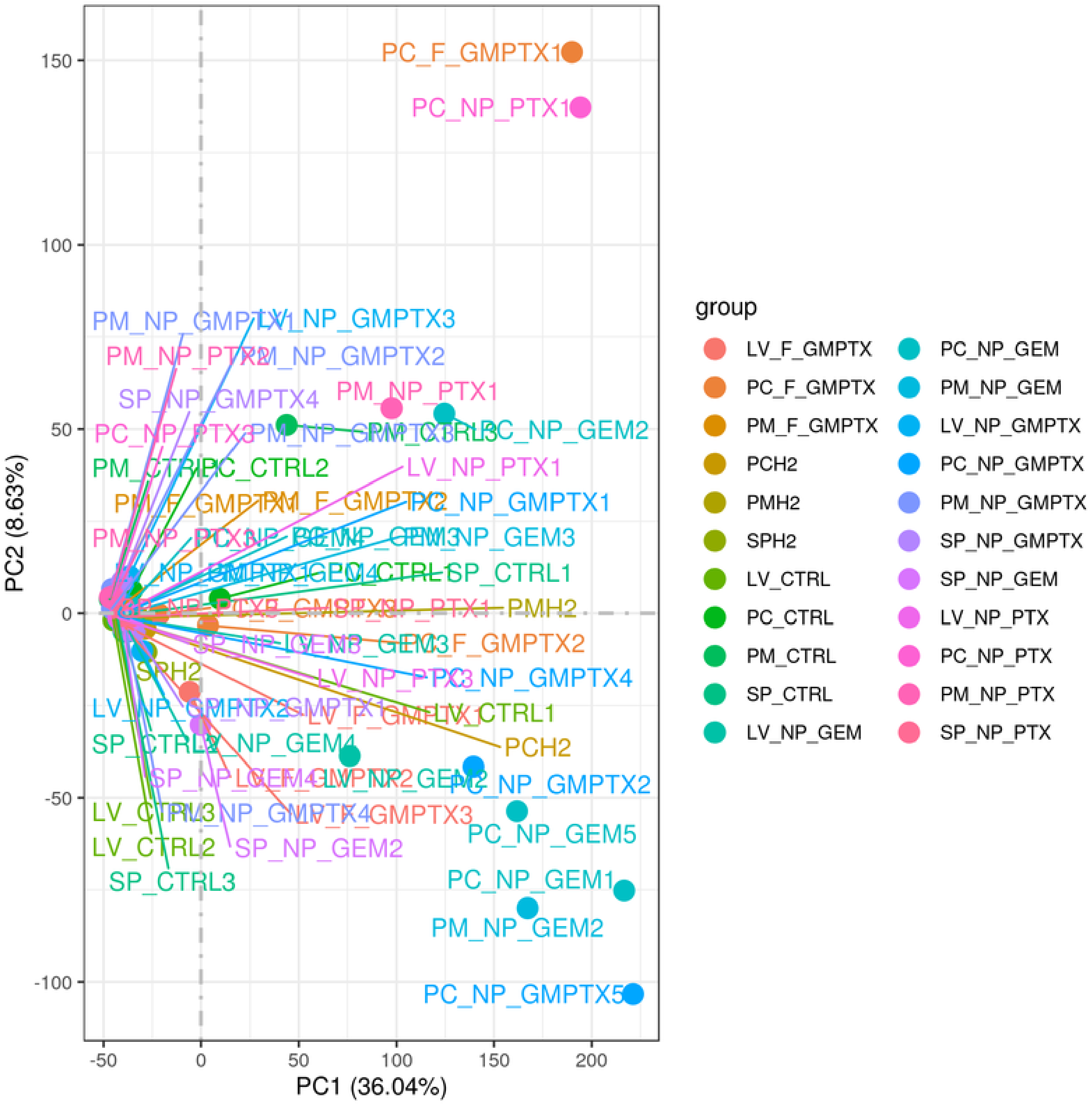

Fig. S29. Two-dimensional Principal Component Analysis (PCA) scatter plot demonstrating the variation and clustering of different sample groups based on their gene expression profiles. Each point represents a sample, with the colour coding corresponding to different treatment groups, and the axes represent the first two principal components that explain the largest amount of variance.

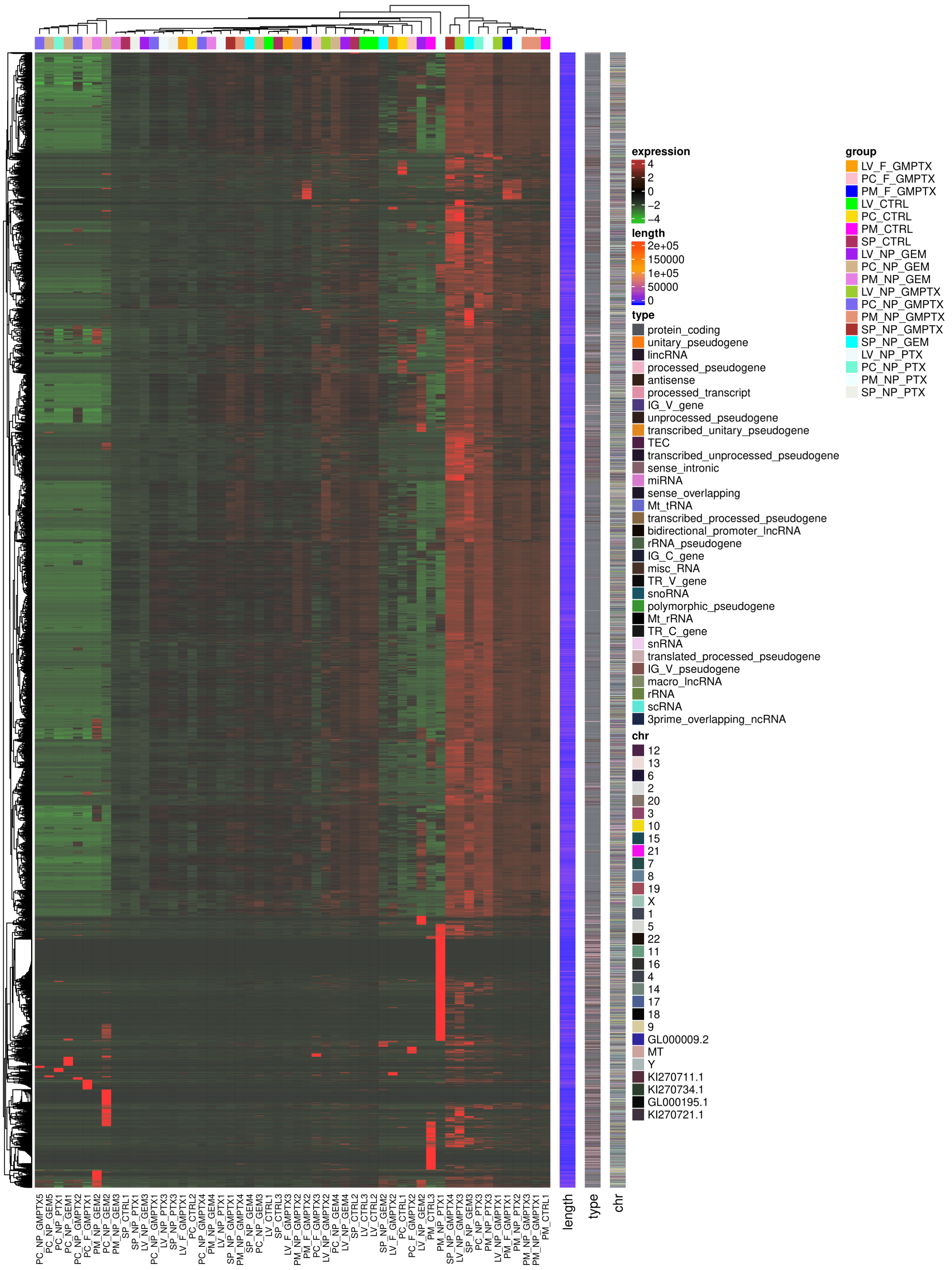

Fig. S30. Heatmap illustrating the differential expression of genes across all sample groups with varying gene types and chromosomes. The colour gradient from green to red indicates the expression level, with red denoting a higher expression and green denoting a lower expression. The clusters at the top correspond to different treatment conditions, whereas the side bars categorise genes by type and chromosomal location.

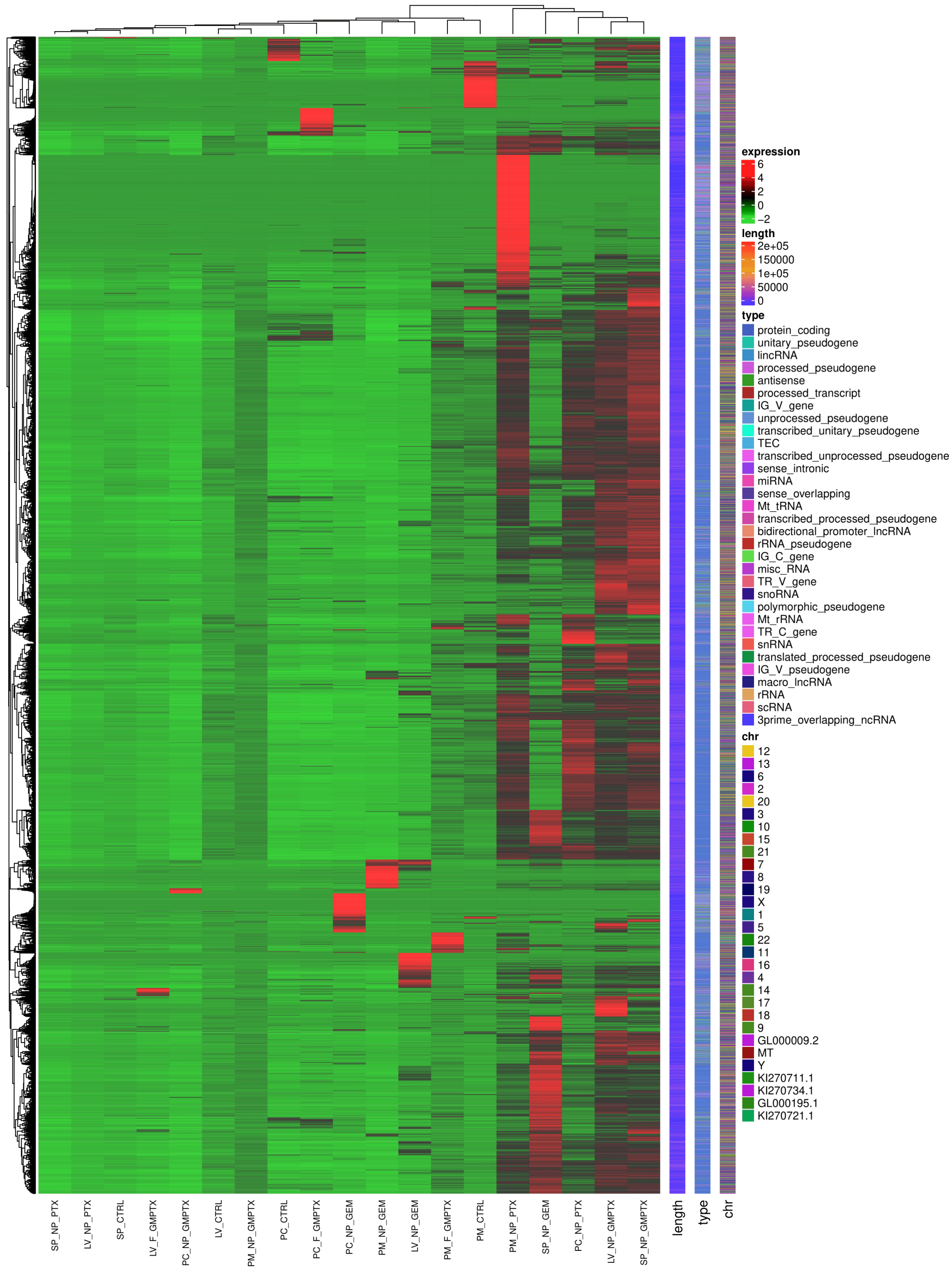

Fig. S31. Gene expression heatmap for sample groups under different treatment conditions, with red indicating higher expression levels and green showing lower expression levels. Gene categories and chromosomal locations are delineated by coloured side bars, providing insights into the genomic distribution of expression changes.

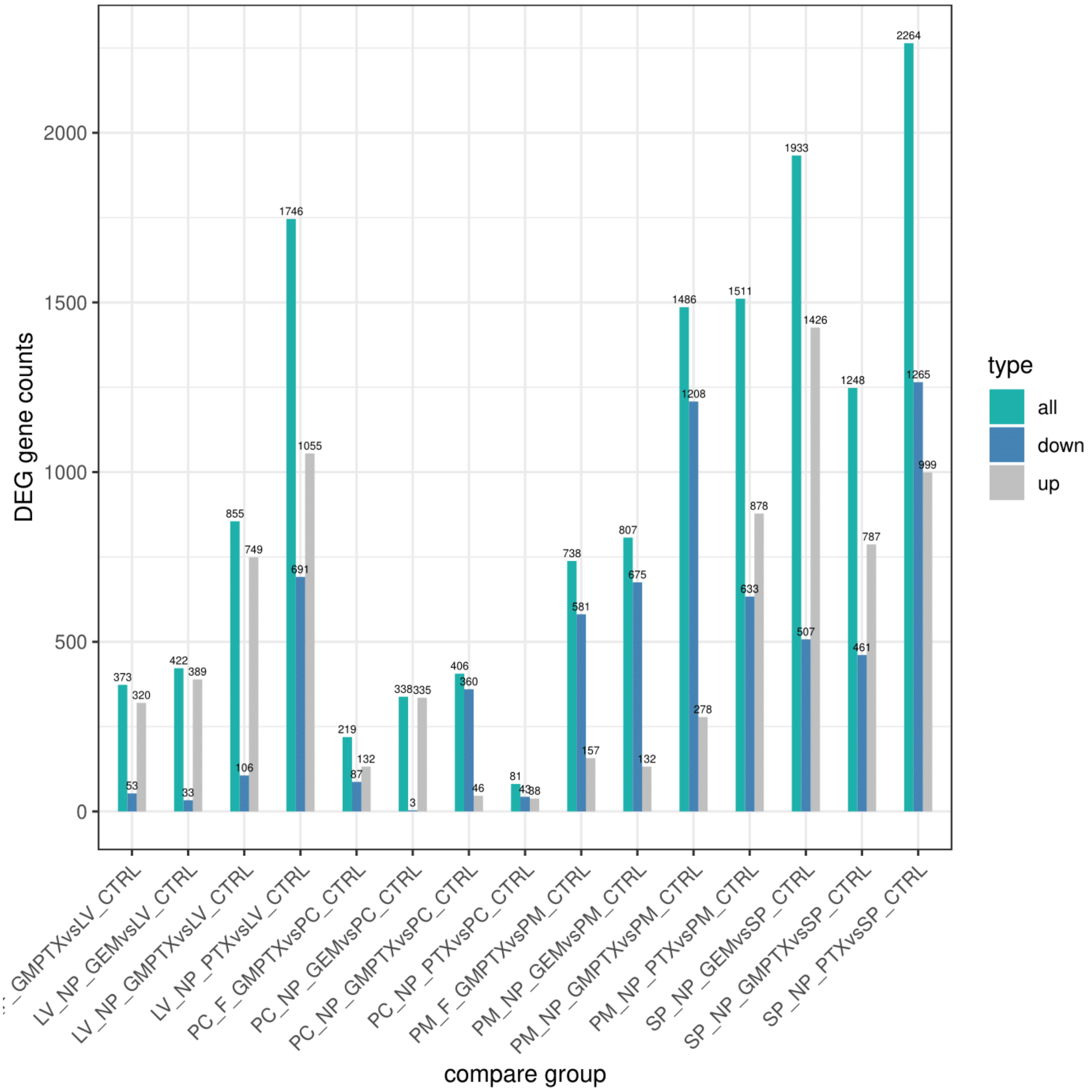

Fig. S32. Bar chart representing the number of differentially expressed genes (DEGs) across various treatment comparison groups, categorised by upregulated (light blue), downregulated (dark blue), and total DEGs (grey). Numbers above the bars indicate the number of DEGs in each category.

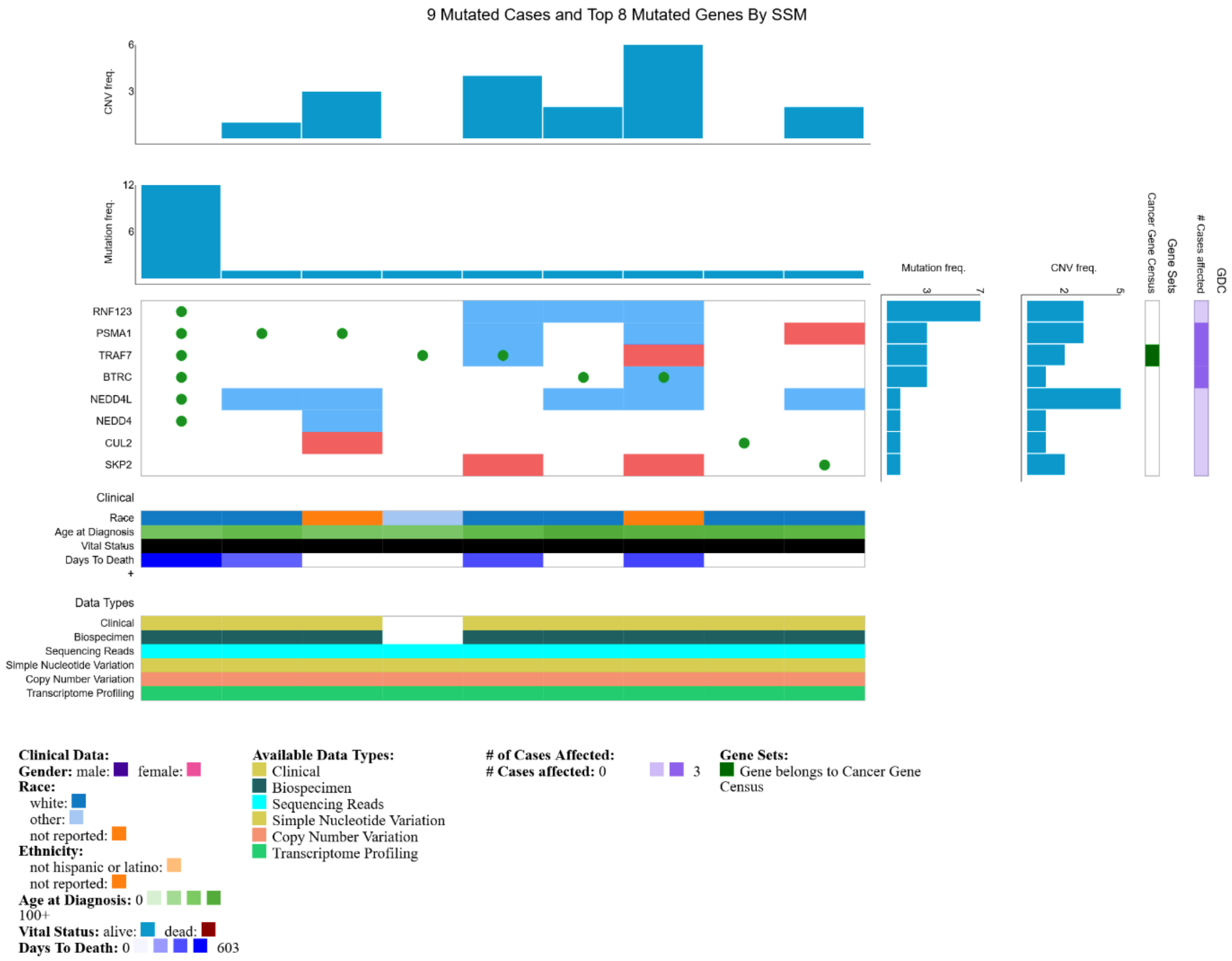

Fig. S33. Oncoprint visualisation of genetic alterations in key ubiquitination and proteasome pathway genes in pancreatic cancer. This Oncoprint from The Cancer Genome Atlas (TCGA) provides an overview of genetic alterations in key genes involved in ubiquitination and proteasome pathways in a cohort of pancreatic cancer cases. It details somatic single nucleotide mutations, copy number variations (CNVs), and clinical data such as patient age at diagnosis, vital status, and race. Green dots represent mutations, whereas colour blocks in the heatmap correspond to CNVs, with the legend indicating amplifications (red) and deletions (blue). The right side of the Oncoprint summarises the mutation and CNV frequencies across the cohort for each gene. This Oncoprint provides a snapshot of the genomic landscape of *RNF123*, *PSMA1*, *TRAF7*, *BTRC*, *NEDD4L*, *NEDD4*, *CUL2*, and *SKP2* in pancreatic cancer. These genes play critical roles in ubiquitination and proteasome degradation pathways, which are integral to protein homeostasis, and can influence cancer development and progression through the regulation of protein degradation. The presence of somatic mutations and CNVs in these genes among the cases indicates their potential involvement in pancreatic tumourigenesis or progression.

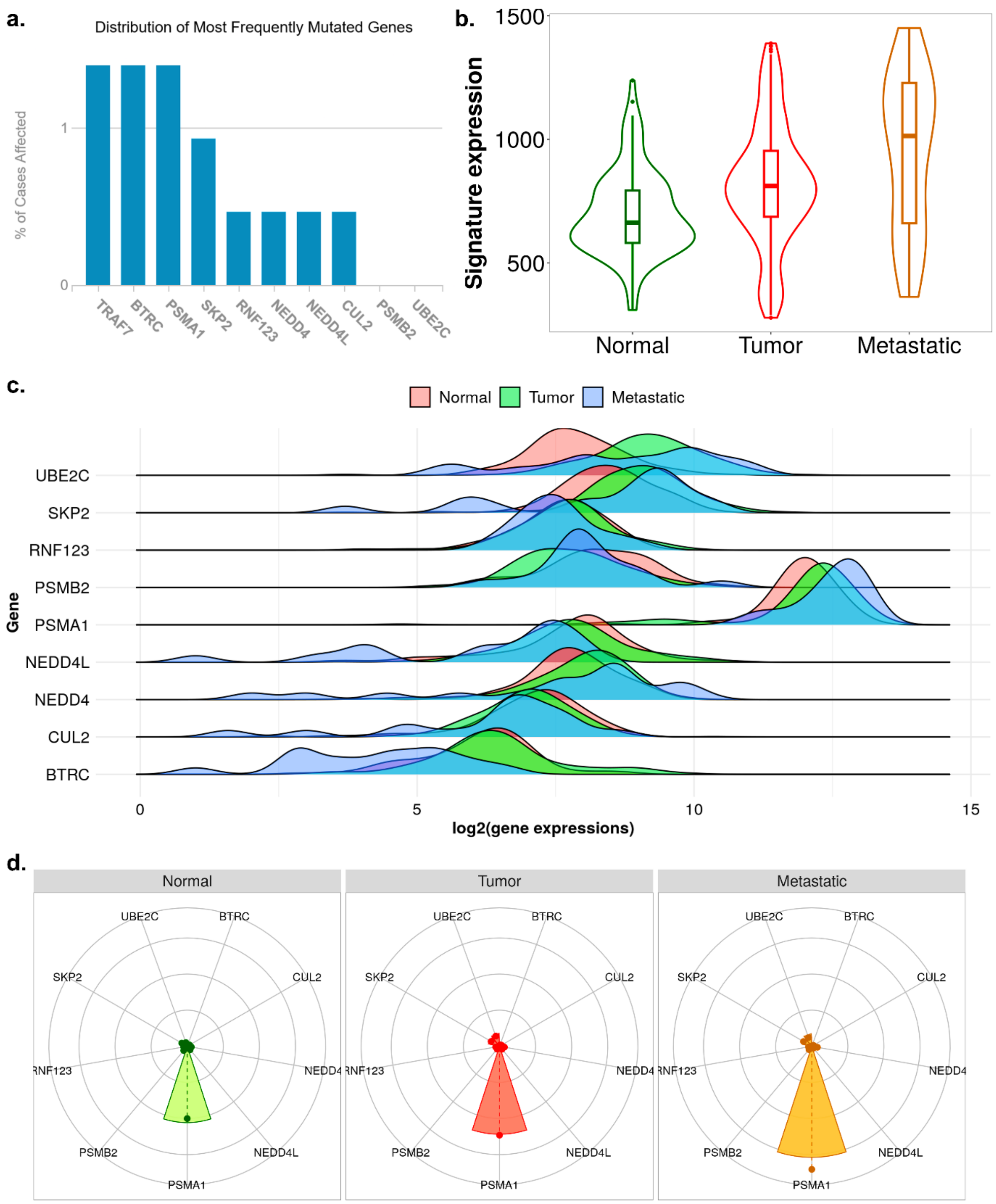

Fig. S34. Genetic alterations and expression profiles of ubiquitination and proteasome pathway genes in pancreatic cancer progression. Multi-panel overview of genetic and expression data related to key genes in ubiquitination and proteasome pathways across different stages of pancreatic cancer. **a.** distribution of the most frequently mutated genes in this pathway across cancer cases. The frequency of these mutations underscores the potential importance of these genes in tumourigenesis and tumour progression. **b.** comparison of the signature expression levels of these genes between normal, primary tumour, and metastatic tissues. Violin plots revealed a clear trend of increased signature expression of these genes in tumour and metastatic tissues compared to that in normal tissues, suggesting that the ubiquitin-proteasome system could be increasingly active or dysregulated as the disease progresses. This

increased expression may contribute to cancer development by affecting the degradation of the regulatory proteins involved in cell growth and survival. **c.** ridge plots detailing the distribution of log2-transformed gene expressions for individual genes across the same tissue types. The distribution of expression levels for genes such as *PSMB2* and *PSMA1* is particularly significant, with a pronounced shift towards higher expression in tumour and metastatic tissues. This shift could be reflective of increased protein turnover in cancer cells and may represent a potential target for therapeutic intervention. **d.** radar plots showcasing the relative expression levels of these genes in normal, tumour, and metastatic samples. The radial distance from the centre indicates the level of gene expression. The plots show a distinct pattern for each gene, with some genes, such as *PSMA1* and *PSMB2*, exhibiting a marked increase in expression in the tumour and metastatic samples, which is consistent with the ridge plots.

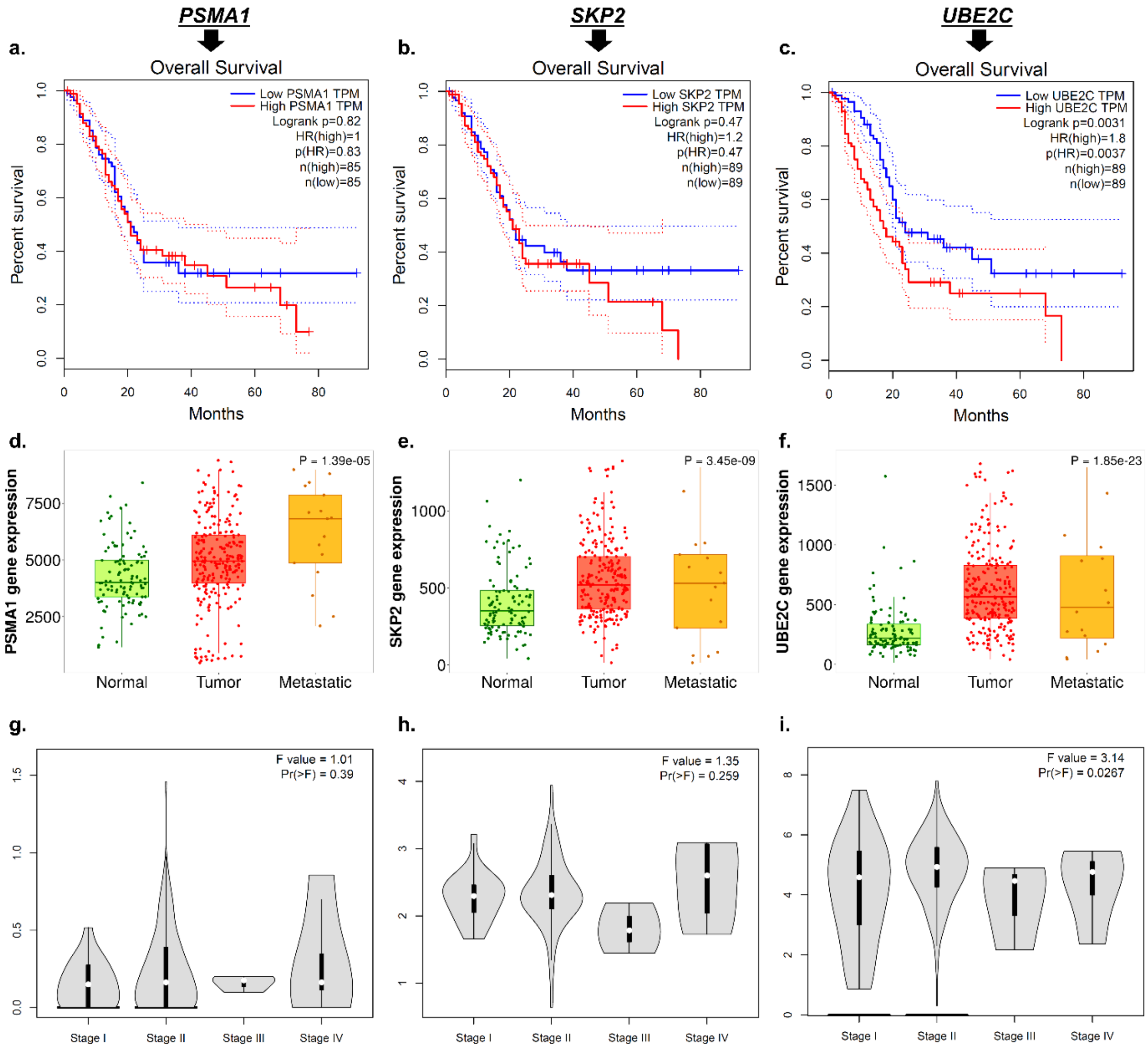

Fig. S35. Survival outcomes and gene expression analysis of *PSMA1*, *SKP2*, and *UBE2C* in pancreatic cancer progression. We analysed the association between *PSMA1*, *SKP2*, and *UBE2C* expression and patient survival in pancreatic cancer, along with the differential expression of these genes in normal, primary tumour, and metastatic contexts. **a-c.** Kaplan-Meier survival curves for patients stratified by high and low expression levels of *PSMA1*, *SKP2*, and *UBE2C* showed that high expression of *PSMA1* and *UBE2C* was associated with significantly lower overall survival, as indicated by the log-rank p value and hazard ratios. **d-f.** Boxplots illustrating gene expression across normal, tumour, and metastatic samples provide evidence of increased expression of *PSMA1*, *SKP2*, and *UBE2C* in tumour tissues compared to that in normal tissues, with the expression generally being the highest in metastatic samples. This upregulation trend in cancerous tissues reinforces the notion that these genes may play a role in tumour growth, maintenance, and metastasis. **g-i.** Violin plots of gene expression distribution across different cancer stages illustrated that while the expression of *PSMA1* and *SKP2* did not vary significantly across stages, *UBE2C* showed significant variation, with stage IV cancers exhibiting higher expression levels. This aligns with the survival analysis and suggests that *UBE2C* may be particularly relevant in late-stage pancreatic cancer.
